# Supplementary material for: Investigation of the optimal platinum-based regimen in the postoperative adjuvant chemotherapy setting for early-stage resected non-small lung cancer: a Bayesian network meta-analysis
Source: BMJ Open. 2022 Jun 12;12(6):e057098. doi: 10.1136/bmjopen-2021-057098 (PMC9196189; doi:10.1136/bmjopen-2021-057098)
Supplement: Supplementary data [file bmjopen-2021-057098supp002.pdf]

Supplementary.Material.2 The detailed searching strategy

*PubMed:

("Carcinoma, non-small cell lung" OR "Lung carcinoma, non-small-cell" OR "Carcinoma, non-small cell lung" OR "Non-small cell lung cancer")

AND ("adjuvant chemotherapy" OR "chemotherapy, adjuvant")

AND ("platinum" OR "cisplatin" OR "carboplatin" or "nedaplatin" or "oxaliplatin")

*EMBASE, and The Cochrane Library, Web of Science and Scopus Google Scholar:

("Carcinoma, non-small cell lung" OR "Lung carcinoma, non-small-cell" OR "Carcinoma, non-small cell lung" OR "Non-small cell lung cancer")

AND ("postoperative adjuvant chemotherapy")

AND ("platinum" OR "cisplatin" OR "carboplatin" or "nedaplatin" or "oxaliplatin")

In addition, searching performed in the database of Web of Science and Scopus Google Scholar was constrained to titles and abstracts while no limitations were applied in the PubMed, EMBASE, and The Cochrane Library.
