# Supplementary material for: Investigation of the optimal platinum-based regimen in the postoperative adjuvant chemotherapy setting for early-stage resected non-small lung cancer: a Bayesian network meta-analysis
Source: BMJ Open. 2022 Jun 12;12(6):e057098. doi: 10.1136/bmjopen-2021-057098 (PMC9196189; doi:10.1136/bmjopen-2021-057098)
Supplement: Supplementary data [file bmjopen-2021-057098supp003.pdf]

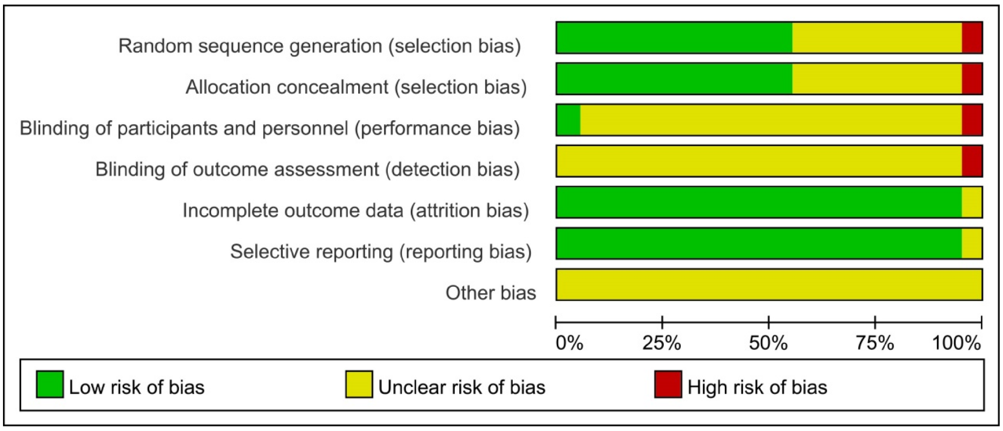

Supplementary Figure 1. A) Risk of bias graph.

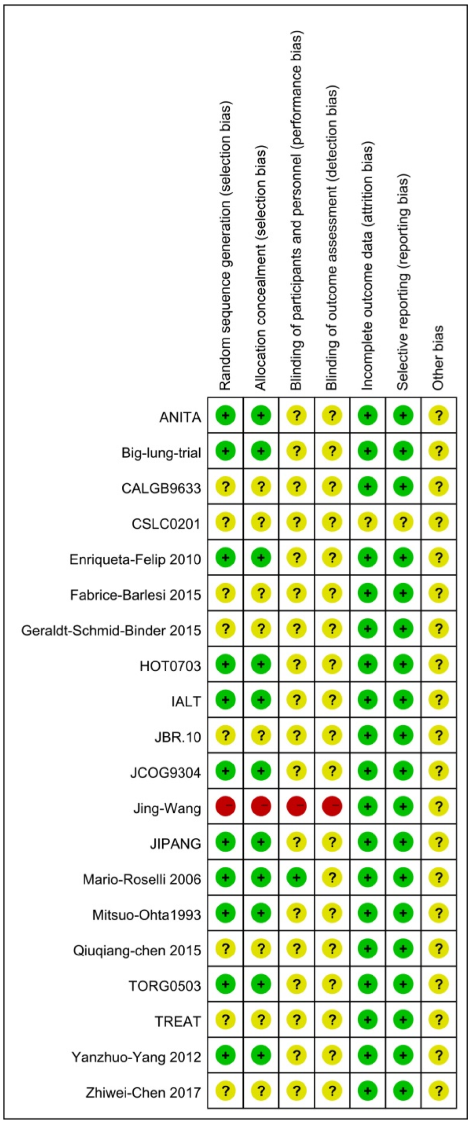

B) Risk of bias summary

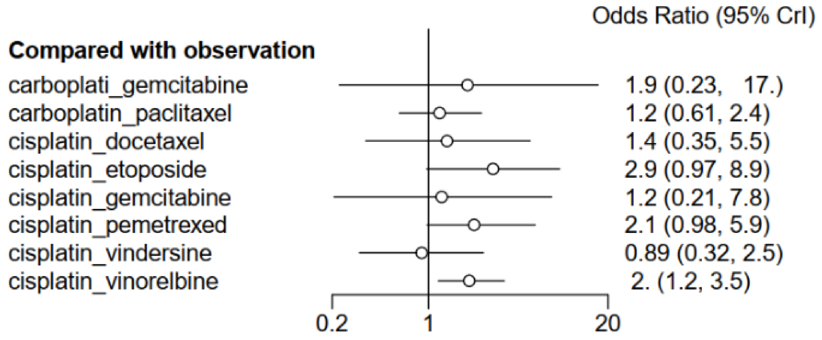

Supplementary.Figure 2.

A) Forest plots of the comparisons for the different cytotoxicity chemotherapy regimens with regard to 2-year RFS rate.

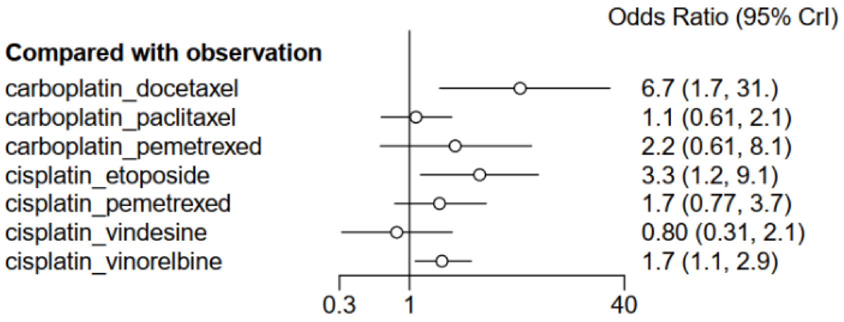

B) Forest plots of the comparisons for the different cytotoxicity chemotherapy regimens with regard to 3-year RFS rate.

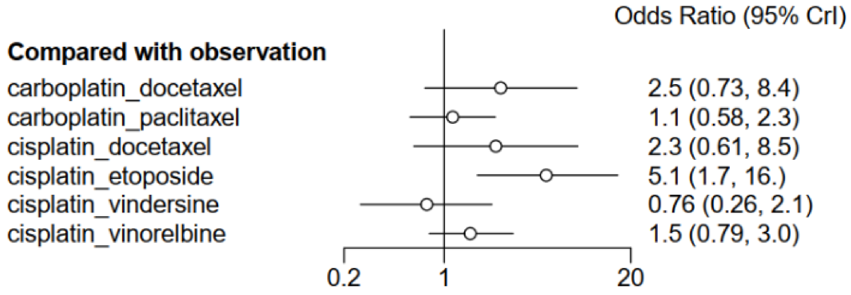

C) Forest plots of the comparisons for the different cytotoxicity chemotherapy regimens with regard to 5-year RFS rate.

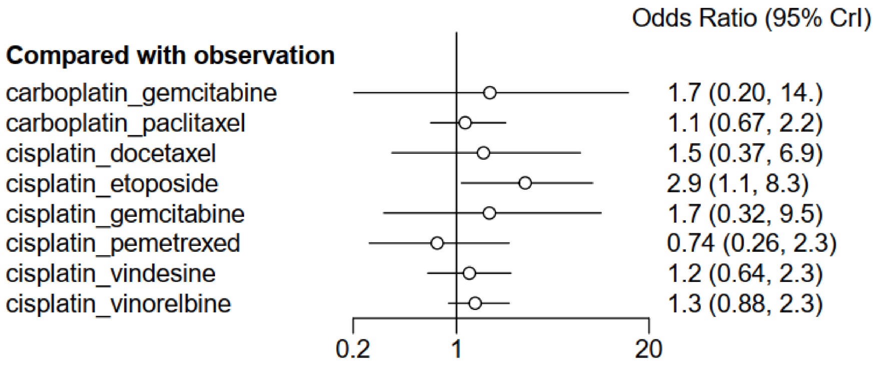

Supplementary.Figure 3.

A) Forest plots of the comparisons for the different cytotoxicity chemotherapy regimens with regard to 2-year OS rate.

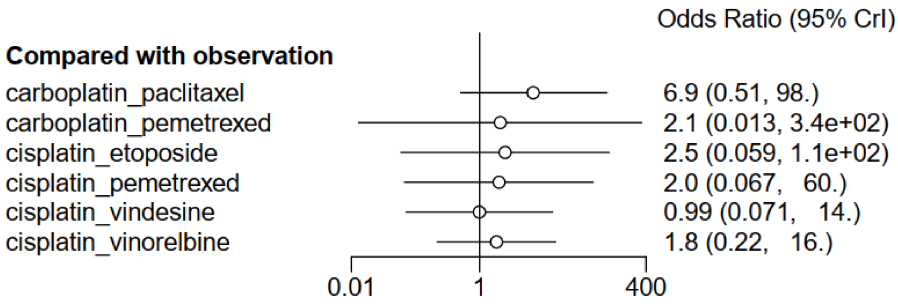

B) Forest plots of the comparisons for the different cytotoxicity chemotherapy regimens with regard to 3-year OS rate.

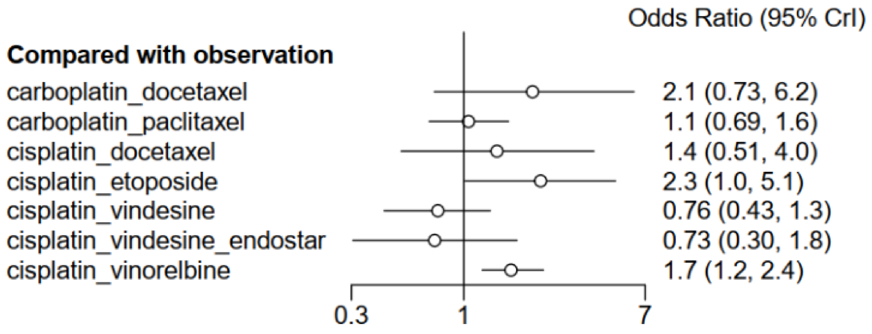

C) Forest plots of the comparisons for the different cytotoxicity chemotherapy regimens with regard to 5-year OS rate.
