# Supplementary material for: Investigation of the optimal platinum-based regimen in the postoperative adjuvant chemotherapy setting for early-stage resected non-small lung cancer: a Bayesian network meta-analysis
Source: BMJ Open. 2022 Jun 12;12(6):e057098. doi: 10.1136/bmjopen-2021-057098 (PMC9196189; doi:10.1136/bmjopen-2021-057098)
Supplement: Author's manuscript [file bmjopen-2021-057098.draft_revisions.pdf]

BMJ Open

BMJ Open is committed to open peer review. As part of this commitment we make the peer review history of every article we publish publicly available.

When an article is published we post the peer reviewers' comments and the authors' responses online. We also post the versions of the paper that were used during peer review. These are the versions that the peer review comments apply to.

The versions of the paper that follow are the versions that were submitted during the peer review process. They are not the versions of record or the final published versions. They should not be cited or distributed as the published version of this manuscript.

BMJ Open is an open access journal and the full, final, typeset and author-corrected version of record of the manuscript is available on our site with no access controls, subscription charges or pay-per-view fees (http://bmjopen.bmj.com).

If you have any questions on BMJ Open's open peer review process please email info.bmjopen@bmj.com

BMJ Open

Investigation of the Optimal Platinum-based Regimen in the Postoperative Adjuvant Chemotherapy Setting for Early-stage Resected Non-small Lung Cancer: A Bayesian Network Meta-analysis

Journal:	*BMJ Open*
Manuscript ID	bmjopen-2021-057098
Article Type:	Original research
Date Submitted by the Author:	08-Sep-2021

Complete List of Authors:	pang, lanlan; Sun Yat-sen University Cancer Center, Department of Medical Oncology<br>Gan, Jiadi; Sun Yat-sen University Cancer Center, Department of Medical Oncology<br>Huang, Yi-Hua; Sun Yat-sen University Cancer Center, Department of Medical Oncology<br>Liao, Jun; Sun Yat-sen University Cancer Center, Department of Medical Oncology<br>Lv, Yi; Sun Yat-Sen University, Zhongshan School of Medicine<br>Ali, Wael; Sun Yat-sen University Cancer Center, Department of Medical Oncology<br>Zhang, L ; Sun Yat-sen University Cancer Center, Department of Medical Oncology<br>Fang, Wenfeng; Sun Yat-sen University Cancer Center, Department of Medical Oncology

Keywords:	Adult oncology < ONCOLOGY, Respiratory tract tumours < ONCOLOGY, Thoracic surgery < SURGERY

SCHOLARONE™
Manuscripts

I, the Submitting Author has the right to grant and does grant on behalf of all authors of the Work (as defined in the below author licence), an exclusive licence and/or a non-exclusive licence for contributions from authors who are: i) UK Crown employees; ii) where BMJ has agreed a CC-BY licence shall apply, and/or iii) in accordance with the terms applicable for US Federal Government officers or employees acting as part of their official duties; on a worldwide, perpetual, irrevocable, royalty-free basis to BMJ Publishing Group Ltd ("BMJ") its licensees and where the relevant Journal is co-owned by BMJ to the co-owners of the Journal, to publish the Work in this journal and any other BMJ products and to exploit all rights, as set out in our licence.

The Submitting Author accepts and understands that any supply made under these terms is made by BMJ to the Submitting Author unless you are acting as an employee on behalf of your employer or a postgraduate student of an affiliated institution which is paying any applicable article publishing charge ("APC") for Open Access articles. Where the Submitting Author wishes to make the Work available on an Open Access basis (and intends to pay the relevant APC), the terms of reuse of such Open Access shall be governed by a Creative Commons licence – details of these licences and which Creative Commons licence will apply to this Work are set out in our licence referred to above.

Other than as permitted in any relevant BMJ Author's Self Archiving Policies, I confirm this Work has not been accepted for publication elsewhere, is not being considered for publication elsewhere and does not duplicate material already published. I confirm all authors consent to publication of this Work and authorise the granting of this licence.

For peer review only

Investigation of the Optimal Platinum-based Regimen in the Postoperative Adjuvant Chemotherapy Setting for Early-stage Resected Non-small Lung Cancer: A Bayesian Network Meta-analysis

Running title: Postoperative Platinum-based Adjuvant Chemotherapy Setting for NSCLC

Author:

Lan-Lan Pang,MD[1], Jia-Di Gan,MD[1], Yi-Hua Huang,MD[1], Jun Liao,MD[1], Yi Lv, MD[2], Wael-Abdullah -Sultan Ali, MD[1], Li Zhang, MD , Phd[1], Wen-Feng Fang, MD ,Phd[1#]

Institutions:

[1]Department of Medical Oncology, State Key Laboratory of Oncology in South China, Collaborative Innovation Center for Cancer Medicine, Sun Yat-sen University Cancer Center, Guangzhou 510060, People's Republic of China

[2] Zhongshan School of Medicine, Sun Yat-sen University, 74 Zhongshan II Road, Guangzhou510080, People's Republic of China.

#Correspondence:

Wenfeng Fang, MD, Phd, Department of Medical Oncology, State Key Laboratory of Oncology in South China, Collaborative Innovation Center for Cancer Medicine, Sun Yat-sen University Cancer Center, Guangzhou 510060, Peoples Republic of China. Email: fangwf@sysucc.org.cn.

Acknowledgments:

For peer review only

This work was financially supported by the Chinese National Natural Science Foundation Project (81772476). We thank all the co-authors for their contributions to this study.

Formatting of funding sources:

This work was supported by the Chinese National Natural Science Foundation Project (81772476).

Conflict of Interest Statement:

The authors had no conflict of interest to declare.

For peer review only

Abstracts

Objective

Investigation of the optimal platinum regimen in the postoperative adjuvant chemotherapy setting for early-stage resected non-small-cell lung cancer (NSCLC) is of great significance for the oncological community in routine clinical practice. However, it seems difficult to conduct large head-to-head randomized controlled trials (RCTs) to figure out the preferred platinum chemotherapy regimen, and utilize the published data might be an alternative option. This meta-analysis was aimed to compare the efficacy and safety of different platinum adjuvant chemotherapy regimens.

Methods

Eligible RCTs comparing the postoperative platinum chemotherapy regimen with the observation-controlled group or comparing two platinum chemotherapy regimens head to head were included. The primary outcome was the efficacy of adjuvant chemotherapy regimens including relapse-free survival (RFS), overall survival (OS), 2, 3, 5-year RFS rate, and OS rate. The secondary outcome were the rate of grade3-4 toxicity assessments. Analyses were performed utilizing R-software version 4.3.1.

Results

20 RCTs with a sample size of 5483 were enrolled in meta-analysis. The chemotherapy group had a significant RFS and OS advantage compared to the observation group [hazard ratio (HR), 0.67; 95% confidence interval (CI), 0.56 to 0.81, $P<0.0001$; HR, 0.80; 95% CI, 0.73 to 0.88, $P<0.0001$, respectively]. Compared

For peer review only

with the observation arm, only the “Cisplatin+Vinorelbine” regimen had a significant RFS and OS advantage (HR, 0.63; 95%CI, 0.43 to 0.87; HR, 0.74; 95%CI, 0.63 to 0.87, respectively) while the other chemotherapy regimens had no significant difference of efficacy compared to the observation group. In terms of the safety of adjuvant chemotherapy, the incidence of hematological toxicities and nausea/vomiting was not significantly higher in the “Cisplatin+Vinorelbine” arm than in other chemotherapy.

Conclusion

The current study summarized the adjuvant cytotoxicity chemotherapy regimens for patients with early-stage resected NSCLC. Our analysis may bring some guiding significance to the clinicians when they make clinical decisions of the optimal chemotherapy regimen.

Keywords

Non-small cell lung cancer, adjuvant chemotherapy, platinum-based, Network Meta-analysis

Trial registration: None

Strengths and limitations of this study

* The current systematic review and Bayesian network meta-analysis was the first study to compare the efficacy and safety of different adjuvant chemotherapy regimens directly for NSCLC patients.

For peer review only

* Researches on adjuvant cytotoxicity chemotherapy might be an out-of-date topic but numerous NSCLC patients could obtain benefit from the optimal cytotoxicity chemotherapy regimen. "

* Cisplatin+Vinorelbine" had a significant survival advantage with a relatively good safety profile in the adjuvant setting while the "Cisplatin+Pemetrexed" arm was not superior to the other therapeutic methods in improving survival.

* It is difficult to determine the optimal subgroup of patients who may obtain the benefit from certain cytotoxicity chemotherapy regimens.

* We did not incorporate any targeted or immune-biological therapy into the analysis due to the open-loop of interventions.

For peer review only

Introduction:

Lung cancer is the most common cause of cancer-related deaths worldwide and patients with non-small lung cancer (NSCLC) account for almost 85% of all cases [1, 2]. Complete surgical resection is recognized as the standard treatment for patients with early-stage NSCLC. However, 30% to 70% of patients ultimately experience local or distant relapse and the five-year survival rate only ranges from 30% to 60% [3, 4]. The presence of micrometastases, which could not be detected by conventional diagnostic techniques, increases the rate of relapse and exerts a negative impact on the survival of patients [5]. Therefore, adjuvant postoperative systematic therapy is necessary for patients with surgical resection.

A pooled analysis conducted by the Lung Adjuvant Cisplatin Evaluation (LACE) Collaborative Group suggested that cisplatin-based adjuvant chemotherapy yield an absolute OS benefit of 5.40% at five years [6]. In light of this promising data, the American Society of Clinical Oncology (ASCO) guidelines recommended the cisplatin-based adjuvant postoperative chemotherapy regimen as the standard treatment for early-stage NSCLC patients [7]. However, the optimal adjuvant chemotherapy regimen has not yet been determined. “Cisplatin+Vinorelbine” has shown its promising efficacy compared with the observation group and is recognized as the standard adjuvant chemotherapy regimen currently [8, 9]. In addition, the feasibility, efficacy, and safety of cisplatin plus non-vinorelbine third-generation drug as the postoperative adjuvant chemotherapy also have been investigated by several RCTs [10-12]. Among them, “Cisplatin+Pemetrexed” is characterized as having

For peer review only

promising efficacy and an acceptable safety profile in comparison with “Cisplatin+ vinorelbine” [12, 13]. Thus, despite the lack of level 1 data concerning the efficacy of cisplatin plus vinorelbine, it was still recommended by the National Comprehensive Cancer Network (NCCN) guidelines as the optional postoperative adjuvant chemotherapy regimen for non-squamous NSCLC [14]. Meanwhile, although carboplatin-based adjuvant regimens have yet been recommended by guidelines, it might be a favorable choice for patients with comorbidities who were unsuited to receive cisplatin when accompanied by the late toxicity [15, 16].

Recent advances in individualized treatment based on molecular and biological profiling have shaped the future of postoperative adjuvant chemotherapy. Substantial numbers of patients benefit from the corresponding adjuvant regimen. The patients unsuited to adjuvant cytotoxicity chemotherapy may benefit from the first-generation Tyrosine Kinase Inhibitor (TKI) but serval clinical trials showed poor outcomes of TKI adjuvant therapy until the advent of ADJUVANT study [17-19]. However, individualization could not apply to all NSCLC patients and those patients inapplicable to it have no choice but receive platinum adjuvant chemotherapy.

Postoperative adjuvant chemotherapy setting for early-stage resected NSCLC is of great significance for the oncological community in routine clinical practice.

However, it seems difficult to conduct large RCT to figure out the preferred platinum chemotherapy regimen, and utilize the published data might be an alternative option.

Hence, we conducted this systematic review and network meta-analysis aiming to

For peer review only

compare the efficacy and safety of different platinum adjuvant chemotherapy regimens.

Methods:

This systematic review and network meta-analysis was performed in accordance with the Preferred Reporting Items for Systematic reviews and Meta-analyses guidelines for RCTs (The PRISMA Statement) (**Supplementary material 1**).

Data Sources

Two authors (L.L.P and J.D.G) independently searched the records in the electronic database of PubMed, EMBASE, and The Cochrane Library, Web of Science and Scopus Google Scholar. The searching terminal date was March 12th, 2021. Searching terms were focused on NSCLC, adjuvant chemotherapy, and platinum. If necessary, an additional manual search of related literature in the reference list would be carried out to enroll any relevant publications. The datasets utilized in this analysis could be obtained from the corresponding author upon request. Records were imported into EndnoteX9 software to eliminate duplications. The detailed strategy was presented in **Supplementary material 2**.

Trial Selection Criteria and Trial Identification

Two authors (L.L.P and Y.H.H) independently reviewed the title, abstracts, and keywords of identified citations to select appropriate articles for a full review. Any disagreement was resolved by consensus or through the judgement of a senior author (W.F.F). Trials would be eligible only if meeting all the following criteria: 1) patients with completely resected NSCLC (squamous and non-squamous) at stage IB–IIIA; 2)

For peer review only

Eligible RCTs comparing the postoperative platinum chemotherapy regimen with observation-controlled group or those including two platinum chemotherapy regimens head-to-head comparison were included; the counterpart of the platinum doublet included Vinorelbine, Etoposide, Pemetrexed, Docetaxel, Paclitaxel, Gemcitabine, Vindesine; a platinum triplet had to be a platinum doublet combined with anti-angiogenesis drug; 3) full-text publications or conference abstract ;4) non-language restrictions. Publications would be disregarded if meeting any of the following criteria: 1) any single perioperative chemotherapy, neo-adjuvant chemotherapy, maintenance chemotherapy, or radio-chemotherapy; 2) chemo-naive patients with advanced, incurable, or recurrent NSCLC; 3) not RCTs including reviews, case series or retrospective trials.

Outcomes and Data Extraction

Two authors (L.L.P and J.L) independently performed data extraction and any discrepancies were eliminated by consensus. Data for the eligible trials related to basic characteristics were extracted including: trial/author name, publication year, country of origin/multi-centers, sample size per group, chemotherapy regime, administration dose of each treatment and number of cycles, follow-up time, phases II/III, basic patients' characteristics (age, sex ratio, stage, pathology, performance status).

The primary outcome was the efficacy of adjuvant chemotherapy regimens including RFS, OS, 2, 3, 5-year RFS rate, and OS rate. RFS was defined as the interval from day of surgeryto disease recurrence or death, whichever occurred first, while OS

For peer review only

referred to the time from sugery to death from any cause.

The secondary outcomes were the rate of grade3-4 toxicity assessment, including hematological (anemia, neutropenia, febrile neutropenia, and thrombocytopenia) and non-hematological (nausea, vomiting) adverse events. No additional information was requested from the authors. Also, Parmar's method would be utilized to obtain survival if necessary.

Quality and Risk of Bias Assessment

Two researchers (L.L.P and J.D.G) independently assessed the risk of bias of enrolled trials according to the recommendations of the Cochrane Handbook of Systematic Reviews of Interventions (http://handbook.cochrane.org). Disagreements were resolved by discussion.

Data Synthesis and Analysis

For RFS and OS, the logarithm of hazard ratio (HR) and their standard error (SE) were pooled into analysis through a Bayesian multiple treatment network meta-analysis with random effects. As for the dichotomous variables, odds ratio (OR) with 95% confidence interval (CI) was applied to calculate. When a network diagram indicated two or more independent loops, only the loop containing "Cisplatin+Vinorelbine" was selected for further analysis.

Random effects and consistency model was computed utilizing Markov chain Monte Carlo methods with Gibbs sampling based on simulations of 50000 iterations and 20000 adaptions in each of 4 chains. For a forest plot, "observation" was chosen as the common reference comparator in the analysis of RFS and OS while

For peer review only

"Cisplatin+Vinorelbine" was applied in the analysis of chemotherapy toxicity. A league table for the survival analysis was presented with the logarithm of HR and their 95% CI. Probability values were summarized and presented with the surface under the cumulative ranking (SUCRA) curve and with a rank gram plot to provide a hierarchy of treatments with consideration of both the location and the variance of all relative treatment effects. The SUCRA value would be 0 if treatment is certain to be the worst and 1 if it is certain to be the best.

All analyses in this article were performed utilizing R-software version 4.3.1 and the gemtc package version 0.8 that interfaces with JAGS version 4.3.0 for computing a Markov chain.

Patient and public involvement

No patient involved

For peer review only

Results

The search process, study characteristics, and quality assessment

A total of 3432 records were yielded after searching the database and 1991 records were left to be identified after removing the 1692 duplications. Then, 1905 irrelevant citations were eliminated by skimming their title, abstracts, and keywords, leaving 86 articles to be considered potentially eligible. 69 articles were further excluded for irrelevant to topic (n=45); non-RCTs (n=11); non-accessible outcome (n=9); non-accessible text (n=4); and 17 articles were enrolled into analysis after skimming their full-text. Additionally, 3 studies were included by browsing the references. Finally, 20 studies with a sample size of 5483 were enrolled into meta-analysis **(Figure 1)**.

Table 1 showed the basic characteristics of enrolled studies and participants. These studies were conducted in countries worldwide, most of which were Japan [20-24] and China [11, 25-27], followed by France [28, 29], Germany [30], Italy [31], USA [32], UK [33], and North Carolina [10]. Noteworthy, 3 of them were conducted in multi-countries [34-36]. 11 studies compared the postoperative platinum chemotherapy regimen with the observation-controlled group while the other 9 studies containing two platinum chemotherapy regimens head-to-head. Chemotherapy regimens included "Cisplatin+Vinorelbine", "Cisplatin+Pemetrexed", "Cisplatin+Gemcitabine", "Cisplatin+Vindesine", "Cisplatin+Etoposide", "Cisplatin+Docetaxel", "Carboplatin+Gemcitabine", "Carboplatin+Pemetrexed", "Carboplatin+Paclitaxel", "Carboplatin+Docetaxel+Endostar",

For peer review only

"Carboplatin+Docetaxel", "Cisplatin+Vinorelbine+Endostar". The median follow-up time ranged from 20.2 to 116 months.

Based on the Cochrane Risk of Bias evaluation, one of the enrolled studies had a high risk of selection bias due to its quasi-randomized setting while nearly half the studies did not clarify the randomization methods in detail. One study had a high risk of performance bias while nearly all of the studies did not mention utilizing the blind design or not. Attrition and reporting bias did not exist in the enrolled studies except one whose bias could not be evaluated as a conference abstract (**Supplementary figure 1A-B**).

Primary outcome---RFS

10 studies were enrolled to analyze the RFS of platinum postoperative adjuvant chemotherapy compared with the observation group. We found that the chemotherapy group experienced a significant RFS advantage compared to the observation group (HR, 0.67; 95%CI, 0.56 to 0.81; I^2=64%, P<0.0001) **(Figure 2A)**. Moreover, in terms of 2-year RFS rate, 3-year RFS rate and 5-year RFS rate, the chemotherapy arm presented higher RFS rate in comparison with the observation arm (OR=1.62, 95%CI 1.20 to 2.18, I^2=68%, P=0.0016; OR=1.50, 95%CI 1.11 to 2.03, I^2=72%, P=0.0081; OR=1.49, 95%CI 1.09 to 2.04, I^2=69%, P=0.0132; respectively)**(Figure 2B-D)**.

15 clinical trials were enrolled to investigate the RFS of several chemotherapy regimens for patients with early-stage resected NSCLC. Network evidence of the comparisons for the best adjuvant chemotherapy concerning the RFS was shown in **Figure 3A**. Compared with the observation arm, only the "Cisplatin+Vinorelbine"

For peer review only

regimen had a significant RFS advantage (HR, 0.63; 95%CI, 0.43 to 0.87) while there was no significant difference between the other chemotherapy regimens and the observation group ---“Carboplatin+Docetaxel” (HR, 0.58; 95%CI, 0.24 to 1.4); “Carboplatin+Docetaxel+Endostar” (HR, 0.42; 95%CI, 0.05 to 3.8); “ Carboplatin+Paclitaxel” (HR, 0.88; 95%CI, 0.51 to 1.50); “Cisplatin+Docetaxel” (HR, 1.40; 95%CI, 0.49 to 3.90); “Cisplatin+Etoposide”(HR, 0.53; 95%CI, 0.23 to 1.30); “ Carboplatin+Pemetrexed” (HR, 0.62; 95%CI, 0.29 to 1.20); “Cisplatin+Vindesine” (HR, 1.00; 95%CI, 0.45 to 2.20); “Cisplatin+Vinorelbine+Endostar” (HR, 0.60; 95%CI, 0.24 to 1.40)(**Figure 3B**). In addition, according to the league table, there was no significant difference among these adjuvant chemotherapy regimens (**Figure 3C**). In the rank of network meta-analysis, the biggest SUCRA value holds the potential to be the best chemotherapy regime to improve RFS. Our results showed that “Cisplatin+Etoposide”(72.0%) was the most effective chemotherapy regime to improve the RFS, followed by “Carboplatin+Docetaxel+Endostar” (69.30%),“Carboplatin+Docetaxel”(65.31%),“Cisplatin+Vinorelbine”(64.73%),“Cisplatin+Pemetrexed”(64.2%),“Cisplatin+Vindesine”(64.2%),“Carboplatin+Paclitaxel”(36.3%),“Carboplatin+Vindesine”(27.6%),“Cisplatin+Vinorelbine+Endostar”(23.0%) and “Cisplatin+Docetaxel”(13.4%)(**Figure 3D**). In terms of the 2-year RFS rate, 3-year RFS rate and 5-year RFS rate, “Cisplatin+Vinorelbine” was an effective therapeutic method to improve 2-year RFS rate (OR=2.00, 95%CI 1.20 to 3.50); “Carboplatin+Docetaxel” (OR=6.70, 95%CI 1.70 to 31.00) “Cisplatin+Etoposide”

For peer review only

(OR=3.30, 95%CI 1.20 to 9.10) and "Cisplatin+Vinorelbine" (OR=1.70, 95%CI 1.10 to 2.90) had a significantly higher 3-year RFS rate than observation arm and "Cisplatin+Etoposide" (OR=5.10, 95%CI 1.70 to 16.00) was an effective method to improve the 5-year RFS rate. However, there was no significant difference among the other chemotherapy regimens and observation arm concerning the RFS rate (**Supplementary figure 2A-C**).

Primary outcome--- OS

To compare the efficacy of platinum chemotherapy arm with the observation arm in improving OS, we enrolled 10 studies into analysis. Our results showed that the OS in the chemotherapy group was significantly longer than the observation group (HR, 0.80; 95%CI, 0.73 to 0.88; I^2=11%, P<0.0001) (**Figure 4A**). As for 2-year OS rate, 3-year OS rate and 5-year OS rate, the chemotherapy group experienced a significant survival advantage than the observation group (OR=1.31, 95%CI 1.06 to 1.61, I^2=21%, P=0.0117; OR=2.24, 95% CI 1.13 to 4.45, I^2=94%, P=0.0081; OR=1.36, 95% CI 1.08 to 1.72, I^2=54%, P=0.0100; respectively)(**Figure 4B-D**).

Moreover, 14 clinical trials were enrolled to investigate the OS of several optimum adjuvant chemotherapy regimens. Network evidence of these chemotherapy regimens was shown in **Figure 5A**. Compared with the observation arm, only the "Cisplatin+Vinorelbine" regimen had a significant OS advantage (HR, 0.74; 95%CI, 0.63 to 0.87) while there was no significant difference between the other chemotherapy regimens and the observation group ---"Carboplatin+Paclitaxel" (HR,0.90; 95%CI, 0.70 to 1.20); "Cisplatin+Docetaxel" (HR, 1.10; 95%CI, 0.50 to

For peer review only

2.30) ; “Cisplatin+Etoposide” (HR, 0.80; 95% CI, 0.44 to 1.40); “Cisplatin+Pemetrexed”(HR, 0.73; 95%CI, 0.47 to 1.10) ; “Cisplatin+Vindesine” (HR, 1.00; 95%CI, 0.69 to 1.40); “Cisplatin+Vinorelbine+Endostar” (HR, 0.75; 95%CI,0.46 to 1.20) (**Figure 5B**). In addition, as the league table showed, there was no significant difference among these chemotherapy regimens in improving OS for patients with early-stage resected NSCLC (**Figure 5C**). In terms of the rank of network meta-analysis, we found that“Cisplatin+Vinorelbine” (74.8%) was the most effective therapeutic arm for the improvement of OS, followed by “Cisplatin+Pemetrexed”(74.1%), “Cisplatin+Vinorelbine+Endostar”(68.5%), “Cisplatin+Etoposide”(59.2%), “Carboplatin+Paclitaxel”(43.6%), “Cisplatin+Vindesine”(28.5%), “Cisplatin+Docetaxel”(27.0%) and “observation”(24.3%)(**Figure 5D**). In addition, “Cisplatin+Etoposide” was an effective chemotherapy regimen to improve the 2-year OS rate (OR=2.90, 95% CI 1.10 to 8.30) and 5-year OS rate (OR= 6.70, 95% CI 1.70 to 3.10) while “Cisplatin+Vinorelbine” had a significantly higher 5-year OS rate than observation arm (OR=1.70, 95% CI 1.10 to 2.90). However, there was no significant difference among the other chemotherapy regimens and observation arm with regard to 2-year OS rate, 3-year OS rate and 5-year OS rate (**Supplementary figure 3A-C**).

Secondary outcome---anemia, neutropenia, thrombocytopenia, febrile neutropenia and nausea/vomiting

15 clinical trials were enrolled to compare the presence of anemia among different adjuvant chemotherapy regimens. We found that the incidence of anemia in the

For peer review only

"Carboplatin+Pemetrexed" arm was significantly higher than the "Cisplatin+Vinorelbine" arm (OR= 5.7e+21, 95% CI 1.70 to 3.7e+71). However, there was no significant difference among the other chemotherapy regimens and "Cisplatin+Vinorelbine" (**Figure 6A**).

As for neutropenia, we enrolled 13 clinical trials to compare the safety of different adjuvant chemotherapy regimens. Patients receiving "Carboplatin+Gemcitabine" (OR=3.80e+33,95%CI 4.20 to 5.2e+91), "Carboplatin+Paclitaxel" (OR=7.30e+32, 95%CI 1.30 to 6.70e+90), " Cisplatin+Docetaxel" (OR=1.80e+33, 95%CI 2.70 to 2.10e+91) and "Cisplatin+Gemcitabine" (OR=2.6e+33, 95%CI 3.20 to 3.2e+91) arm were significantly observed the neutropenia compared with"Cisplatin+Vinorelbine". However, the incidence of neutropenia in the "Cisplatin+Vinorelbine" group was not significantly higher than the other chemotherapy regimens group (**Figure 6B**).

Also, as for thrombocytopenia, we enrolled 11 clinical trials to investigate the safety of different adjuvant chemotherapy regimens. We found that patients medicated with "Carboplatin+Pemetrexed" were significantly observed thrombocytopenia compared with "Cisplatin+Vinorelbine" (OR=2.20e+14, 95%CI 12 to 3.10e+44). However, there was no significant difference among other chemotherapy regimens and "Cisplatin+Vinorelbine"(Figure 6C).

In terms of febrile neutropenia, 6 clinical trials were included to compare the effect of different chemotherapy regimens. Our results showed that there was no significant difference among these chemotherapy regimens and "Cisplatin+Vinorelbine" (**Figure 6D**).

For peer review only

In addition, 14 clinical trials were enrolled to analyze the observation of nausea/vomiting in NSCLC patients with postoperative adjuvant chemotherapy. We found that compared to “Cisplatin+Vinorelbine”,“Carboplatin+Gemcitabine” (OR=3.8e+33, 95%CI 4.20 to 5.20e+91), “Carboplatin+Paclitaxel” (OR=7.30e+32, 95%CI 1.30 to 6.70e+90), “Cisplatin+Docetaxel” (OR=1.8e+33, 95%CI 2.70 to 2.10e+91), “Cisplatin+Gemcitabine” (OR=2.60e+33, 95%CI 3.20 to 3.20e+91) had higher incidence of nausea/vomiting. However, there was no significant difference among the other chemotherapy regimens and “Cisplatin+Vinorelbine”(**Figure 6E**).

For peer review only

Discussion

Recent advances in precision medicine have shaped the future of adjuvant therapy for patients with completely resected NSCLC [19]. However, for patients not applicable to targeted therapy or immunotherapy, cytotoxicity chemotherapy is still recommended by guidelines as the standard of care. It is of great significance to investigate the best platinum regimen in the postoperative adjuvant chemotherapy setting for early-stage NSCLC. Our results showed that adjuvant postoperative chemotherapy was beneficial to improve the RFS and OS compared with the observation arm. Among these chemotherapy regimens, the "Cisplatin+Vinorelbine" arm was an effective therapeutic method to improve survival with tolerable toxicity while the "Cisplatin+Pemetrexed" arm did not take advantage of the other therapeutic methods in improving survival. Also, the "Cisplatin+Etoposide" arm contributed to improving the RFS and OS rate for patients with resected NSCLC.

A meta-analysis, published in 1995, demonstrated that postoperative chemotherapy had a significant advantage in 5-year survival compared with the observation group, which opened up a new era of postoperative chemotherapy [37]. Moreover, a pooled analysis also indicated the promising role of cisplatin-based adjuvant chemotherapy on the 5-year survival [6]. Based on these findings, it is universally acknowledged that adjuvant cytotoxicity chemotherapy integration of platinum and third-generation agents is conducive to the improvement of postsurgical survival for NSCLC patients. In the present study, the survival benefit was also observed in the platinum adjuvant chemotherapy setting. The adjuvant chemotherapy regimens were not limited to

For peer review only

cisplatin-based chemotherapy, which extended to cisplatin/carboplatin in combination with other third-generation agents. Furthermore, the peak recurrence of patients with early-stage NSCLC is 2 years later after surgery and a higher 5-year RFS rate represented the lower possibility of postoperative relapse. In our study, not only 5-year RFS and OS benefit but the improvement of RFS, OS, 2-year RFS and OS rate, and 3-year RFS and OS rate were observed in the adjuvant chemotherapy group. These significant survival advantages could be explained in part by the fact that postoperative adjuvant chemotherapy could decrease the presence of micro-metastases of cancer cells.

According to the subgroup analysis conducted by LACE in 2010,“Cisplatin+Vinorelbine” was an effective therapeutic method for the improvement of survival with a manageable toxicity profile [8]. Over the few decades, the introduction of the other third-generation cytotoxicity chemotherapy regimens such as Pemetrexed and Docetaxel brought into the development of platinum adjuvant chemotherapy [10-12]. Noteworthy, regardless of the absence of level 1 data in the adjuvant setting, due to its gold status in non-squamous advanced NSCLC with superior efficacy and low tolerability, “Cisplatin+ Pemetrexed” is still reasonably recommended as a standard of care for patients with early-stage resectable non-squamous NSCLC [13, 14]. However, the other chemotherapy regimens did not show their significant survival advantages over the observation arm in the adjuvant setting in this analysis. In addition, a subgroup analysis conducted by a large clinical trial---ECOG1505, did not find a significant difference among four platinum-based

For peer review only

chemotherapy regimens (“Cisplatin+Vinorelbine”, “Cisplatin+Docetaxel”, “Cisplatin+Gemcitabine” and “Cisplatin+Pemetrexed”)[38]. On the one hand, the absolute effect of adjuvant cytotoxicity chemotherapy on stage IB and squamous patients is still under debated [10, 39]. Hence, it seemed that the “Cisplatin+Vinorelbine” arm still played a vital role in the adjuvant setting for patients with NSCLC since its efficacy was not influenced by the histology [8]. By contrast, the negative results of “Cisplatin+ Pemetrexed” implies the restrictions of its application in the adjuvant chemotherapy setting for patients with squamous. Moreover, some biomarkers such as the excision repair cross-complementation group 1 (ERCC1), b-Tubulin, and Mucin hold the potential to predict the efficacy of cytotoxicity chemotherapy in the adjuvant setting [40-42]. Therefore, the heterogeneity of biological characteristics of each patient imposed confounding factors to the comparisons of different chemotherapy regimens.

According to our analysis, “Cisplatin + Etoposide”, the gold chemotherapy regimens for small cell lung cancer, did have a significantly higher survival rate over observation arm in the patients with early-stage resectable NSCLC. Mario Roselli et.al conducted a 10-year follow-up clinical trials to demonstrate the effect of “Cisplatin+Etoposide” on the reduction of recurrences and the extension of long-term survival, which enrolled only patients with stage I NSCLC and adopted the relatively high dose of Cisplatin (100mg/m2) and Etoposide (120mg/m2) [31]. Hence, considering the potential bias, the positive results of “Cisplatin+Etoposide” need to

For peer review only

be interpreted with caution. Our analysis may bring some guiding significance to the clinicians when they make clinical decisions of the optimal chemotherapy regimen. Anti-angiogenic therapy such as bevacizumab and endostar, hold the potential to improve the efficacy of adjuvant chemotherapy probably through inhibiting the growth of micro-metastatic cancer cells and increasing the sensitivity of tumor cells to chemotherapy [25, 43, 44]. However, our results showed that the combination of chemotherapy regimens and endostar did not take advantage over a single platinum-based chemotherapy arm. Furthermore, according to the results of ECOG1505, there was no significant difference between the adjuvant chemotherapy with or without bevacizumab for patients with resected NSCLC [38]. One of the reasons to explain these negative results might be the heterogeneity in the expression of vascular endothelial growth factor (VEGF). Patients with high VEGF expression tend to obtain more benefits from anti-angiogenic therapy than the low VEGF expression ones [25].

In terms of the safety of adjuvant chemotherapy, the incidence of hematological toxicities and nausea/vomiting, which was greatly influenced by the dose and schedules, were not significantly higher in the “Cisplatin+Vinorelbine” arm than other chemotherapy. Noteworthy, the schedules in the adjuvant setting was quite different from the one utilized in the advanced NSCLC patients [45]. Therefore, “Cisplatin+Vinorelbine” should be recommended as an adjuvant cytotoxicity chemotherapy regimen with an acceptable safety profile. On the contrary, the incidence of anemia and thrombocytopenia in the “Carboplatin+Pemetrexed” group

For peer review only

was significantly higher than the "Cisplatin+Vinorelbine" group. Both the "Carboplatin" and "Pemetrexed" are characterized as relatively safe cytotoxicity chemotherapy regimens and recommended to be utilized for patients with medical commodities. The contradictory outcomes could be explained in part by the relatively lower dose of "Cisplatin+Vinorelbine".

To the best of our knowledge, the current systematic review and Bayesian network meta-analysis was the first study to compare the efficacy and safety of different adjuvant chemotherapy regimens directly for NSCLC patients. Despite the strengths, it was evident that there still existed some limitations in the current study. Firstly, for nearly half of the enrolled studies, the risk of bias in certain items remained unclear and the quality of them could not be evaluated. Secondary, due to the lack of specific data, the subgroup analysis stratified by the stage and pathology could not be performed. Thus, it is difficult to determine the optimal subgroup of patients who may obtain the benefit from certain cytotoxicity chemotherapy regimens. Furthermore, we did not incorporate any targeted or immune-biological therapy into the analysis due to the open-loop of interventions. Last but not at least, although the absolute effect of radiotherapy for patients with completely resected NSCLC was presented recently, we did not conduct a subgroup analysis stratified by postoperative radiotherapy or not as it was undertaken according to every center's policy.

Conclusion

In conclusion, the current study summarized the adjuvant cytotoxicity chemotherapy regimens for patients with early-stage resected NSCLC. Researches on adjuvant

For peer review only

cytotoxicity chemotherapy might be an out-of-date topic but numerous NSCLC patients could obtain benefit from the optimal cytotoxicity chemotherapy regimen. "Cisplatin+Vinorelbine" had a significant survival advantage with a relatively good safety profile in the adjuvant setting while the "Cisplatin+Pemetrexed" arm was not superior to the other therapeutic methods in improving survival.

For peer review only

Contributorship statement

Wenfeng Fang: Conceptualization,Lanlan Pang& Jiadi Gan & Yihua Huang: Data curation,Lanlan Pang & Jiadi Gan: Formal analysis,Wenfeng Fang:Funding acquisition,Lanlan Pang: Investigation,Lanlan Pang:Methodology,Wenfeng Fang: Project administration,Lanlan Pang: Resources,Lanlan Pang: Software,Li Zhang &Wenfeng Fang: Supervision,

Jiadi Gan:Validation Lanlan Pang: Visualization,All authors: Roles/Writing - original draft,All Authors: Writing - review & editing.

Competing Interest

The authors declare no potential conflicts of interest.

Funding

This work was supported by Chinese National Natural Science Foundation Project grant number 81772476.

Data sharing

No additional data available.

For peer review only

References

1. Sung, H., et al., *Global Cancer Statistics 2020: GLOBOCAN Estimates of Incidence and Mortality Worldwide for 36 Cancers in 185 Countries.* CA Cancer J Clin, 2021. **71**(3): p. 209-249.doi:10.3322/caac.21660

2. Zappa, C. and S.A. Mousa, *Non-small cell lung cancer: current treatment and future advances.* Transl Lung Cancer Res, 2016. **5**(3): p. 288-300.doi:10.21037/tlcr.2016.06.07

3. Mountain, C.F., *Revisions in the International System for Staging Lung Cancer.* Chest, 1997. **111**(6): p. 1710-7.doi:10.1378/chest.111.6.1710

4. Goldstraw, P., et al., *The IASLC Lung Cancer Staging Project: Proposals for Revision of the TNM Stage Groupings in the Forthcoming (Eighth) Edition of the TNM Classification for Lung Cancer.* J Thorac Oncol, 2016. **11**(1): p. 39-51.doi:10.1016/j.jtho.2015.09.009

5. Rosell, R., et al., *The role of chemotherapy in early non-small-cell lung cancer management.* Lung Cancer, 2001. **34 Suppl 3**: p. S63-74.doi:10.1016/s0169-5002(01)00376-2

6. Pignon, J.P., et al., *Lung adjuvant cisplatin evaluation: a pooled analysis by the LACE Collaborative Group.* J Clin Oncol, 2008. **26**(21): p. 3552-9.doi:10.1200/jco.2007.13.9030

7. *Cancer Care Ontario and American Society of Clinical Oncology Adjuvant Chemotherapy and Adjuvant Radiation Therapy for Stages I-IIIA Resectable Non-Small-Cell Lung Cancer Guideline.* J Oncol Pract, 2007. **3**(6): p.

For peer review only

332-335.doi:10.1200/jop.0768503

8. Douillard, J.Y., et al., *Adjuvant cisplatin and vinorelbine for completely resected non-small cell lung cancer: subgroup analysis of the Lung Adjuvant Cisplatin Evaluation.* J Thorac Oncol, 2010. **5**(2): p. 220-8.doi:10.1097/JTO.0b013e3181c814e7

9. Postmus, P.E., et al., *Early and locally advanced non-small-cell lung cancer (NSCLC): ESMO Clinical Practice Guidelines for diagnosis, treatment and follow-up.* Ann Oncol, 2017. **28**(suppl_4): p. iv1-iv21.doi:10.1093/annonc/mdx222

10. Strauss, G.M., et al., *Adjuvant paclitaxel plus carboplatin compared with observation in stage IB non-small-cell lung cancer: CALGB 9633 with the Cancer and Leukemia Group B, Radiation Therapy Oncology Group, and North Central Cancer Treatment Group Study Groups.* J Clin Oncol, 2008. **26**(31): p. 5043-51.doi:10.1200/jco.2008.16.4855

11. Chen, Q., et al., *Clinical observation of docetaxel or gemcitabine combined with cisplatin in the chemotherapy after surgery for stage II-III non-small cell lung cancer.* Pak J Med Sci, 2015. **31**(5): p. 1095-8.doi:10.12669/pjms.315.7380

12. Kreuter, M., et al., *Randomized phase 2 trial on refinement of early-stage NSCLC adjuvant chemotherapy with cisplatin and pemetrexed versus cisplatin and vinorelbine: the TREAT study.* Ann Oncol, 2013. **24**(4): p. 986-92.doi:10.1093/annonc/mds578

For peer review only

13. Scagliotti, G.V., et al., *Phase III study comparing cisplatin plus gemcitabine with cisplatin plus pemetrexed in chemotherapy-naive patients with advanced-stage non-small-cell lung cancer.* J Clin Oncol, 2008. **26**(21): p. 3543-51.doi:10.1200/jco.2007.15.0375

14. National Comprehensive Cancer Network. NCCN Clinical Practice Guidelines in Oncology. Non-Small Cell Lung Cancer. Version 1.2021.Available at https://www.nccn.org/professionals/physician_gls/pdf/nscl.pdf. Accessed on December 10, 2020

15. Ardizzoni, A., et al., *Cisplatin- versus carboplatin-based chemotherapy in first-line treatment of advanced non-small-cell lung cancer: an individual patient data meta-analysis.* J Natl Cancer Inst, 2007. **99**(11): p. 847-57.doi:10.1093/jnci/djk196

16. Gridelli, C., et al., *Chemotherapy of advanced NSCLC in special patient population.* Ann Oncol, 2006. **17 Suppl 5**: p. v72-8.doi:10.1093/annonc/mdj955

17. Goss, G.D., et al., *Gefitinib versus placebo in completely resected non-small-cell lung cancer: results of the NCIC CTG BR19 study.* J Clin Oncol, 2013. **31**(27): p. 3320-6.doi:10.1200/jco.2013.51.1816

18. Kelly, K., et al., *Adjuvant Erlotinib Versus Placebo in Patients With Stage IB-IIIA Non-Small-Cell Lung Cancer (RADIANT): A Randomized, Double-Blind, Phase III Trial.* J Clin Oncol, 2015. **33**(34): p. 4007-14.doi:10.1200/jco.2015.61.8918

For peer review only

19. Zhong, W.Z., et al., *Gefitinib versus vinorelbine plus cisplatin as adjuvant treatment for stage II-IIIA (N1-N2) EGFR-mutant NSCLC (ADJUVANT/CTONG1104): a randomised, open-label, phase 3 study.* Lancet Oncol, 2018. **19**(1): p. 139-148.doi:10.1016/s1470-2045(17)30729-5

20. Fukumoto, S.I., et al., *A randomized phase II trial of cisplatin plus gemcitabine versus carboplatin plus gemcitabine in patients with completely resected non-small cell lung cancer: Hokkaido Lung Cancer Clinical Study Group Trial (HOT0703).* Cancer Chemother Pharmacol, 2020. **86**(1): p. 117-127.doi:10.1007/s00280-020-04104-1

21. Tada, H., et al., *A randomized trial comparing adjuvant chemotherapy versus surgery alone for completely resected pN2 non-small cell lung cancer (JCOG9304).* Lung Cancer, 2004. **43**(2): p. 167-73.doi:10.1016/j.lungcan.2003.08.028

22. Kenmotsu, H., et al., *Randomized Phase III Study of Pemetrexed Plus Cisplatin Versus Vinorelbine Plus Cisplatin for Completely Resected Stage II to IIIA Nonsquamous Non-Small-Cell Lung Cancer.* J Clin Oncol, 2020. **38**(19): p. 2187-2196.doi:10.1200/jco.19.02674

23. Ohta, M., et al., *Adjuvant chemotherapy for completely resected stage III non-small-cell lung cancer. Results of a randomized prospective study. The Japan Clinical Oncology Group.* J Thorac Cardiovasc Surg, 1993. **106**(4): p. 703-8

24. Kubota, K., et al., *Randomized phase II trial of adjuvant chemotherapy with*

For peer review only

docetaxel plus cisplatin versus paclitaxel plus carboplatin in patients with completely resected non-small cell lung cancer: TORG 0503. Lung Cancer, 2020. **141**: p. 32-36.doi:10.1016/j.lungcan.2019.11.009

25. Chen, Z., et al., *Endostar in combination with postoperative adjuvant chemotherapy prolongs the disease free survival of stage IIIA NSCLC patients with high VEGF expression.* Oncotarget, 2017. **8**(45): p. 79703-79711.doi:10.18632/oncotarget.19114

26. Zhong, W.Z., et al., *Randomized Study of Adjuvant Docetaxel vs. Observation for Completely Resected Stage IB-IIIa NSCLC with 11 Years' Median Follow-Up.* Journal of Thoracic Oncology, 2017. **12**(1): p. S642-S643

27. YANG Yan-zhuo,et.al.,*Recombinant human endostatin combined with TP regimen as postoperative adjuvant treatment for non-small-cell lung cancer*.Med J Chin PLA, Vol. 37, No. 1, January 1, 2012.

28. Barlesi, F., et al., *A randomized trial comparing adjuvant chemotherapy with gemcitabine plus cisplatin with docetaxel plus cisplatin in patients with completely resected non-small-cell lung cancer with quality of life as the primary objective.* Interact Cardiovasc Thorac Surg, 2015. **20**(6): p. 783-90.doi:10.1093/icvts/ivv050

29. Dunant, A., J.P. Pignon, and T. Le Chevalier, *Adjuvant chemotherapy for non-small cell lung cancer: contribution of the International Adjuvant Lung Trial.* Clin Cancer Res, 2005. **11**(13 Pt 2): p. 5017s-5021s.doi:10.1158/1078-0432.Ccr-05-9006

For peer review only

30. Kreuter, M., et al., *Three-Year Follow-Up of a Randomized Phase II Trial on Refinement of Early-Stage NSCLC Adjuvant Chemotherapy with Cisplatin and Pemetrexed versus Cisplatin and Vinorelbine (the TREAT Study).* J Thorac Oncol, 2016. **11**(1): p. 85-93.doi:10.1016/j.jtho.2015.09.014

31. Roselli, M., et al., *Postsurgical chemotherapy in stage IB nonsmall cell lung cancer: Long-term survival in a randomized study.* Int J Cancer, 2006. **119**(4): p. 955-60.doi:10.1002/ijc.21933

32. Winton, T., et al., *Vinorelbine plus cisplatin vs. observation in resected non-small-cell lung cancer.* N Engl J Med, 2005. **352**(25): p. 2589-97.doi:10.1056/NEJMoa043623

33. Waller, D., et al., *Chemotherapy for patients with non-small cell lung cancer: the surgical setting of the Big Lung Trial.* Eur J Cardiothorac Surg, 2004. **26**(1): p. 173-82.doi:10.1016/j.ejcts.2004.03.041

34. Douillard, J.Y., et al., *Adjuvant vinorelbine plus cisplatin versus observation in patients with completely resected stage IB-IIIA non-small-cell lung cancer (Adjuvant Navelbine International Trialist Association [ANITA]): a randomised controlled trial.* Lancet Oncol, 2006. **7**(9): p. 719-27.doi:10.1016/s1470-2045(06)70804-x

35. Schmid-Bindert, G., et al., *A randomized Phase 2 study of pemetrexed in combination with cisplatin or carboplatin as adjuvant chemotherapy in patients with completely resected stage IB or II Non-Small-Cell Lung Cancer.* Lung Cancer, 2015. **90**(3): p. 397-404.doi:10.1016/j.lungcan.2015.11.007

For peer review only

36. Felip, E., et al., *Preoperative chemotherapy plus surgery versus surgery plus adjuvant chemotherapy versus surgery alone in early-stage non-small-cell lung cancer.* J Clin Oncol, 2010. **28**(19): p. 3138-45.doi:10.1200/jco.2009.27.6204

37. *Chemotherapy in non-small cell lung cancer: a meta-analysis using updated data on individual patients from 52 randomised clinical trials. Non-small Cell Lung Cancer Collaborative Group.* Bmj, 1995. **311**(7010): p. 899-909

38. Wakelee, H.A., et al., *Adjuvant chemotherapy with or without bevacizumab in patients with resected non-small-cell lung cancer (E1505): an open-label, multicentre, randomised, phase 3 trial.* Lancet Oncol, 2017. **18**(12): p. 1610-1623.doi:10.1016/s1470-2045(17)30691-5

39. Scagliotti, G., et al., *The differential efficacy of pemetrexed according to NSCLC histology: a review of two Phase III studies.* Oncologist, 2009. **14**(3): p. 253-63.doi:10.1634/theoncologist.2008-0232

40. Friboulet, L., et al., *ERCC1 isoform expression and DNA repair in non-small-cell lung cancer.* N Engl J Med, 2013. **368**(12): p. 1101-10.doi:10.1056/NEJMoa1214271

41. Graziano, S.L., et al., *Prognostic significance of mucin and p53 expression in stage IB non-small cell lung cancer: a laboratory companion study to CALGB 9633.* J Thorac Oncol, 2010. **5**(6): p. 810-7.doi:10.1097/jto.0b013e3181d89f95

42. Reiman, T., et al., *Cross-validation study of class III beta-tubulin as a predictive marker for benefit from adjuvant chemotherapy in resected*

For peer review only

non-small-cell lung cancer: analysis of four randomized trials. Ann Oncol, 2012. **23**(1): p. 86-93.doi:10.1093/annonc/mdr033

43. Folkman, J., *Antiangiogenesis in cancer therapy--endostatin and its mechanisms of action.* Exp Cell Res, 2006. **312**(5): p. 594-607.doi:10.1016/j.yexcr.2005.11.015

44. Limaverde-Sousa, G., C. Sternberg, and C.G. Ferreira, *Antiangiogenesis beyond VEGF inhibition: a journey from antiangiogenic single-target to broad-spectrum agents.* Cancer Treat Rev, 2014. **40**(4): p. 548-57.doi:10.1016/j.ctrv.2013.11.009

45. Caffo, O., et al., *An evaluation of the pharmacokinetics and clinical use of vinorelbine for NSCLC treatment.* Expert Opin Drug Metab Toxicol, 2013. **9**(8): p. 1037-51.doi:10.1517/17425255.2013.804065

For peer review only

Figure list

Figure 1. Flow diagram of enrolled studies selection.

Figure 2.

A) The efficacy of platinum-based postoperative adjuvant chemotherapy in improving the RFS compared with the observation group.

B) 2-year RFS rate of the chemotherapy arm in comparison with the observation arm.

C) 3-year RFS rate of the chemotherapy arm in comparison with the observation arm.

D) 5-year RFS rate of the chemotherapy arm in comparison with the observation arm

Figure 3.

A) Network evidence of the comparisons for the best adjuvant chemotherapy with regard to RFS.

B) Forest plots of the comparisons for the different cytotoxicity chemotherapy regimens with regard to RFS.

C) The league table of the comparisons for the different cytotoxicity chemotherapy regimens with regard to RFS.

D) SUCRA value of every cytotoxicity chemotherapy regimens with regard to RFS.

Figure 4.

A) The efficacy of platinum-based postoperative adjuvant chemotherapy in improving the OS compared with the observation group.

B) 2-year OS rate of the chemotherapy arm compared to the observation arm.

C) 3-year OS rate of the chemotherapy arm compared to the observation arm.

D) 5-year OS rate of the chemotherapy arm compared to the observation arm.

For peer review only - http://bmjopen.bmj.com/site/about/guidelines.xhtml

Figure 5.

A) Network evidence of the comparisons for the best adjuvant chemotherapy with regard to OS.

B) Forest plots of the comparisons for the different cytotoxicity chemotherapy regimens with regard to OS.

C) The league table of the comparisons for the different cytotoxicity chemotherapy regimens with regard to OS.

D) SUCRA value of every cytotoxicity chemotherapy regimens with regard to OS.

Figure 6.

A) Forest plots of the comparisons for the different cytotoxicity chemotherapy regimens with regard to the presence of anemia.

B) Forest plots of the comparisons for the different cytotoxicity chemotherapy regimens with regard to the presence of neutropenia.

C) Forest plots of the comparisons for the different cytotoxicity chemotherapy regimens with regard to the presence of thrombocytopenia .

D) Forest plots of the comparisons for the different cytotoxicity chemotherapy regimens with regard to the presence of febrile neutropenia.

E) Forest plots of the comparisons for the different cytotoxicity chemotherapy regimens with regard to the presence of nausea/vomiting.

Supplementary Figure list

Supplementary Figure 1.

A) Risk of bias graph.

For peer review only

691 **B)** Risk of bias summary

692 **Supplementary Figure 2.**

693 **A)** Forest plots of the comparisons for the different cytotoxicity chemotherapy regimens with regard to 2-year RFS rate.

695 **B)** Forest plots of the comparisons for the different cytotoxicity chemotherapy regimens with regard to 3-year RFS rate.

697 **C)** Forest plots of the comparisons for the different cytotoxicity chemotherapy regimens with regard to 5-year RFS rate.

699 **Supplementary Figure 3.**

700 A) Forest plots of the comparisons for the different cytotoxicity chemotherapy regimens with regard to 2-year OS rate.

702 B) Forest plots of the comparisons for the different cytotoxicity chemotherapy regimens with regard to 3-year OS rate.

704 C) Forest plots of the comparisons for the different cytotoxicity chemotherapy regimens with regard to 5-year OS rate.

For peer review only

Table 1 Basic characteristics of enrolled studies and participants.

Trail	Publication year	Country of region	Chemotherapy regimes	Sample size	Median follow-up (months)	Phase	Radiotherapy	Patients characteristics (Pathology;stage,PS)	Age	Sex
ANITA	2006	Multi-Center	**Cisplatin**100mg/m2+**Vinorelbine**30mg/m2 VS.**Observation**	840(407vs.433)	76	III	Not mandatory.	NSCLC; Stage IB－IIIA	59 (32－75) VS.59 (18－75)	85%vs.87%
Big-lung-trial	2003	UK	**Cisplatin**80mg/m2+**Vinorelbine**30mg/m2 VS.**Observation**	66(37VS.29)	58.8	---	Not mandatory	NSCLC;Stage I－III	/	/
CALGB 9633	2004	North Carolina	**Carboplatin**(AUC-6) + **Paclitaxel** 200 mg/m2 VS. **Observation**	344(173VS.171)	74	III	Not-mentioned	NSCLC; T2 with pathologically negative lymph nodes	61(34-78) VS.62(40-81) (range)	65%VS.63%
CSLC0201	2016	China	**Carboplatin**+**Docetaxel** VS.**Observation**	82(43vs.39)	132	---	Not-mentioned	NSCLC; Stage IB-IIIA	/	/
Enriqueta Felip et.al	2010	Multi-Center	**Carboplatin**(AUC-6.0)+**Paclitaxel**200mg/m2 VS.**Observation**	423(211VS.212)	51	III	Postoperative radiotherapy was allowed in patients with pathologic N2 disease.	NSCLC ; Stage IA with tumor size more than 2 cm, IB, II, or T3N1	64(33-81) VS.64(36-89) (range)	86%VS.88%
Fabrice Barlesi et.al	2015	France	**Cisplatin**75mg/m2+**Gemcitabine**1250mg/m2VS. **Cisplatin**75mg/m2+**Docetaxel**75mg/m2	136(67VS.69)	20.2	---	Not mandatory.	NSCLC; Stage IB-III	57 (44－74) VS.57 (36－71)	75%vs.74%
HOT0703	2020	Japan	**Cisplatin**40mg/m2+**Gemcitabine**1000mg/m2 VS.**Carboplatin**(AUC-5)+**Gemcitabine** 1000mg/m2	102(51vs.51)	69.6	II	Not-mentioned	NSCLC; Stage IB-IIIA	63(40-72) VS.64(36-74) (range)	67%VS.63%
IALT	2004	France	**Cisplatin**100mg/m2+**Vinorelbine**30mg/m2VS .**Observation**	500(248VS.262)	49.2	---	Not allowed.	NSCLC; Stage I, II, or III	/	/
JBR10	2005;2010	North American	**Cisplatin**+**Vinorelbine**VS. **Observation**	482(240vs.242)	111.6	III	Not-mentioned	NSCLC; Stage IB (T2N0) or II (T1-2N1)	61(35-82) VS.60.5(34-78)	64%VS.66%

For peer review only

For peer review only - http://bmjopen.bmj.com/site/about/guidelines.xhtml

JCOG9304	2003	Japan	**Cisplatin**80mg/m2+**Vindesine**3mg/m2 VS.**Observation**	159(59VS.60)	---	---	Not-mentioned	NSCLC;N2	62 (41—75) VS.62 (43—74)	68%VS.62%
Jing Wang et.al	2012	China	**Cisplation**80mg/m2+**Vinorelbine**30mg/m2 VS.**Observation**	451(225VS.226)	46	---	Not-mentioned	NSCLC;Stage I, II, and IIIA	55(38-83) VS.58(38-82)	71%VS.75%
JIPANG	2020	Japan	**Cisplatin**75mg/m2+**Pemetrexed**500mg/m2VS. **Cisplatin**80mg/m2+**Vinorelbine**25mg/m2	804(402VS.402)	45.2	III	Not allowed.	Non-squamous NSCLC; N2 Stage II or IIIA	65(58-69) VS. 64 (57-67)	60%VS.58%
Mario Roselli et.al	2006	Italy	**Cisplatin**100mg/m2+**Etoposide**120mg/m2VS. **Observation**	140(70VS.70)	40.31	---	Not-mentioned	NSCLC;Stage IB disease (pT2N0)	64.7±9.9 VS.62.9±9.2	91%VS.76%
Mitsuo Ohta et.al	1993	Japan	**Cisplatin**80mg/m2+**Vindesine** 3mg/m2VS.**Observation**	181(90VS.91)	31.2	---	Not-mentioned	NSCLC; Stage III	56.3 ± 9.1 VS.58.9 ± 8.4	77%S. 87%
Qiuqiang Chen et.al	2015	China	**Cisplatin**75mg/m2+**Docetaxel**75mg/m2VS. **Cisplatin**75mg/m2+**Gemcitabine**1250mg/m2	92(45vs.47)	22	---	Not-mentioned	NSCLC; Stage II to III	55(32-67) VS.56(31-67) (range)	84%VS.87%
Schmid-Binder Geraldt	2015	Germany, France, and Spain	**Cisplatin**75mg/m2+**Pemetrexed**500mg/m2VS. **Carboplatin**(AUC-5)+**Pemetrexed** 500 mg/m2	112(63VS.59)	---	II	Not-mentioned	NSCLC; Stage IB, IIA or IIB	61(44-75) VS.59(43-69)	78%VS.70%
TORG0503	2019	Japan	**Cisplatin**80mg/m2+**Docetaxel**60mg/m2VS. **Carboplatin**AUC-6+**Paclitaxel** 200 mg/m2	111(58vs.53)	---	II	Not-mentioned	NSCLC; Stage IB, II and IIIA	63(33-70) VS.59(34-70)	60%VS.66%
TREAT	2015	German	**Cisplatin**50mg/m2+**Vinorelbine**25mg/m2VS. **Cisplatin**75mg/m2+**Pemetrexed**500mg/m2	132(67vs.65)	36m	II	Not allowed.	NSCLC; Stages IB, IIA, IIB,	58 (40 – 73) VS.60 (38 – 74)	72%VS. 77%
Yanzhuo Yang	2012	China	**Carboplatin**(AUC=5-6)+**Docetaxel**75mg/m2+ **Endostar**15mgVS.**Carboplatin**+**Docetaxel**	76(38vs.38)	22	---	Not-mentioned	NSCLC;Stage IB-III	55.6(36-74) VS.60.2(45-77)	31%vs.27%
Zhiwei Chen et.al	2017	China	**Cisplatin**75mg/m2+**Vinorelbine**25mg/m2+ **Endostar**7.5mg/m2VS.**Cisplatin**+**Vinorelbine**	250(125vs.125)	60	III	Not-mentioned	NSCLC; Stage IB to IIIA	58(33-75) VS.55.5(37-71)	66%VS.67%

NSCLC: non-small cell lung cancer; PS: performance status; AUC: area under the curve

For peer review only

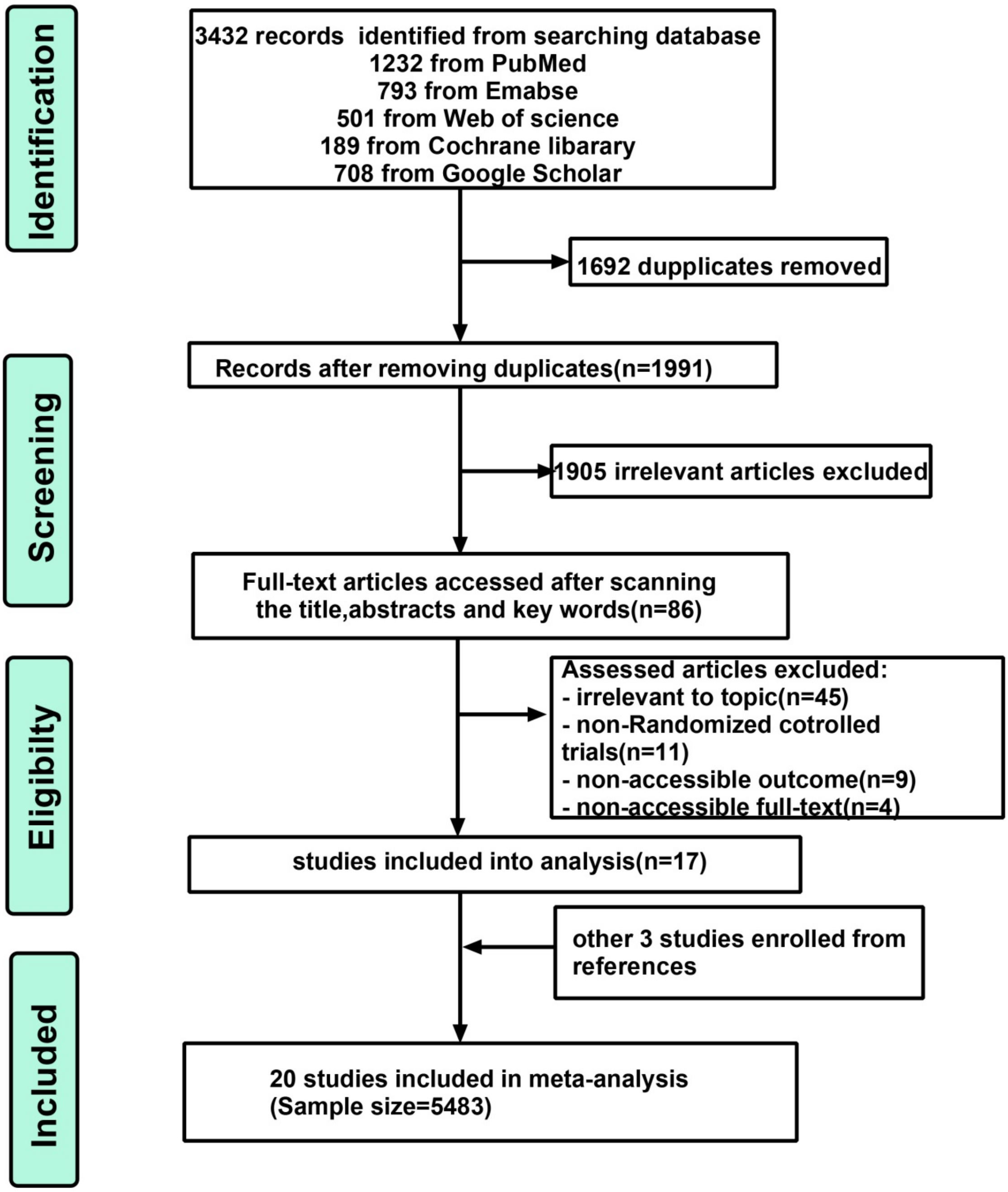

Figure 1. Flow diagram of enrolled studies selection.

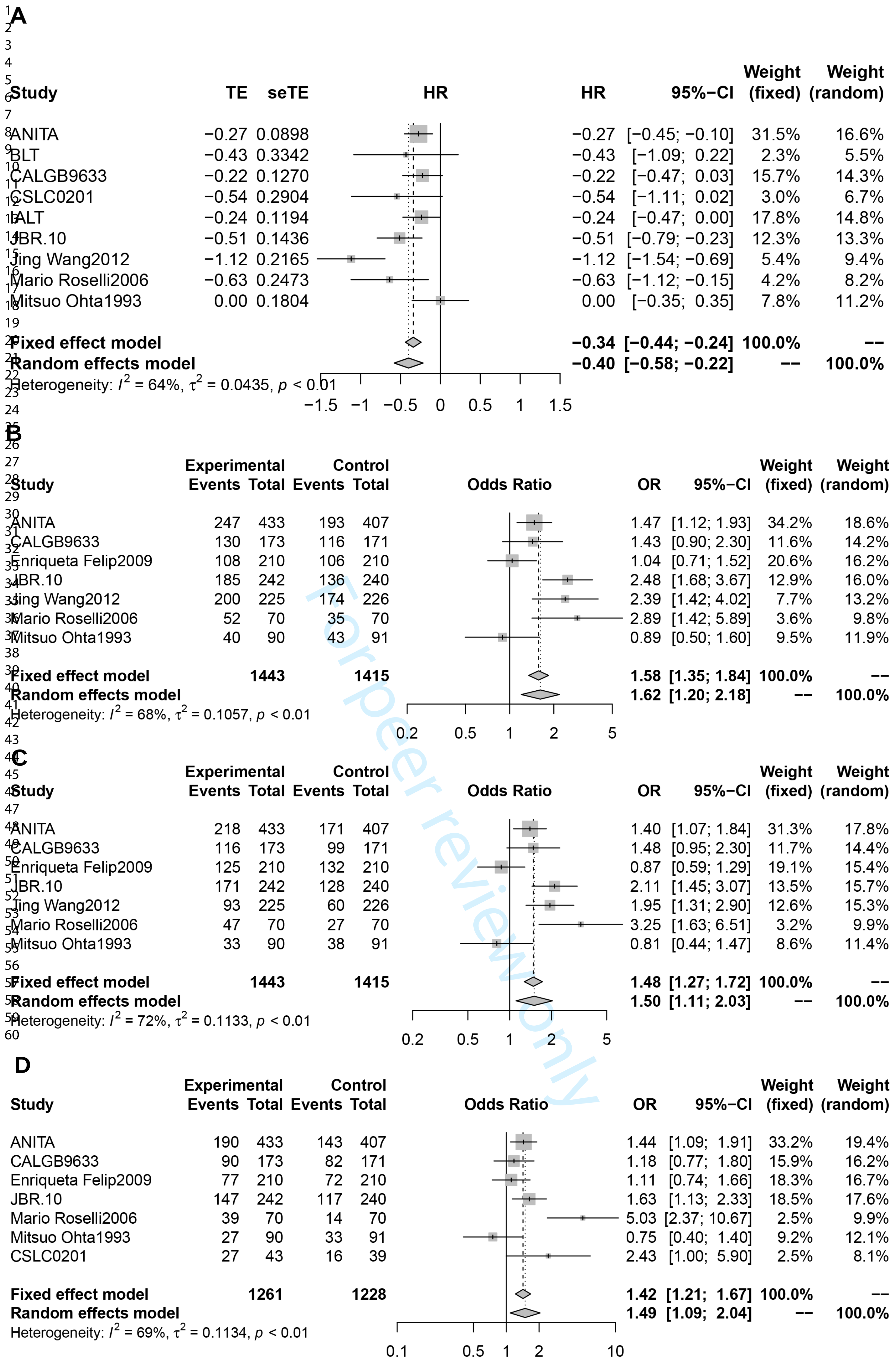

Figure 2.
A) The efficacy of platinum-based postoperative adjuvant chemotherapy in improving the RFS compared with the observation group.
B) 2-year;C) 3-year ; D) 5-year RFS rate of the chemotherapy arm in comparison with the observation arm.

For peer review only

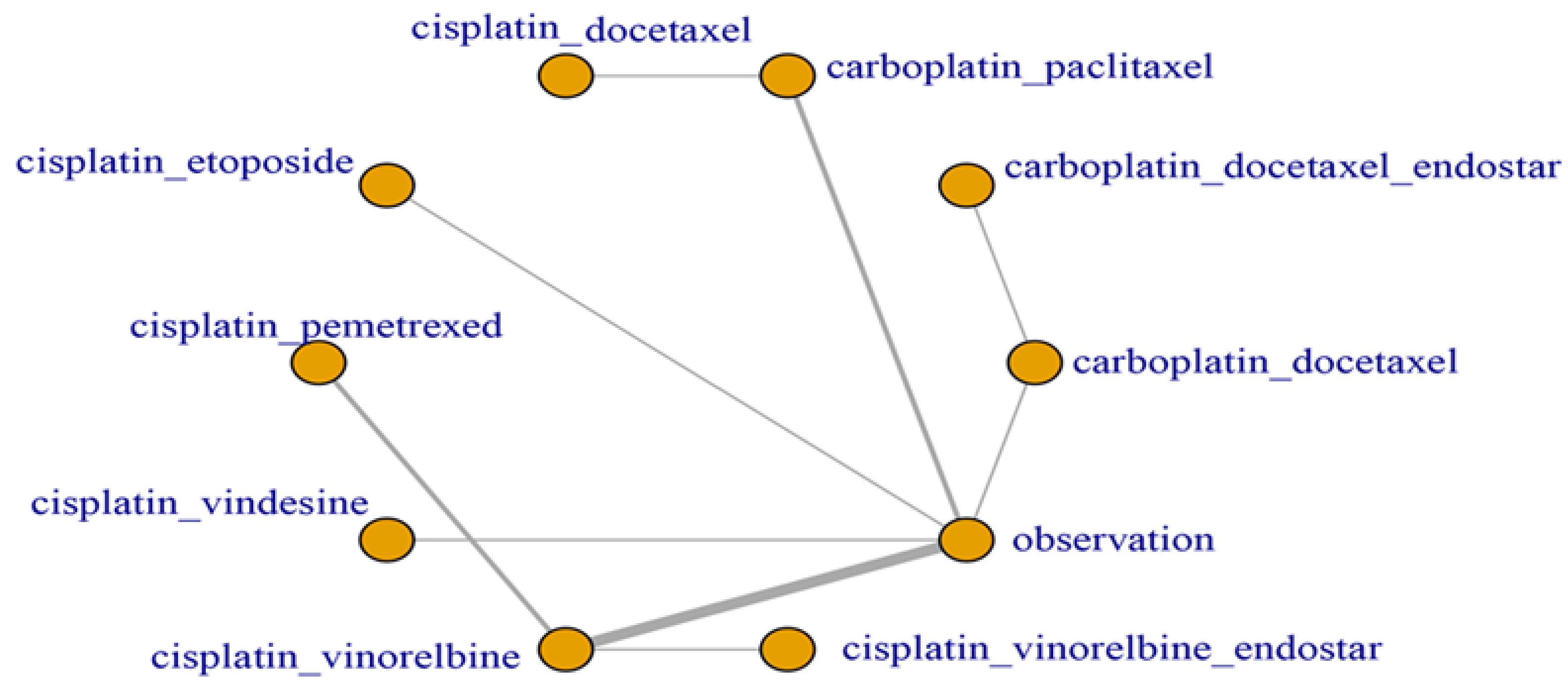

Figure 3.A) Network evidence of the comparisons for the best adjuvant chemotherapy with regard to RFS.

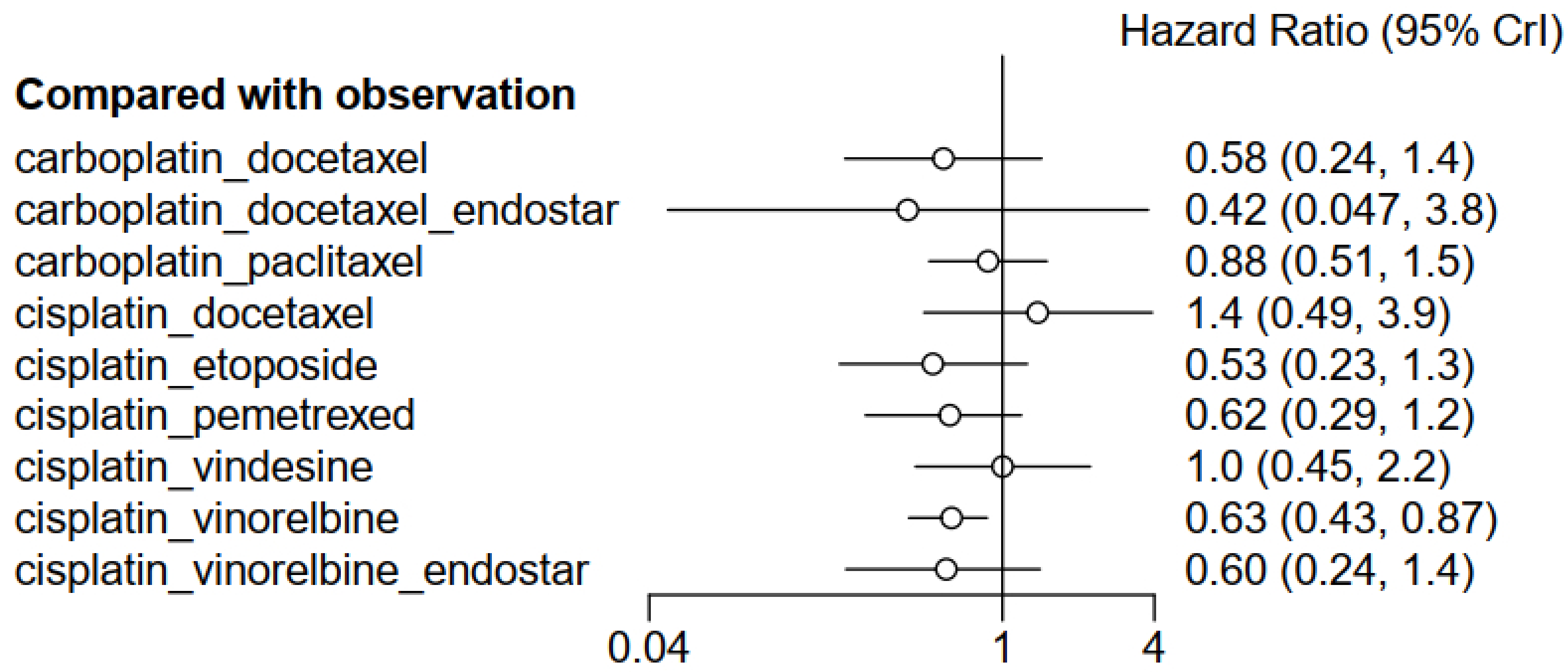

Figure4.B) Forest plots of the comparisons for the different cytotoxicity chemotherapy regimens with regard to RFS.

carboplatin_docetaxel									
1.37 (0.19, 10.16)	carboplatin_docetaxel_endostar								
0.66 (0.23, 1.9)	0.48 (0.05, 4.53)	carboplatin_paclitaxel							
0.42 (0.11, 1.68)	0.31 (0.03, 3.44)	0.63 (0.26, 1.57)	cisplatin_docetaxel						
1.1 (0.32, 3.84)	0.8 (0.08, 8.14)	1.66 (0.6, 4.56)	2.62 (0.68, 10.11)	cisplatin_etoposide					
0.94 (0.31, 3.14)	0.7 (0.07, 6.97)	1.42 (0.61, 3.71)	2.25 (0.67, 8.44)	0.86 (0.3, 2.77)	cisplatin_pemetrexed				
0.58 (0.17, 1.95)	0.43 (0.04, 4.28)	0.88 (0.33, 2.29)	1.39 (0.37, 5.13)	0.53 (0.16, 1.72)	0.62 (0.2, 1.7)	cisplatin_vindesine			
0.93 (0.36, 2.51)	0.68 (0.07, 6.23)	1.39 (0.75, 2.78)	2.21 (0.75, 6.84)	0.84 (0.34, 2.2)	0.98 (0.51, 1.78)	1.59 (0.68, 3.94)	cisplatin_vinorelbine		
0.97 (0.28, 3.54)	0.72 (0.07, 7.46)	1.46 (0.54, 4.26)	2.32 (0.61, 9.36)	0.88 (0.26, 3.15)	1.03 (0.36, 2.78)	1.67 (0.52, 5.69)	1.05 (0.47, 2.37)	cisplatin_vinorelbine_endostar	
0.58 (0.24, 1.43)	0.42 (0.05, 3.74)	0.88 (0.51, 1.5)	1.39 (0.49, 3.94)	0.53 (0.22, 1.25)	0.62 (0.28, 1.19)	1 (0.45, 2.22)	0.63 (0.43, 0.87)	0.6 (0.24, 1.41)	observation

Figure4.C) The league table of the comparisons for the different cytotoxicity chemotherapy regimens with regard to RFS.

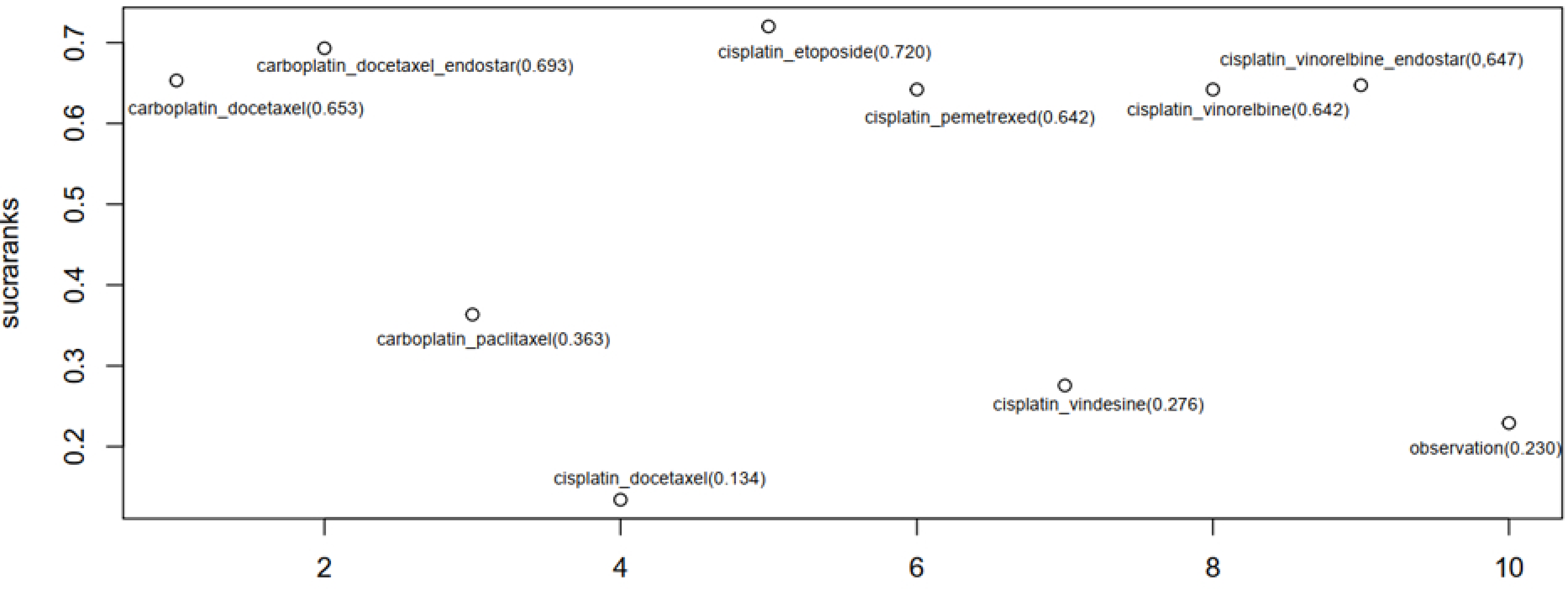

Figure4.D) SUCRA value of every cytotoxicity chemotherapy regimens with regard to RFS.

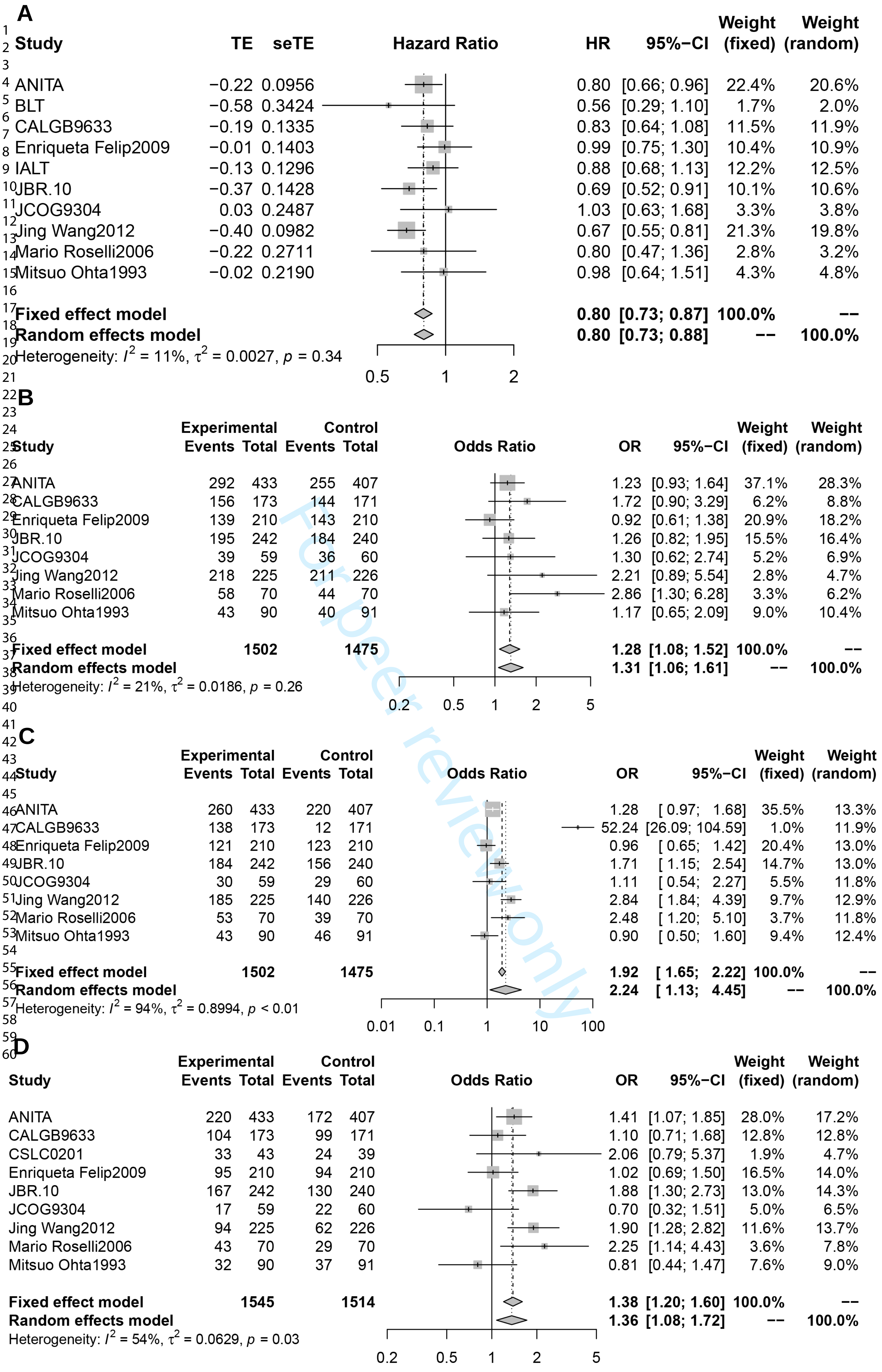

Figure 4.
A) The efficacy of platinum-based postoperative adjuvant chemotherapy in improving the OS compared with the observation group.
B) 2-year ;C) 3-year;D) 5-year OS rate of the chemotherapy arm compared to the observation arm.

For peer review only

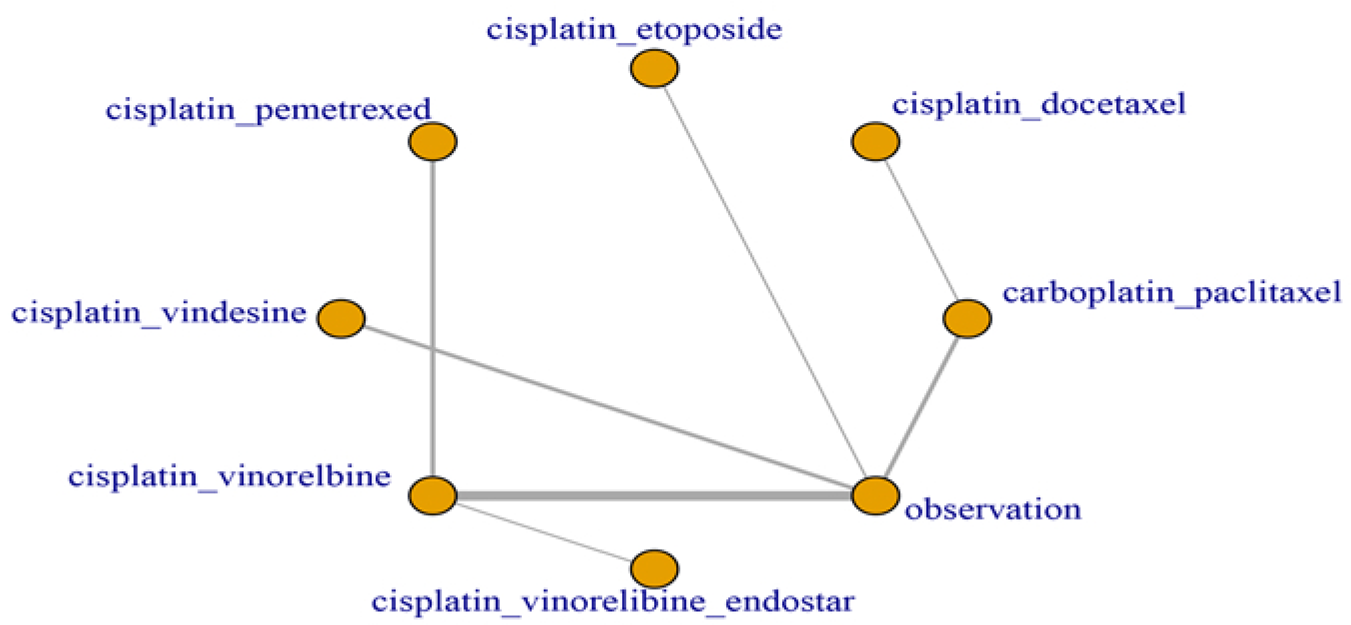

Figure 5.A) Network evidence of the comparisons for the best adjuvant chemotherapy with regard to OS.

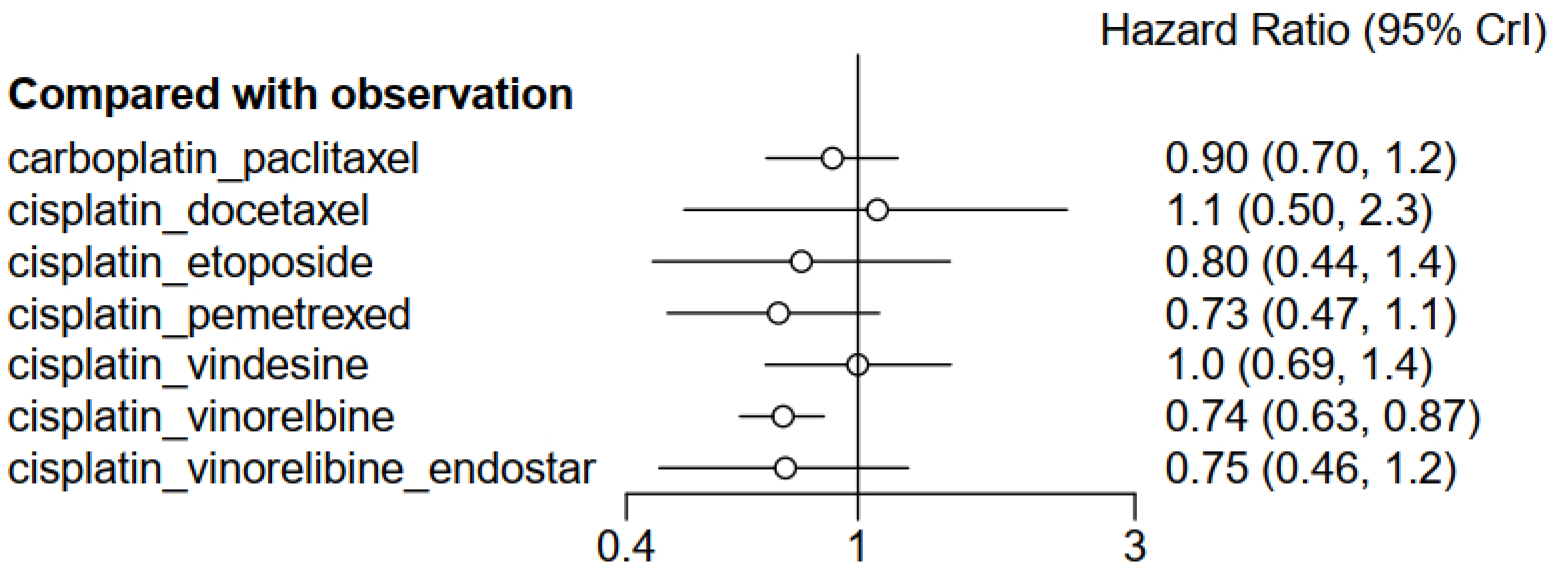

Figure5.B) Forest plots of the comparisons for the different cytotoxicity chemotherapy regimens with regard to OS.

carboplatin_docetaxel									
1.4 (0.19, 10.25)	carboplatin_docetaxel_endostar								
0.66 (0.23, 1.9)	0.48 (0.05, 4.67)	carboplatin_paclitaxel							
0.42 (0.11, 1.68)	0.3 (0.03, 3.47)	0.63 (0.26, 1.57)	cisplatin_docetaxel						
1.1 (0.32, 3.81)	0.78 (0.08, 8.43)	1.65 (0.6, 4.56)	2.61 (0.67, 10.04)	cisplatin_etoposide					
0.95 (0.31, 3.14)	0.68 (0.07, 7.09)	1.42 (0.61, 3.68)	2.26 (0.66, 8.36)	0.86 (0.3, 2.76)	cisplatin_pemetrexed				
0.58 (0.17, 1.95)	0.42 (0.04, 4.4)	0.88 (0.33, 2.29)	1.38 (0.37, 5.13)	0.53 (0.16, 1.71)	0.62 (0.2, 1.71)	cisplatin_vindesine			
0.93 (0.36, 2.51)	0.67 (0.07, 6.36)	1.39 (0.75, 2.76)	2.21 (0.75, 6.85)	0.85 (0.34, 2.21)	0.98 (0.51, 1.78)	1.59 (0.68, 3.93)	cisplatin_vinorelbine		
0.97 (0.28, 3.56)	0.7 (0.07, 7.71)	1.46 (0.54, 4.28)	2.32 (0.6, 9.34)	0.89 (0.27, 3.13)	1.03 (0.36, 2.79)	1.67 (0.52, 5.67)	1.05 (0.47, 2.36)	cisplatin_vinorelbine_endostar	
0.58 (0.23, 1.44)	0.42 (0.05, 3.84)	0.88 (0.51, 1.5)	1.39 (0.48, 3.94)	0.53 (0.23, 1.25)	0.62 (0.28, 1.19)	1 (0.45, 2.22)	0.63 (0.43, 0.87)	0.6 (0.24, 1.4)	observation

Figure5.C) The league table of the comparisons for the different cytotoxicity chemotherapy regimens with regard to OS.

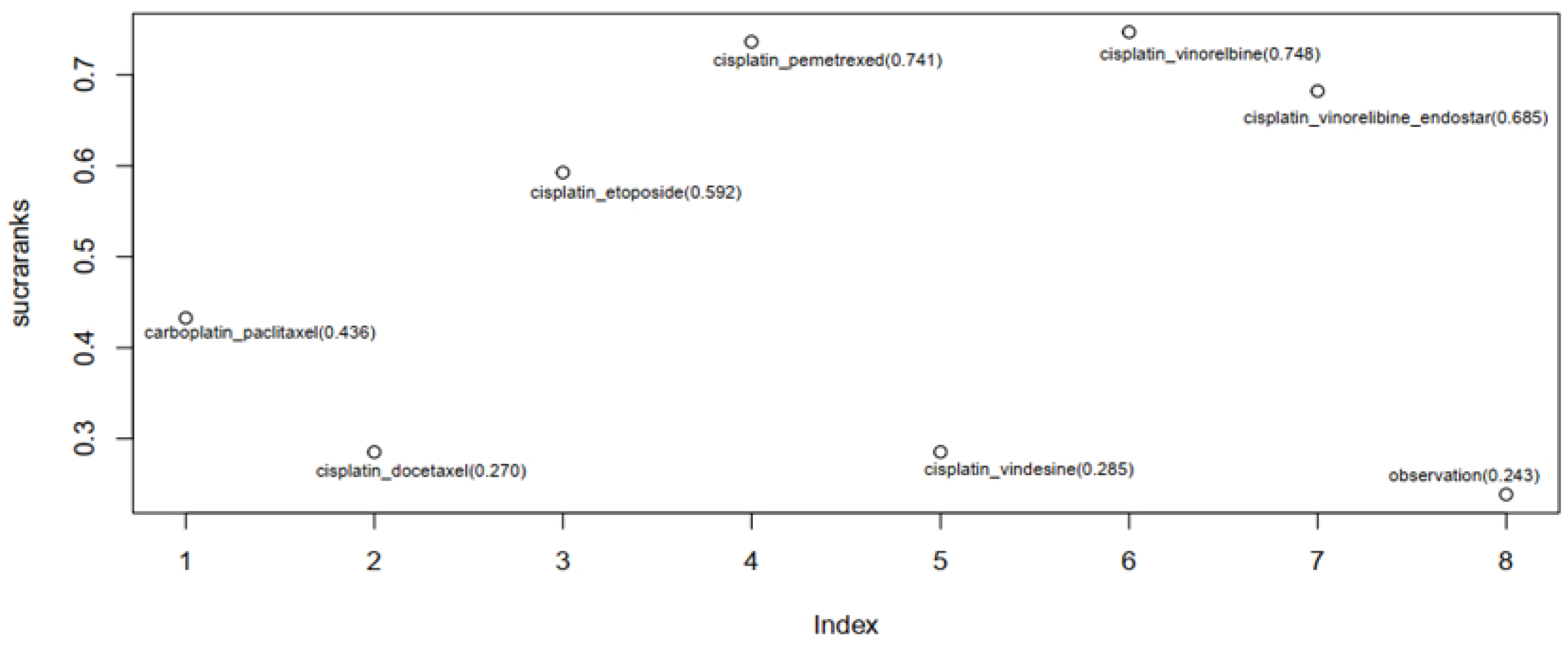

Figure5.D) SUCRA value of every cytotoxicity chemotherapy regimens with regard to OS.

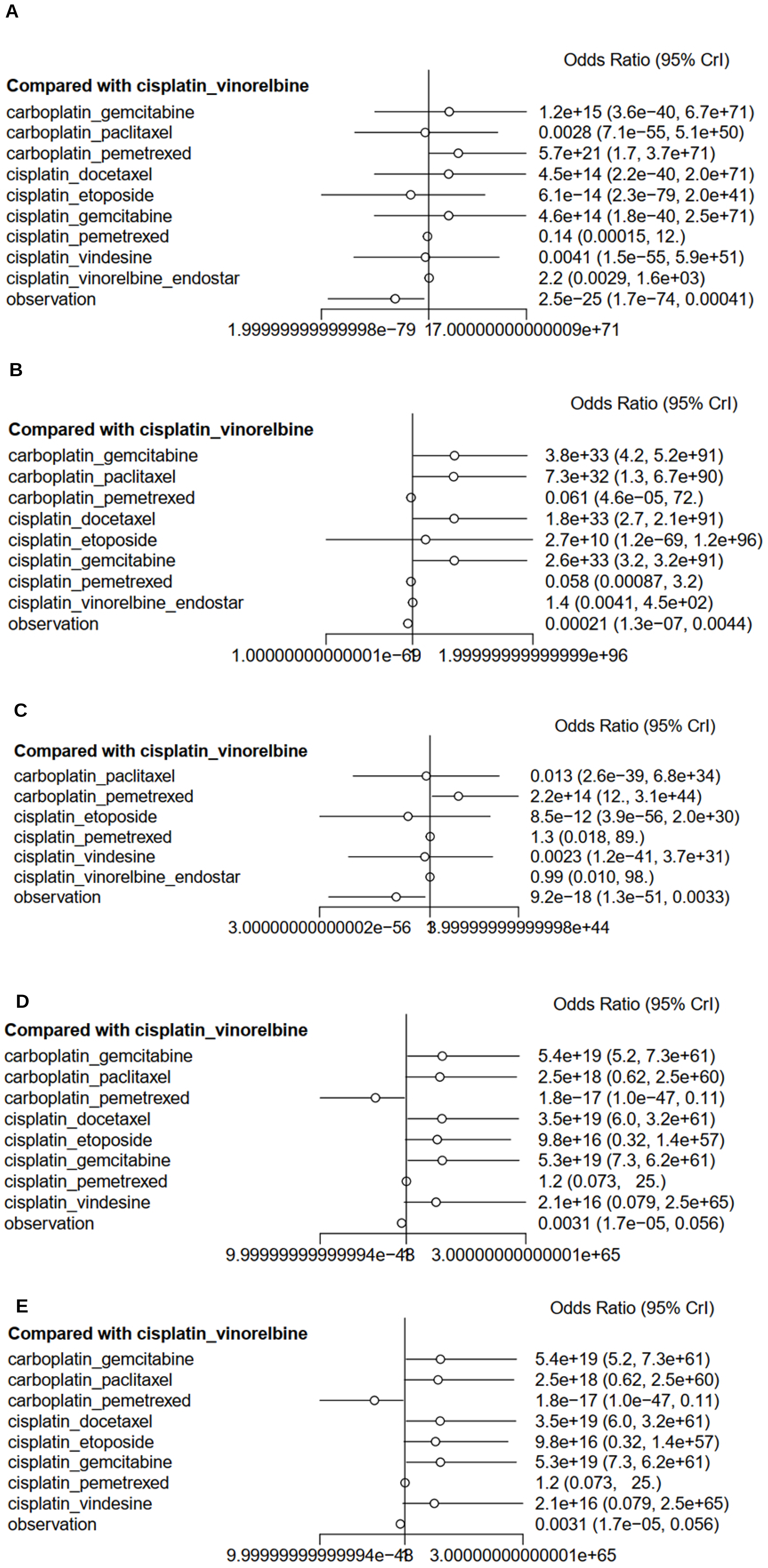

Figure 6.
Forest plots of the comparisons for the different cytotoxicity chemotherapy regimens with regard to the presence of (A)anemia;(B)neutropenia;(C);thrombocytopenia (D)febrile neutropenia;(E)nausea/vomiting.

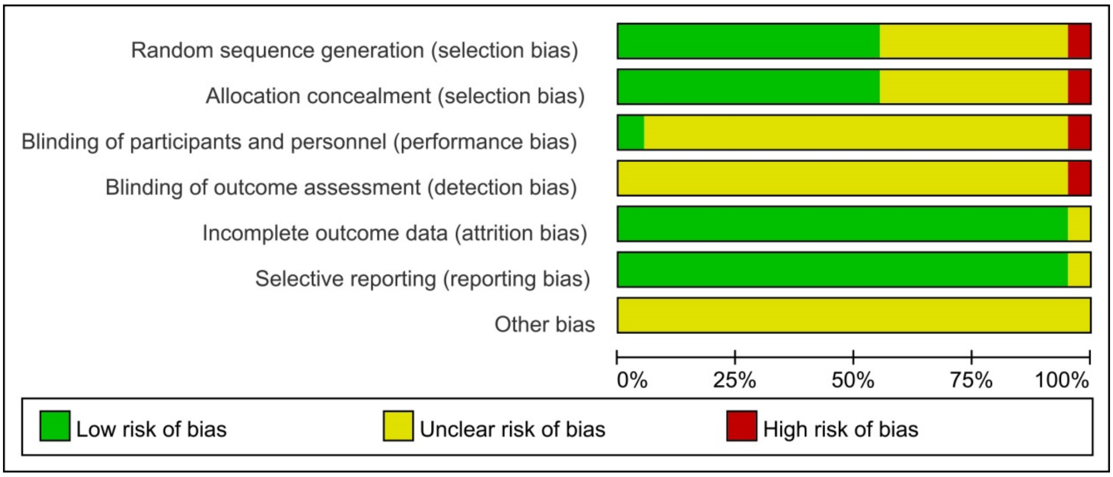

Supplementary Figure 1. A) Risk of bias graph.

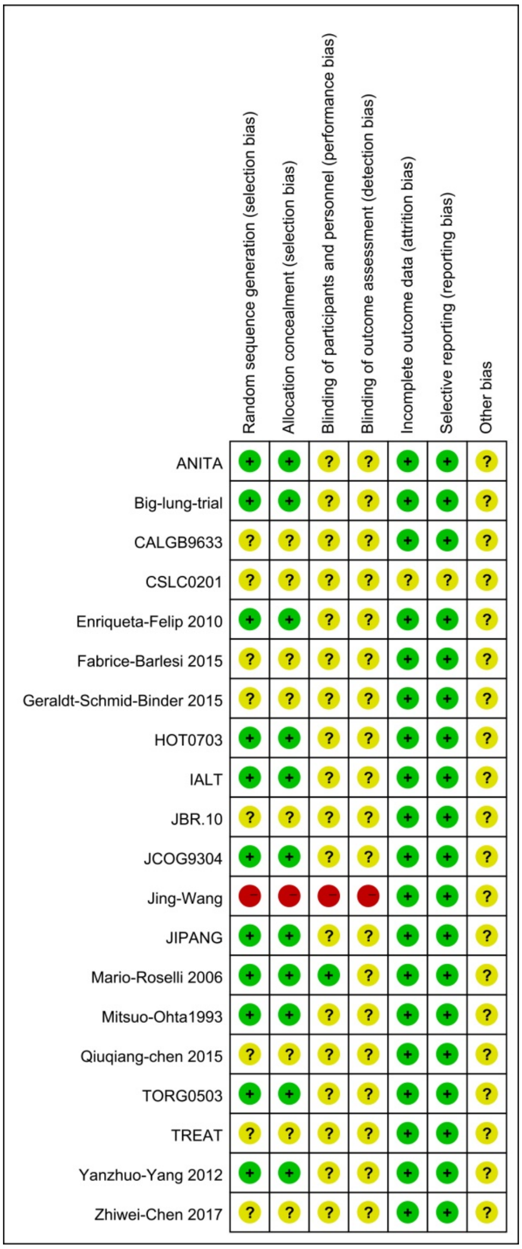

B) Risk of bias summary

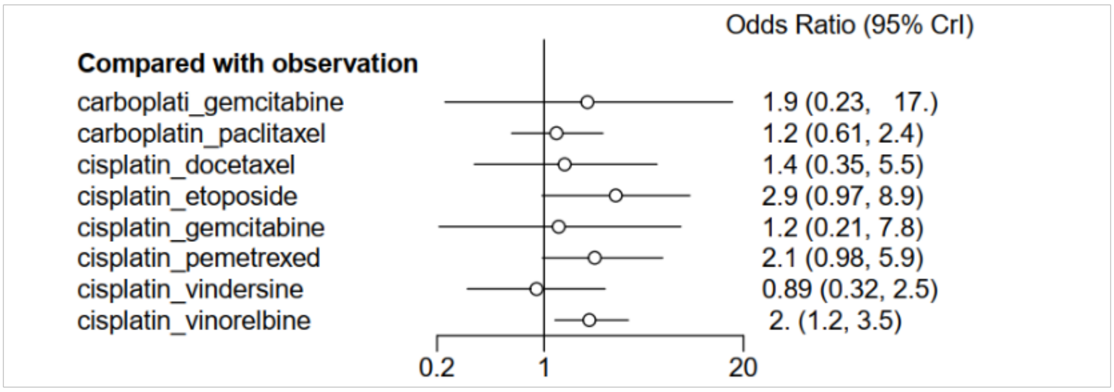

Supplementary.Figure 2.

A) Forest plots of the comparisons for the different cytotoxicity chemotherapy regimens with regard to 2-year RFS rate.

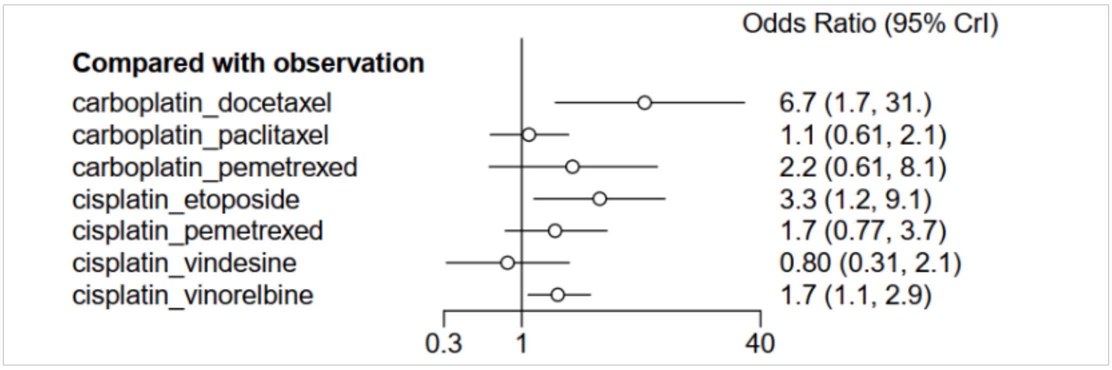

B) Forest plots of the comparisons for the different cytotoxicity chemotherapy regimens with regard to 3-year RFS rate.

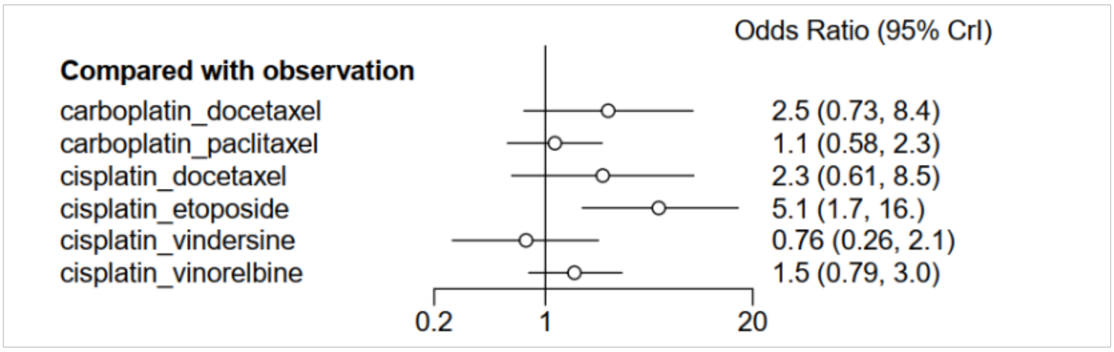

C) Forest plots of the comparisons for the different cytotoxicity chemotherapy regimens with regard to 5-year RFS rate.

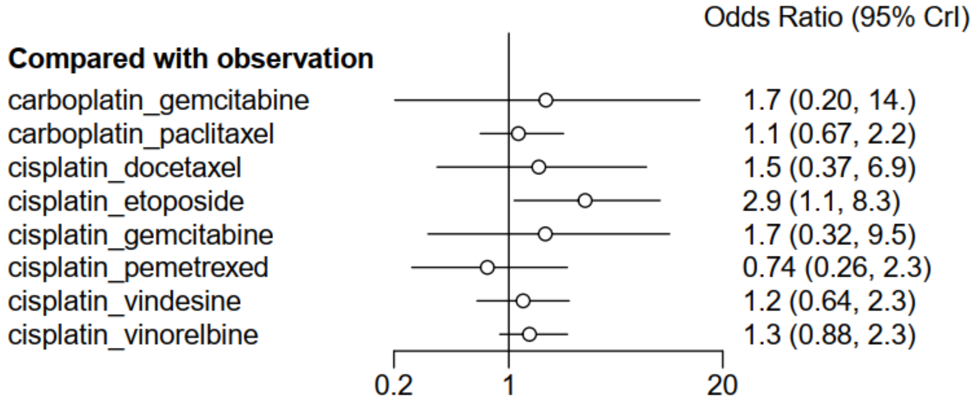

Supplementary.Figure 3.

A) Forest plots of the comparisons for the different cytotoxicity chemotherapy regimens with regard to 2-year OS rate.

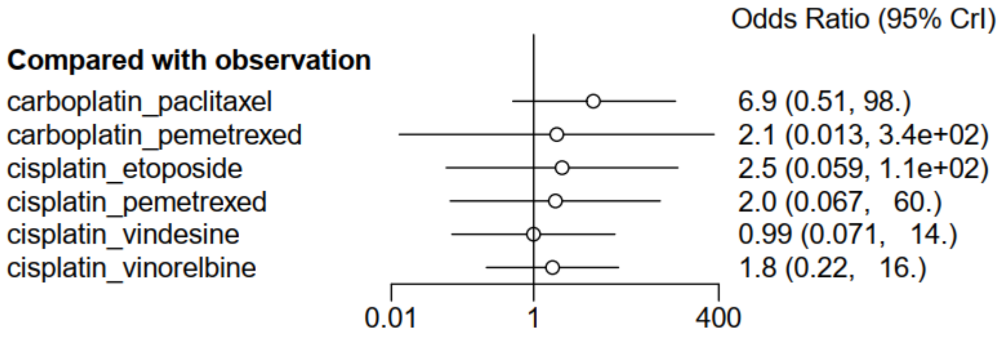

B) Forest plots of the comparisons for the different cytotoxicity chemotherapy regimens with regard to 3-year OS rate.

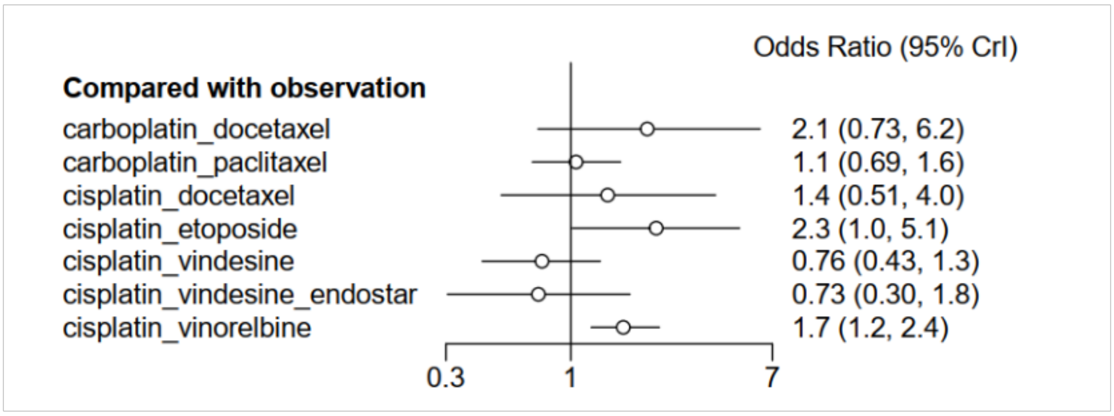

C) Forest plots of the comparisons for the different cytotoxicity chemotherapy regimens with regard to 5-year OS rate.

Supplementary.Material.2 The detailed searching strategy

*PubMed:

(“Carcinoma, non-small cell lung” OR “Lung carcinoma, non-small-cell” OR “Carcinoma, non-small cell lung” OR “Non-small cell lung cancer”)

AND (“adjuvant chemotherapy” OR “chemotherapy, adjuvant”)

AND (“platinum” OR “cisplatin” OR “carboplatin” or “nedaplatin” or “oxaliplatin”)

*EMBASE, and The Cochrane Library, Web of Science and Scopus Google Scholar:

(“Carcinoma, non-small cell lung” OR “Lung carcinoma, non-small-cell” OR “Carcinoma, non-small cell lung” OR “Non-small cell lung cancer”)

AND (“postoperative adjuvant chemotherapy”)

AND (“platinum” OR “cisplatin” OR “carboplatin” or “nedaplatin” or “oxaliplatin”)

In addition, searching performed in the database of Web of Science and Scopus Google Scholar was constrained to titles and abstracts while no limitations were applied in the PubMed, EMBASE, and The Cochrane Library.

For peer review only

PRISMA 2009 Checklist

Section/topic	#	Checklist item	Reported on page #
**TITLE**			
Title	1	Identify the report as a systematic review, meta-analysis, or both.	Page 1
**ABSTRACT**			
Structured summary	2	Provide a structured summary including, as applicable: background; objectives; data sources; study eligibility criteria, participants, and interventions; study appraisal and synthesis methods; results; limitations; conclusions and implications of key findings; systematic review registration number.	Page 3-5
**INTRODUCTION**			
Rationale	3	Describe the rationale for the review in the context of what is already known.	Page 6-7
Objectives	4	Provide an explicit statement of questions being addressed with reference to participants, interventions, comparisons, outcomes, and study design (PICOS).	Page 7-8
**METHODS**			
Protocol and registration	5	Indicate if a review protocol exists, if and where it can be accessed (e.g., Web address), and, if available, provide registration information including registration number.	NA
Eligibility criteria	6	Specify study characteristics (e.g., PICOS, length of follow-up) and report characteristics (e.g., years considered, language, publication status) used as criteria for eligibility, giving rationale.	Page 10
Information sources	7	Describe all information sources (e.g., databases with dates of coverage, contact with study authors to identify additional studies) in the search and date last searched.	Page 9
Search	8	Present full electronic search strategy for at least one database, including any limits used, such that it could be repeated.	Page 9
Study selection	9	State the process for selecting studies (i.e., screening, eligibility, included in systematic review, and, if applicable, included in the meta-analysis).	Page 10
Data collection process	10	Describe method of data extraction from reports (e.g., piloted forms, independently, in duplicate) and any processes for obtaining and confirming data from investigators.	Page 10-11
Data items	11	List and define all variables for which data were sought (e.g., PICOS, funding sources) and any assumptions and simplifications made.	Page 10-11
Risk of bias in individual studies	12	Describe methods used for assessing risk of bias of individual studies (including specification of whether this was done at the study or outcome level), and how this information is to be used in any data synthesis.	Page11

For peer review only

PRISMA 2009 Checklist

Summary measures	13	State the principal summary measures (e.g., risk ratio, difference in means).	Page 11-12
Synthesis of results	14	Describe the methods of handling data and combining results of studies, if done, including measures of consistency (e.g., I^2) for each meta-analysis.	Page 12

Section/topic	#	Checklist item	Reported on page #
Risk of bias across studies	15	Specify any assessment of risk of bias that may affect the cumulative evidence (e.g., publication bias, selective reporting within studies).	NA
Additional analyses	16	Describe methods of additional analyses (e.g., sensitivity or subgroup analyses, meta-regression), if done, indicating which were pre-specified.	NA
**RESULTS**			
Study selection	17	Give numbers of studies screened, assessed for eligibility, and included in the review, with reasons for exclusions at each stage, ideally with a flow diagram.	Page 13
Study characteristics	18	For each study, present characteristics for which data were extracted (e.g., study size, PICOS, follow-up period) and provide the citations.	Page 13-14
Risk of bias within studies	19	Present data on risk of bias of each study and, if available, any outcome level assessment (see item 12).	Page 14
Results of individual studies	20	For all outcomes considered (benefits or harms), present, for each study: (a) simple summary data for each intervention group (b) effect estimates and confidence intervals, ideally with a forest plot.	NA
Synthesis of results	21	Present results of each meta-analysis done, including confidence intervals and measures of consistency.	Page 14-19
Risk of bias across studies	22	Present results of any assessment of risk of bias across studies (see Item 15).	NA
Additional analysis	23	Give results of additional analyses, if done (e.g., sensitivity or subgroup analyses, meta-regression [see Item 16]).	NA
**DISCUSSION**			
Summary of evidence	24	Summarize the main findings including the strength of evidence for each main outcome; consider their relevance to key groups (e.g., healthcare providers, users, and policy makers).	Page 20-24
Limitations	25	Discuss limitations at study and outcome level (e.g., risk of bias), and at review-level (e.g., incomplete retrieval of identified research, reporting bias).	Page 24

For peer review only

For peer review only - http://bmjopen.bmj.com/site/about/guidelines.xhtml

PRISMA 2009 Checklist

Conclusions	26	Provide a general interpretation of the results in the context of other evidence, and implications for future research.	Page 25
**FUNDING**			
Funding	27	Describe sources of funding for the systematic review and other support (e.g., supply of data); role of funders for the systematic review.	Page 2

From: Moher D, Liberati A, Tetzlaff J, Altman DG, The PRISMA Group (2009). Preferred Reporting Items for Systematic Reviews and Meta-Analyses: The PRISMA Statement. PLoS Med 6(7): e1000097. doi:10.1371/journal.pmed1000097

For more information, visit: www.prisma-statement.org.

Page 2 of 2

For peer review only

Investigation of the Optimal Platinum-based Regimen in the Postoperative Adjuvant Chemotherapy Setting for Early-stage Resected Non-small Lung Cancer: A Bayesian Network Meta-analysis

Journal:	*BMJ Open*
Manuscript ID	bmjopen-2021-057098.R1
Article Type:	Original research
Date Submitted by the Author:	25-Feb-2022

Complete List of Authors:	pang, lanlan; Sun Yat-sen University Cancer Center, Department of Medical Oncology<br>Gan, Jiadi; Sun Yat-sen University Cancer Center, Department of Medical Oncology<br>Huang, Yi-Hua; Sun Yat-sen University Cancer Center, Department of Medical Oncology<br>Liao, Jun; Sun Yat-sen University Cancer Center, Department of Medical Oncology<br>Lv, Yi; Sun Yat-Sen University, Zhongshan School of Medicine<br>Ali, Wael; Sun Yat-sen University Cancer Center, Department of Medical Oncology<br>Zhang, L ; Sun Yat-sen University Cancer Center, Department of Medical Oncology<br>Fang, Wenfeng; Sun Yat-sen University Cancer Center, Department of Medical Oncology

**Primary Subject Heading**:	Oncology
Secondary Subject Heading:	Oncology
Keywords:	Adult oncology < ONCOLOGY, Respiratory tract tumours < ONCOLOGY, Thoracic surgery < SURGERY, CHEMOTHERAPY

For peer review only - http://bmjopen.bmj.com/site/about/guidelines.xhtml

I, the Submitting Author has the right to grant and does grant on behalf of all authors of the Work (as defined in the below author licence), an exclusive licence and/or a non-exclusive licence for contributions from authors who are: i) UK Crown employees; ii) where BMJ has agreed a CC-BY licence shall apply, and/or iii) in accordance with the terms applicable for US Federal Government officers or employees acting as part of their official duties; on a worldwide, perpetual, irrevocable, royalty-free basis to BMJ Publishing Group Ltd ("BMJ") its licensees and where the relevant Journal is co-owned by BMJ to the co-owners of the Journal, to publish the Work in this journal and any other BMJ products and to exploit all rights, as set out in our licence.

The Submitting Author accepts and understands that any supply made under these terms is made by BMJ to the Submitting Author unless you are acting as an employee on behalf of your employer or a postgraduate student of an affiliated institution which is paying any applicable article publishing charge ("APC") for Open Access articles. Where the Submitting Author wishes to make the Work available on an Open Access basis (and intends to pay the relevant APC), the terms of reuse of such Open Access shall be governed by a Creative Commons licence – details of these licences and which Creative Commons licence will apply to this Work are set out in our licence referred to above.

Other than as permitted in any relevant BMJ Author's Self Archiving Policies, I confirm this Work has not been accepted for publication elsewhere, is not being considered for publication elsewhere and does not duplicate material already published. I confirm all authors consent to publication of this Work and authorise the granting of this licence.

For peer review only

Investigation of the Optimal Platinum-based Regimen in the Postoperative Adjuvant Chemotherapy Setting for Early-stage Resected Non-small Lung Cancer: A Bayesian Network Meta-analysis

Running title: Postoperative Platinum-based Adjuvant Chemotherapy Setting for NSCLC： a Meta-analysis

Author:

Lan-Lan Pang,MD[1], Jia-Di Gan,MD[1], Yi-Hua Huang,MD[1], Jun Liao,MD[1], Yi Lv, MD[2], Wael-Abdullah -Sultan Ali, MD[1], Li Zhang, MD , Phd[1], Wen-Feng Fang, MD ,Phd[1#]

Institutions:

[1]Department of Medical Oncology, State Key Laboratory of Oncology in South China, Collaborative Innovation Center for Cancer Medicine, Sun Yat-sen University Cancer Center, Guangzhou 510060, People's Republic of China

[2] Zhongshan School of Medicine, Sun Yat-sen University, 74 Zhongshan II Road, Guangzhou510080, People's Republic of China.

#Correspondence:

Wenfeng Fang, MD, Phd, Department of Medical Oncology, State Key Laboratory of Oncology in South China, Collaborative Innovation Center for Cancer Medicine, Sun Yat-sen University Cancer Center, Guangzhou 510060, Peoples Republic of China. Email: fangwf@sysucc.org.cn.

Acknowledgments:

For peer review only

This work was financially supported by the Chinese National Natural Science Foundation Project (81772476). We thank all the co-authors for their contributions to this study.

Formatting of funding sources:

This work was supported by the Chinese National Natural Science Foundation Project (81772476).

For peer review only

Abstracts

Objective: The present study aimed to compare the efficacy and safety of different platinum adjuvant chemotherapy regimens for early-stage resected non-small-cell lung cancer (NSCLC).

Design: Systematic review with network meta-analysis (NMA) of randomized trials.

Data sources: PubMed, EMBASE, The Cochrane Library, Web of Science, and Scopus Google Scholar were searched through March 12th, 2021.

Eligibility criteria: Eligible randomized controlled trials comparing the postoperative platinum chemotherapy regimen with the observation-controlled group or comparing two platinum chemotherapy regimens head-to-head were included.

Data extraction and synthesis: The primary outcome was the efficacy of adjuvant chemotherapy regimens including relapse-free survival (RFS), overall survival (OS), 2, 3, 5-year RFS rate, and OS rate. The secondary outcome was the rate of grade3-4 toxicity assessments. Cochrane Handbook (Version 5) was utilized for the risk of bias assessment. Analyses were performed utilizing R-software version 4.3.1.

Results: 20 RCTs with a sample size of 5483 were enrolled in meta-analysis. The chemotherapy group had a significant RFS, and OS advantage compared to the observation group [hazard ratio (HR), 0.67; 95% confidence interval (CI), 0.56 to 0.81, P<0.0001; HR, 0.80; 95% CI, 0.73 to 0.88, P<0.0001, respectively]. Compared with the observation arm, only the “Cisplatin_Vinorelbine” regimen had a significant RFS and OS advantage (HR, 0.63; 95%CI, 0.43 to 0.87; HR, 0.74; 95%CI, 0.63 to 0.87, respectively) while the remaining chemotherapy regimens had no significant difference

For peer review only

of efficacy compared to the observation group. In terms of the safety of adjuvant chemotherapy, the incidence of hematological toxicities was not significantly higher in the "Cisplatin_Vinorelbine" arm than in any other chemotherapy group.

Conclusion: The current study summarized the adjuvant cytotoxicity chemotherapy regimens for patients with early-stage resected NSCLC. Our analysis may bring some guiding significance to the clinicians when they make clinical decisions of the optimal chemotherapy regimen.

Keywords: Non-small cell lung cancer, adjuvant chemotherapy, platinum-based, Network Meta-analysis

Trial registration: None

Strengths and limitations of this study

*The Bayesian Network Meta-analysis provided access to directly compare the efficacy and safety of different adjuvant chemotherapy regimens for NSCLC patients.

* It is difficult to determine the optimal subgroup populations who may obtain the benefit from certain cytotoxicity chemotherapy regimens.

* We did not incorporate any targeted or immune-biological therapy into the analysis due to the open-loop of interventions.

* The present study could not present a subgroup analysis stratified by postoperative radiotherapy or not as it was undertaken according to every center's policy.

For peer review only

Introduction:

Lung cancer is the most common cause of cancer-related deaths worldwide and patients with non-small lung cancer (NSCLC) account for almost 85% of all cases[1, 2]. Complete surgical resection is recognized as the standard treatment for patients with early-stage NSCLC. However, 30% to 70% of patients ultimately experience local or distant relapse and the five-year survival rate ranges from only 30% to 60%[3, 4]. The presence of micro-metastases, which could not be detected by conventional diagnostic techniques, increases the rate of relapse and exerts a negative impact on the survival of patients[5]. Therefore, adjuvant postoperative systematic therapy is necessary for patients experiencing surgical resection.

A pooled analysis conducted by the Lung Adjuvant Cisplatin Evaluation (LACE) Collaborative Group suggested that cisplatin-based adjuvant chemotherapy yield an absolute OS benefit of 5.40% at five years[6]. In light of this promising data, the American Society of Clinical Oncology (ASCO) guidelines recommended the cisplatin-based adjuvant postoperative chemotherapy regimen as the standard treatment for early-stage NSCLC patients[7]. However, the optimal adjuvant chemotherapy regimen has not yet been determined. “Cisplatin_Vinorelbine” has shown its promising efficacy compared with the observation group and is recognized as the standard adjuvant chemotherapy regimen currently[8, 9]. In addition, several RCTs have also investigated the feasibility, efficacy, and safety of cisplatin plus non-vinorelbine third-generation drug as the postoperative adjuvant chemotherapy [10-12]. Among them, “Cisplatin_Pemetrexed” is characterized as having promising efficacy and an

For peer review only

acceptable safety profile in comparison with “Cisplatin_vinorelbine”[12, 13]. Thus, despite the lack of level 1 data concerning the efficacy of cisplatin plus vinorelbine, it was still recommended by the National Comprehensive Cancer Network (NCCN) guidelines as the optional postoperative adjuvant chemotherapy regimen for non-squamous NSCLC[14]. Meanwhile, although carboplatin-based adjuvant regimens have not yet been recommended by guidelines, it might be a favorable choice for patients with comorbidities and unsuited to receive cisplatin when accompanied by the late toxicity [15, 16].

Recent advances in individualized treatment based on molecular and biological profiling have shaped the future of postoperative adjuvant chemotherapy. Substantial numbers of patients benefit from the corresponding adjuvant regimen. The patients unsuited to adjuvant cytotoxicity chemotherapy may benefit from the first-generation Tyrosine Kinase Inhibitor (TKI) but serval clinical trials showed poor outcomes of TKI adjuvant therapy until the advent of ADJUVANT study [17-19]. However, individualization could not apply to all NSCLC patients and those patients inapplicable to it have no choice but receive platinum adjuvant chemotherapy.

Postoperative adjuvant chemotherapy setting for early-stage resected NSCLC is of great significance for the oncological community in routine clinical practice. However, it seems difficult to conduct large RCT to figure out the preferred platinum chemotherapy regimen and utilizing the published data might be an alternative option. Hence, we conducted this systematic review and network meta-analysis (NMA) aiming

For peer review only

to compare the efficacy and safety of different platinum adjuvant chemotherapy regimens.

For peer review only

114 **Methods:**

115 This systematic review and NMA was performed in accordance with the Preferred
116 Reporting Items for Systematic reviews and Meta-analyses guidelines for RCTs (The
117 PRISMA Statement) (**Supplementary material 1**).

118 **Data Sources**

119 Two authors (L.L.P and J.D.G) independently searched the records in the electronic
120 database of PubMed, EMBASE, and The Cochrane Library, Web of Science and
121 Scopus Google Scholar. The searching terminal date was March 12th, 2021. Searching
122 terms were focused on NSCLC, adjuvant chemotherapy, and platinum. If necessary, an
123 additional manual search of related literature in the reference list would be carried out
124 to enroll any relevant publications. The datasets utilized in this analysis could be
125 obtained from the corresponding author upon request. Records were imported into
126 EndnoteX9 software to eliminate duplications. The detailed strategy was presented in
127 **Supplementary material 2**.

128 **Trial Selection Criteria and Trial Identification**

129 Two authors (L.L.P and Y.H.H) independently reviewed the title, abstracts, and
130 keywords of identified citations to select appropriate articles for a full review. Any
131 disagreement was resolved by consensus or through the judgement of a senior author
132 (W.F.F). Trials would be eligible only if meeting all the following criteria: 1) patients
133 with completely resected NSCLC (squamous and non-squamous) at stage IB–IIIA; 2)
134 Eligible RCTs comparing the postoperative platinum chemotherapy regimen with
135 observation-controlled group or those concerning two platinum chemotherapy

For peer review only

regimens head-to-head comparison; given that Vinorelbine, Etoposide, Pemetrexed, Docetaxel, Paclitaxel, Gemcitabine, Vindesine are currently commonly used in the routine clinical practice, the counterpart of the platinum doublet including these above-mentioned drugs were considered eligible; and a platinum triplet must be a platinum doublet combined with anti-angiogenesis drug; 3) full-text publications or conference abstract; 4) non-language restrictions. Publications would be disregarded if meeting any of the following criteria: 1) any single perioperative chemotherapy, neo-adjuvant chemotherapy, maintenance chemotherapy, or radio-chemotherapy; 2) chemo-naive patients with advanced, incurable, or recurrent NSCLC; 3) no RCTs, including reviews, case series or retrospective trials.

Outcomes and Data Extraction

Two authors (L.L.P and J.L) independently performed data extraction and any discrepancies were eliminated by consensus. Data for eligible trials related to basic characteristics were extracted, including: trial/author name, publication year, country of origin/multi-centers, sample size per group, chemotherapy regime, administration dose of each treatment and number of cycles, follow-up time, phases II/III, basic patients' characteristics (age, sex ratio, stage, pathology, performance status).

The primary outcome was the efficacy of adjuvant chemotherapy regimens including RFS, OS, 2, 3, 5-year RFS rate, and OS rate. RFS was defined as the interval from day of randomized assignment to disease recurrence or death, whichever occurred first, while OS referred to the time from randomization to death from any cause.

The secondary outcomes were the rate of grade3-4 toxicity assessment, including

For peer review only

158 hematological (anemia, neutropenia, febrile neutropenia, and thrombocytopenia) and
159 non-hematological (nausea/vomiting) adverse events. No additional information was
160 requested from the authors. Also, Parmar's method would be utilized to obtain survival
161 if necessary.

162 **Quality and Risk of Bias Assessment**

163 Two researchers (L.L.P and J.D.G) independently assessed the risk of bias of enrolled
164 trials according to the recommendations of the Cochrane Handbook of Systematic
165 Reviews of Interventions (Version 5, http://handbook.cochrane.org)[20]. Disagree-
166 ments were resolved by discussion.

167 **Data Synthesis and Analysis**

168 For RFS and OS, the logarithm of hazard ratio (HR) and their standard error (SE) were
169 pooled into analysis through a Bayesian multiple treatment network meta-analysis with
170 random effects. As for the dichotomous variables, odds ratio (OR) with 95% confidence
171 interval (CI) was applied to calculate. When a network diagram indicated two or more
172 independent loops, only the loop containing "Cisplatin_Vinorelbine" was selected for
173 further analysis.

174 Random effects and consistency model was computed utilizing Markov chain Monte
175 Carlo methods with Gibbs sampling based on simulations of 50000 iterations and 20000
176 adaptions in each of 4 chains. For a forest plot, "observation" was chosen as the
177 common reference comparator in the analysis of RFS and OS while
178 "Cisplatin_Vinorelbine" was applied in the analysis of chemotherapy toxicity. A league
179 table for the survival analysis was presented with the logarithm of HR and their 95%

For peer review only

180 CI. The surface under the cumulative ranking curve (SUCRA), represents the percentage of efficacy achieved by an agent compared to an imaginary agent that is always the best without uncertainty (ie, SUCRA = 100%)[21]. Namely, the SUCRA value would be 1 if treatment is certain to be the best and 0 if it is certain to be the worst. Higher SUCRA scores correspond to a higher ranking for extending survival.

All analyses in this article were performed utilizing R-software version 4.3.1 and the gemtc package version 0.8 that interfaces with JAGS version 4.3.0 for computing a Markov chain.

Patient and public involvement

Patients and the public were not involved in the design or planning of the study.

For peer review only

Results

The search process, study characteristics, and quality assessment

A total of 3432 records yielded after searching the database and 1991 records were left to be identified after removing 1692 duplications. Then, 1905 irrelevant citations were eliminated by skimming their title, abstracts, and keywords, leaving 86 articles to be considered potentially eligible. 69 articles were further excluded for irrelevant to topic (n=45); non-RCTs (n=11); non-accessible outcome (n=9); non-accessible text (n=4); and 17 articles were enrolled into analysis after skimming their full text. Additionally, three studies were included by browsing the references. Finally, 20 studies with a sample size of 5483 were enrolled into meta-analysis **(Figure 1)**.

Table 1 showed the basic characteristics of enrolled studies and participants. These studies were conducted in countries worldwide, most of which were Japan[22-26] and China[11, 27-29], followed by France[30, 31], Germany[32], Italy[33], USA[34], UK[35], and North Carolina[10]. Noteworthy, three of them were conducted in multi-countries[36-38]. 11 studies compared the postoperative platinum chemotherapy regimen with the observation-controlled group while the remaining nine studies comparing two platinum chemotherapy regimens head-to-head. Chemotherapy regimens included "Cisplatin_Vinorelbine", "Cisplatin_Pemetrexed", "Cisplatin _Gemcitabine", "Cisplatin_Vindesine", "Cisplatin_Etoposide", "Cisplatin_Docetaxel", "Carboplatin_Gemcitabine", "Carboplatin_Pemetrexed", "Carboplatin_Paclitaxel", "Carboplatin_Docetaxel_Endostar", "Carboplatin_Docetaxel", "Cisplatin_Vinorelbine _Endostar". The median follow-up time ranged from 20.2 to 116 months. Of note, we

have noticed four well-designed RCTs during the process of eligible trial selection[39-42]. Nevertheless, we could not obtain the specific outcomes in the subgroup populations per treatment regimens from these clinical trials, whereas only the outcomes concerning the comparison between the observation group and "Cisplatin _Vinorelbine" were available in BLT or IALT trial[31, 35]. Given that the objective of the present work was to investigate the optimal platinum-based regimen in the postoperative adjuvant chemotherapy setting for early-stage resected NSCLC, we only enrolled the BLT and IALT trial while leaving the remaining four RCTs out.

Based on the Cochrane Risk of Bias evaluation, one of the enrolled studies had a high risk of selection bias due to its quasi-randomized setting while nearly half the studies did not clarify the randomization methods in detail. One study had a high risk of performance bias while nearly all the studies did not mention utilizing the blind design or not. Attrition and reporting bias did not exist in the enrolled studies except one whose bias could not be evaluated due to a conference abstract (**Supplementary figure 1A-B**).

Primary outcome---RFS

10 studies were enrolled to analyze the RFS of platinum postoperative adjuvant chemotherapy compared with the observation group. We found that the chemotherapy group had a significant RFS advantage compared versus the observation group (HR, 0.67; 95%CI, 0.56 to 0.81; I^2=64%, P<0.0001) **(Figure 2A)**. Moreover, in terms of 2-year RFS rate, 3-year RFS rate and 5-year RFS rate, the chemotherapy arm presented higher RFS rate in comparison with the observation arm (OR=1.62, 95%CI 1.20 to 2.18,

I^2=68%, P=0.0016; OR=1.50, 95%CI 1.11 to 2.03, I^2=72%, P=0.0081; OR=1.49, 95%CI 1.09 to 2.04, I^2=69%, P=0.0132; respectively)**(Figure 2B-D)**.

15 clinical trials were enrolled to investigate the RFS of several chemotherapy regimens for patients with early stage resected NSCLC. In the Network evidence figure, each node represents a type of treatment. Solid lines connect treatments that are directly compared in at least one study. The thickness of connections varies based on the number of studies involved in a comparison. Network evidence of the comparisons for the best adjuvant chemotherapy concerning the RFS was shown in **Figure 3A**. Almost all platinum chemotherapy has a direct comparison with "Observation" except "Cisplatin_Docetaxel", "Cisplatin_Pemetrexed", "Cisplatin_Vinorelbine_Endostar" and "Carboplatin_Docetaxel_Endostar". Furthermore, the number of trials that compared "Cisplatin_Vinorelbine" versus "Observation" ranked the first. Furthermore, compared with the observation arm, only the "Cisplatin_Vinorelbine" regimen had a significant RFS advantage (HR, 0.63; 95%CI, 0.43 to 0.87) while there was no significant difference between the remaining chemotherapy regimens and the observation group---"Carboplatin_Docetaxel" (HR, 0.58; 95%CI, 0.24 to 1.4); "Carboplatin_Docetaxel_Endostar" (HR, 0.42; 95%CI, 0.05 to 3.8); "Carboplatin_ Paclitaxel" (HR, 0.88; 95%CI, 0.51 to 1.50); "Cisplatin_Docetaxel" (HR, 1.40; 95%CI, 0.49 to 3.90); "Cisplatin_Etoposide" (HR, 0.53; 95%CI, 0.23 to 1.30); " Carboplatin_Pemetrexed" (HR, 0.62; 95%CI, 0.29 to 1.20); "Cisplatin_Vindesine" (HR, 1.00; 95%CI, 0.45 to 2.20); "Cisplatin_Vinorelbine_Endostar" (HR, 0.60; 95%CI, 0.24 to 1.40) (**Figure 3B**). In addition, according to the league table, no significant

For peer review only

difference was observed in the remaining comparisons, eg “Cisplatin_ Vinorelbine” and “Cisplatin_Pemetrexmed”.(**Figure 3C**). In the rank of network meta-analysis, the biggest SUCRA value holds the potential to be the best chemotherapy regime in extending RFS. Our results showed that “Cisplatin_Etoposide” (72.0%) was the most effective chemotherapy regime to improve RFS, followed by “Carboplatin_Docetaxel_Endostar” (69.30%), “Carboplatin_Docetaxel” (65.31%), “Cisplatin_Vinorelbine” (64.73%), “Cisplatin_Pemetrexed” (64.2%), “Cisplatin _Vindesine” (64.2%), “Carboplatin_Paclitaxel” (36.3%), “Carboplatin_Vindesine” (27.6%), “Cisplatin_Vinorelbine_Endostar” (23.0%) and “Cisplatin_Docetaxel” (13.4%) (**Figure 3D**). In terms of the 2-year RFS rate, 3-year RFS rate and 5-year RFS rate, “Cisplatin_Vinorelbine” was an effective therapeutic method in improving 2-year RFS rate (OR=2.00, 95%CI 1.20 to 3.50); “Carboplatin_Docetaxel” (OR=6.70, 95%CI 1.70 to 31.00) “Cisplatin_Etoposide” (OR=3.30, 95%CI 1.20 to 9.10) and “Cisplatin_Vinorelbine” (OR=1.70, 95%CI 1.10 to 2.90) had a significantly higher 3-year RFS rate than observation arm; whereas “Cisplatin_Etoposide” (OR=5.10, 95%CI 1.70 to 16.00) was an effective method in improving 5-year RFS rate. However, there was no significant difference between the remaining chemotherapy regimens and observation arm concerning the RFS rate (**Supplementary figure 2A-C**).

Primary outcome--- OS

To compare the efficacy of platinum chemotherapy arm with the observation arm in extending OS, we enrolled 10 studies into analysis. Our results showed that the OS in the chemotherapy group was significantly longer than the observation group (HR, 0.80;

For peer review only

95%CI, 0.73 to 0.88; I^2=11%, P<0.0001) (**Figure 4A**). As for 2-year OS rate, 3-year OS rate and 5-year OS rate, the chemotherapy group showed a significant survival advantage than the observation group (OR=1.31, 95%CI 1.06 to 1.61, I^2=21%, P=0.0117; OR=2.24, 95% CI 1.13 to 4.45, I^2=94%, P=0.0081; OR=1.36, 95% CI 1.08 to 1.72, I^2=54%, P=0.0100; respectively)(**Figure 4B-D**).

Moreover, 14 clinical trials with eight treatment regimens were enrolled to investigate the optimum adjuvant chemotherapy regimens in extending OS. Network evidence of these chemotherapy regimens was shown in **Figure 5A**. Almost all platinum chemotherapy has a direct comparison with "Observation" except "Cisplatin_Docetaxel", "Cisplatin_Pemetrexed", and "Cisplatin_Vinorelbine_Endostar". Similar to RFS, the number of trials related to "Cisplatin_Vinorelbine" comparing versus "Observation" also ranked the first in extending OS. Compared with the observation arm, only the "Cisplatin_Vinorelbine" regimen had a significant OS advantage(HR, 0.74; 95%CI, 0.63 to 0.87) while there was no significant difference between the remaining chemotherapy regimens and the observation group---"Carboplatin_Paclitaxel" (HR,0.90; 95%CI, 0.70 to 1.20); "Cisplatin_Docetaxel" (HR, 1.10; 95%CI, 0.50 to 2.30) ; "Cisplatin_Etoposide" (HR, 0.80; 95% CI, 0.44 to 1.40); "Cisplatin_ Pemetrexed" (HR, 0.73; 95%CI, 0.47 to 1.10) ; "Cisplatin_Vindesine" (HR, 1.00; 95%CI, 0.69 to 1.40); "Cisplatin_Vinorelbine_Endostar" (HR, 0.75; 95%CI, 0.46 to 1.20) (**Figure 5B**). Similar to RFS, as the league table showed, there was no significant difference among these remaining platinum chemotherapy regimens in improving OS for patients with early-stage resected NSCLC (**Figure 5C**). In terms of

For peer review only

the rank of network meta-analysis, we found that "Cisplatin_Vinorelbine" (74.8%) was the most effective therapeutic arm for the improvement of OS, followed by "Cisplatin_Pemetrexed" (74.1%), "Cisplatin_Vinorelbine_Endostar" (68.5%), "Cisplatin_Etoposide" (59.2%), "Carboplatin_Paclitaxel" (43.6%), "Cisplatin_ Vindesine" (28.5%), "Cisplatin_Docetaxel" (27.0%) and "Observation" (24.3%) (**Figure 5D**). In addition, "Cisplatin_Etoposide" was an effective chemotherapy regimen to improve the 2-year OS rate (OR=2.90, 95% CI 1.10 to 8.30) and 5-year OS rate (OR= 6.70, 95% CI 1.70 to 3.10) while "Cisplatin_Vinorelbine" had a significantly higher 5-year OS rate than observation arm (OR=1.70, 95% CI 1.10 to 2.90). However, there was no significant difference between the remain chemotherapy regimens and observation arm concerning 2-year ,3-year and 5-year OS rate (**Supplementary figure 3A-C**).

Secondary outcome---anemia, neutropenia, thrombocytopenia, febrile neutropenia, and nausea/vomiting

15 clinical trials were enrolled to compare the presence of anemia among different adjuvant chemotherapy regimens. We found that the incidence of anemia in the "Carboplatin_Pemetrexed" arm was significantly higher than the " Cisplatin_Vinorelbine" arm (Log OR=44.06, 95% CI 0.21 to 151.05). However, there was no significant difference between the remaining chemotherapy regimens and "Cisplatin_Vinorelbine" (**Figure 6A**).

As for neutropenia, we enrolled 13 clinical trials to compare the safety of different adjuvant chemotherapy regimens. The neutropenia was more frequently observed in

patients receiving "Carboplatin_Gemcitabine" (Log OR= 60.60, 95%CI 0.94 to 193.7), "Cisplatin_Gemcitabine" (Log OR= 60.11, 95%CI 0.77 to 193.27), and "Cisplatin_Docetaxel" (Log OR= 59.71, 95%CI 0.56 to 192.89) than Cisplatin_Vinorelbine". However, the incidence of neutropenia in the "Cisplatin_Vinorelbine" group was not significantly higher than the remaining chemotherapy regimens group (**Figure 6B**).

Also, as for thrombocytopenia, we enrolled 11 clinical trials to investigate the safety of different adjuvant chemotherapy regimens. Patients medicated with "Carboplatin_Pemetrexed" were significantly observed thrombocytopenia compared with "Cisplatin_Vinorelbine" (Log OR=36.43, 95%CI 2.87 to 114.27). However, there was no significant difference between the remaining chemotherapy regimens and "Cisplatin_Vinorelbine" (**Figure 6C**).

In terms of febrile neutropenia, six clinical trials were included. Our results showed that there was no significant difference between these chemotherapy regimens and "Cisplatin_Vinorelbine" (**Figure 6D**).

In addition, 14 clinical trials were enrolled to analyze the observation of nausea/vomiting in NSCLC patients receiving postoperative adjuvant chemotherapy. We found that compared to "Cisplatin_Vinorelbine", "Carboplatin_Gemcitabine" (Log OR=45.59, 95%CI 1.29 to 154.91), "Cisplatin_Docetaxel" (Log OR=44.90, 95%CI 1.36 to 154.30), "Cisplatin_Gemcitabine" (Log OR=45.38, 95%CI 1.70 to 154.96) had a higher incidence of nausea/vomiting. However, the nausea/vomiting was more frequently observed in patients receiving "Cisplatin_Vinorelbine" than

For peer review only

344 "Carboplatin_Pemetrexed" (Log OR= -27.02, 95%CI -92.63 to -1.18). Furthermore, there was no significant difference between the remaining chemotherapy regimens and "Cisplatin_Vinorelbine" (**Figure 6E**).

For peer review only

Discussion

Recent advances in precision medicine have shaped the future of adjuvant therapy for patients with completely resected NSCLC [19]. However, for patients not applicable to targeted therapy or immunotherapy, cytotoxicity chemotherapy is still recommended by guidelines as the standard of care. It is of great significance to investigate the best platinum regimen in the postoperative adjuvant chemotherapy setting for early-stage NSCLC. Our results showed that adjuvant postoperative chemotherapy was beneficial to improve the RFS and OS compared with the observation arm. Among these chemotherapy regimens, the "Cisplatin_Vinorelbine" arm was an effective therapeutic method to improve survival with tolerable toxicity ; wherase the "Cisplatin_Pemetrexed" arm did not show advantage over the other therapeutic methods in improving survival. Also, the "Cisplatin_Etoposide" arm contributed to improving the RFS and OS rate for patients with resected NSCLC.

A meta-analysis, published in 1995, demonstrated that postoperative chemotherapy had a significant advantage in 5-year survival compared with the observation group, which opened up a new era of postoperative chemotherapy [43]. Of note, an updated analysis published in 2010 further confirmed the clinical value of postoperative adjuvant chemotherapy for patients with operable NSCLC, irrespective of adding radiotherapy or not[44]. Moreover, a pooled analysis also indicated the promising role of cisplatin-based adjuvant chemotherapy on the 5-year survival [6]. Based on these findings, it is universally acknowledged that adjuvant cytotoxicity chemotherapy integrating platinum and third-generation agents is conducive to the improvement of postsurgical

For peer review only

survival for NSCLC patients. In the present study, the survival benefit was also observed in the platinum adjuvant chemotherapy setting. The adjuvant chemotherapy regimens were not limited to cisplatin-based chemotherapy, which extended to cisplatin/carboplatin in combination with other third-generation agents. Furthermore, the peak recurrence of patients with early-stage NSCLC is 2 years later after surgery and a higher 5-year RFS rate represented the lower possibility of postoperative relapse. In our study, not only 5-year RFS and OS benefit but the improvement of RFS, OS, 2-year RFS and OS rate, and 3-year RFS and OS rate were observed in the adjuvant chemotherapy group. These significant survival advantages could be explained in part by the fact that postoperative adjuvant chemotherapy could decrease the presence of micro-metastases of cancer cells.

High heterogeneity existed in evaluating the RFS and OS rate between the chemotherapy and observation group. Given that heterogeneity in some analyses may impose a distinct effect on the synthesis results, the mechanisms behind deserve to be figured out. And more importantly, the heterogeneity may provide a guiding significance for the optimal subgroup of patients who may obtain the clinical benefits from postoperative adjuvant chemotherapy. As the JCOG9304[23] indicated, postoperative cisplatin with vindesine chemotherapy was not shown to be efficacious in cases of resected N2 NSCLC. Meanwhile, the JLCSSG trial also failed to elucidate the therapeutic benefits of postoperative cisplatin and vindesine chemotherapy, since the eligible patients in the trial were restrained to be stage III[25]. Therefore, it is

For peer review only

reasonable to assume that the benefits of adjuvant "Cisplatin_Vindesine" may be limited in patients with stage III (T3 or any N2).

According to the subgroup analysis conducted by LACE in 2010, "Cisplatin_Vinorelbine" was an effective therapeutic method for the improvement of survival with a manageable toxicity profile [8]. Over the few decades, the introduction of the other third-generation cytotoxicity chemotherapy regimens such as Pemetrexed and Docetaxel have brought into the development of platinum adjuvant chemotherapy [10-12]. Noteworthy, regardless of the absence of level 1 data in the adjuvant setting, due to its gold status in non-squamous advanced NSCLC with superior efficacy and low tolerability, "Cisplatin_Pemetrexed" is still reasonably recommended as a standard of care for patients with early-stage resectable non-squamous NSCLC [13, 14]. However, our results indicated that "Cisplatin_Pemetrexed" did not show its significant survival advantages over the observation arm in the adjuvant setting. In addition, a subgroup analysis conducted by a large clinical trial---ECOG1505, did not find a significant difference among the four platinum-based chemotherapy regimens: "Cisplatin_Vinorelbine", "Cisplatin_Docetaxel", "Cisplatin_Gemcitabine" and "Cisplatin_Pemetrexed" [45]. Of note, the absolute effect of adjuvant cytotoxicity chemotherapy on stage IB and squamous patients is still under debated [10, 46]. Hence, it seemed that the "Cisplatin_Vinorelbine" arm still played a vital role in the adjuvant setting for patients with NSCLC since its efficacy was not influenced by the histology or stage [8]. By contrast, the negative results of "Cisplatin_Pemetrexed" implies the restrictions of its application in the adjuvant chemotherapy setting for patients with

For peer review only

squamous. Moreover, some biomarkers such as the excision repair cross-complementation group 1 (ERCC1), b-Tubulin, and Mucin hold the potential to predict the efficacy of cytotoxicity chemotherapy in the adjuvant setting [47-49]. Therefore, the heterogeneity of biological characteristics of each patient imposed confounding factors to the comparisons of different chemotherapy regimens.

According to our analysis, "Cisplatin_Etoposide", the gold chemotherapy regimens for small cell lung cancer, did have a significantly higher survival rate over observation arm in the patients with early-stage resectable NSCLC. Mario Roselli et.al conducted a 10-year follow-up clinical trials to demonstrate the effect of "Cisplatin_Etoposide" on the reduction of recurrences and the extension of long-term survival, which enrolled only patients with stage I NSCLC and adopted the relatively high dose of Cisplatin (100mg/m2) and Etoposide (120mg/m2)[33]. Hence, considering the potential bias, the positive results of "Cisplatin_Etoposide" need to be interpreted with caution.

Anti-angiogenic therapy, such as bevacizumab and endostar, hold the potential to improve the efficacy of adjuvant chemotherapy probably through inhibiting the growth of micro-metastatic cancer cells and increasing the sensitivity of tumor cells to chemotherapy [27, 50, 51]. However, our results showed that the combination of chemotherapy regimens and endostar did not show advantage over a single platinum-based chemotherapy arm. Furthermore, according to the results of ECOG1505 trial, there was no significant difference between the adjuvant chemotherapy with or without bevacizumab for patients with resected NSCLC [45]. One of the reasons to explain these negative results might be the heterogeneity in the expression of vascular

For peer review only

endothelial growth factor (VEGF). Patients with high VEGF expression tend to obtain more benefits from anti-angiogenic therapy than the low VEGF expression ones [27]. In terms of the safety of adjuvant chemotherapy, the incidence of hematological toxicities and nausea/vomiting, which was greatly influenced by the dose and schedules, were not significantly higher in the "Cisplatin_Vinorelbine" arm than other chemotherapy. Noteworthy, the schedules in the adjuvant setting was quite different from the one utilized in the advanced NSCLC patients[52]. Therefore, "Cisplatin_Vinorelbine" could be recommended as an adjuvant cytotoxicity chemotherapy regimen with an acceptable safety profile. On the contrary, the incidence of anemia and thrombocytopenia in the "Carboplatin_Pemetrexed" group was significantly higher than the "Cisplatin_Vinorelbine" group. Both the "Carboplatin" and "Pemetrexed" are characterized as relatively safe cytotoxicity chemotherapy regimens and recommended to be utilized for patients with medical commodities. The contradictory outcomes could be explained in part by the relatively lower dose of "Cisplatin_Vinorelbine".

To the best of our knowledge, the current study was the first to directly compare the efficacy and safety of different adjuvant chemotherapy regimens for NSCLC patients. Despite the strengths, it was evident that there still existed some limitations in the present study. Firstly, for nearly half of the enrolled studies, the risk of bias in certain items remained unclear and the quality of them could not be evaluated. Secondary, due to the lack of specific data, the subgroup analysis stratified by stage and pathology could not be performed. Thus, it is difficult to determine the optimal subgroup populations

For peer review only

who may obtain the most benefits from certain cytotoxicity chemotherapy regimens. Furthermore, we did not incorporate any targeted or immune-biological therapy into the analysis due to the open-loop of interventions. Last but not at least, although the absolute effect of radiotherapy for patients with completely resected NSCLC was presented recently, we did not conduct a subgroup analysis stratified by postoperative radiotherapy or not as it was undertaken according to every center's policy.

Conclusion

In conclusion, the current study summarized the adjuvant cytotoxicity chemotherapy regimens for patients with early-stage resected NSCLC. Research on adjuvant cytotoxicity chemotherapy might be an out-of-date topic but numerous NSCLC patients could obtain benefit from the optimal cytotoxicity chemotherapy regimen. "Cisplatin_Vinorelbine" had a significant survival advantage with a relatively good safety profile in the adjuvant setting while the "Cisplatin_Pemetrexed" arm was not superior to the other therapeutic methods in improving survival. Our analysis may bring some guiding significance to the clinicians when they make clinical decisions of the optimal chemotherapy regimen.

For peer review only

For peer review only - http://bmjopen.bmj.com/site/about/guidelines.xhtml

Contributorship statement

Wenfeng Fang: Conceptualization; Lanlan Pang& Jiadi Gan & Yihua Huang: Data curation; Lanlan Pang & Jiadi Gan: Formal analysis; Wenfeng Fang: Funding acquisition; Lanlan Pang: Investigation; Lanlan Pang: Methodology; Wenfeng Fang: Project administration; Lanlan Pang: Resources; Lanlan Pang: Software, Li Zhang &Wenfeng Fang: Supervision; Jiadi Gan: Validation; Lanlan Pang: Visualization; All authors: Roles/Writing - original draft; All Authors: Writing - review & editing.

Competing Interest

The authors declare no potential conflicts of interest.

Funding

This work was supported by Chinese National Natural Science Foundation Project grant number 81772476.

Data sharing

No additional data available.

For peer review only

References

1. Sung, H., et al., *Global Cancer Statistics 2020: GLOBOCAN Estimates of Incidence and Mortality Worldwide for 36 Cancers in 185 Countries.* CA Cancer J Clin, 2021. **71**(3): p. 209-249.
2. Zappa, C. and S.A. Mousa, *Non-small cell lung cancer: current treatment and future advances.* Transl Lung Cancer Res, 2016. **5**(3): p. 288-300.
3. Mountain, C.F., *Revisions in the International System for Staging Lung Cancer.* Chest, 1997. **111**(6): p. 1710-7.
4. Goldstraw, P., et al., *The IASLC Lung Cancer Staging Project: Proposals for Revision of the TNM Stage Groupings in the Forthcoming (Eighth) Edition of the TNM Classification for Lung Cancer.* J Thorac Oncol, 2016. **11**(1): p. 39-51.
5. Rosell, R., et al., *The role of chemotherapy in early non-small-cell lung cancer management.* Lung Cancer, 2001. **34 Suppl 3**: p. S63-74.
6. Pignon, J.P., et al., *Lung adjuvant cisplatin evaluation: a pooled analysis by the LACE Collaborative Group.* J Clin Oncol, 2008. **26**(21): p. 3552-9.
7. *Cancer Care Ontario and American Society of Clinical Oncology Adjuvant Chemotherapy and Adjuvant Radiation Therapy for Stages I-IIIA Resectable Non-Small-Cell Lung Cancer Guideline.* J Oncol Pract, 2007. **3**(6): p. 332-335.
8. Douillard, J.Y., et al., *Adjuvant cisplatin and vinorelbine for completely resected non-small cell lung cancer: subgroup analysis of the Lung Adjuvant Cisplatin Evaluation.* J Thorac Oncol, 2010. **5**(2): p. 220-8.
9. Postmus, P.E., et al., *Early and locally advanced non-small-cell lung cancer (NSCLC): ESMO Clinical Practice Guidelines for diagnosis, treatment and follow-up.* Ann Oncol, 2017. **28**(suppl_4): p. iv1-iv21.
10. Strauss, G.M., et al., *Adjuvant paclitaxel plus carboplatin compared with observation in stage IB non-small-cell lung cancer: CALGB 9633 with the Cancer and Leukemia Group B, Radiation Therapy Oncology Group, and North Central Cancer Treatment Group Study Groups.* J Clin Oncol, 2008. **26**(31): p. 5043-51.
11. Chen, Q., et al., *Clinical observation of docetaxel or gemcitabine combined with cisplatin in the chemotherapy after surgery for stage II-III non-small cell lung cancer.* Pak J Med Sci, 2015. **31**(5): p. 1095-8.
12. Kreuter, M., et al., *Randomized phase 2 trial on refinement of early-stage NSCLC adjuvant chemotherapy with cisplatin and pemetrexed versus cisplatin and vinorelbine: the TREAT study.* Ann Oncol, 2013. **24**(4): p. 986-92.
13. Scagliotti, G.V., et al., *Phase III study comparing cisplatin plus gemcitabine with cisplatin plus pemetrexed in chemotherapy-naive patients with advanced-stage non-small-cell lung cancer.* J Clin Oncol, 2008. **26**(21): p. 3543-51.
14. Bray, F., et al., *Global cancer statistics 2018: GLOBOCAN estimates of incidence and mortality worldwide for 36 cancers in 185 countries.* CA Cancer J Clin, 2018. **68**(6): p. 394-424.
15. Ardizzoni, A., et al., *Cisplatin- versus carboplatin-based chemotherapy in first-line treatment of advanced non-small-cell lung cancer: an individual patient data meta-analysis.* J Natl Cancer Inst, 2007. **99**(11): p. 847-57.
16. Gridelli, C., et al., *Chemotherapy of advanced NSCLC in special patient population.* Ann Oncol,

For peer review only

2006. **17 Suppl 5**: p. v72-8.

17. Goss, G.D., et al., *Gefitinib versus placebo in completely resected non-small-cell lung cancer: results of the NCIC CTG BR19 study.* J Clin Oncol, 2013. **31**(27): p. 3320-6.

18. Kelly, K., et al., *Adjuvant Erlotinib Versus Placebo in Patients With Stage IB-IIIA Non-Small-Cell Lung Cancer (RADIANT): A Randomized, Double-Blind, Phase III Trial.* J Clin Oncol, 2015. **33**(34): p. 4007-14.

19. Zhong, W.Z., et al., *Gefitinib versus vinorelbine plus cisplatin as adjuvant treatment for stage II-IIIA (N1-N2) EGFR-mutant NSCLC (ADJUVANT/CTONG1104): a randomised, open-label, phase 3 study.* Lancet Oncol, 2018. **19**(1): p. 139-148.

20. Higgins, J.P.T., *Cochrane handbook for systematic reviews of interventions. Cochrane Collaboration.* 2011. **10**.

21. Salanti, G., A.E. Ades, and J.P. Ioannidis, *Graphical methods and numerical summaries for presenting results from multiple-treatment meta-analysis: an overview and tutorial.* J Clin Epidemiol, 2011. **64**(2): p. 163-71.

22. Fukumoto, S.I., et al., *A randomized phase II trial of cisplatin plus gemcitabine versus carboplatin plus gemcitabine in patients with completely resected non-small cell lung cancer: Hokkaido Lung Cancer Clinical Study Group Trial (HOT0703).* Cancer Chemother Pharmacol, 2020. **86**(1): p. 117-127.

23. Tada, H., et al., *A randomized trial comparing adjuvant chemotherapy versus surgery alone for completely resected pN2 non-small cell lung cancer (JCOG9304).* Lung Cancer, 2004. **43**(2): p. 167-73.

24. Kenmotsu, H., et al., *Randomized Phase III Study of Pemetrexed Plus Cisplatin Versus Vinorelbine Plus Cisplatin for Completely Resected Stage II to IIIA Nonsquamous Non-Small-Cell Lung Cancer.* J Clin Oncol, 2020. **38**(19): p. 2187-2196.

25. Ohta, M., et al., *Adjuvant chemotherapy for completely resected stage III non-small-cell lung cancer. Results of a randomized prospective study. The Japan Clinical Oncology Group.* J Thorac Cardiovasc Surg, 1993. **106**(4): p. 703-8.

26. Kubota, K., et al., *Randomized phase II trial of adjuvant chemotherapy with docetaxel plus cisplatin versus paclitaxel plus carboplatin in patients with completely resected non-small cell lung cancer: TORG 0503.* Lung Cancer, 2020. **141**: p. 32-36.

27. Chen, Z., et al., *Endostar in combination with postoperative adjuvant chemotherapy prolongs the disease free survival of stage IIIA NSCLC patients with high VEGF expression.* Oncotarget, 2017. **8**(45): p. 79703-79711.

28. Zhong, W.Z., et al., *Randomized Study of Adjuvant Docetaxel vs. Observation for Completely Resected Stage IB-IIIa NSCLC with 11 Years' Median Follow-Up.* Journal of Thoracic Oncology, 2017. **12**(1): p. S642-S643.

29. Park, J.H., et al., *Postoperative adjuvant chemotherapy for stage I non-small cell lung cancer.* Eur J Cardiothorac Surg, 2005. **27**(6): p. 1086-91.

30. Barlesi, F., et al., *A randomized trial comparing adjuvant chemotherapy with gemcitabine plus cisplatin with docetaxel plus cisplatin in patients with completely resected non-small-cell lung cancer with quality of life as the primary objective.* Interact Cardiovasc Thorac Surg, 2015. **20**(6): p. 783-90.

31. Dunant, A., J.P. Pignon, and T. Le Chevalier, *Adjuvant chemotherapy for non-small cell lung cancer: contribution of the International Adjuvant Lung Trial.* Clin Cancer Res, 2005. **11**(13 Pt

For peer review only

2): p. 5017s-5021s.

32. Kreuter, M., et al., *Three-Year Follow-Up of a Randomized Phase II Trial on Refinement of Early-Stage NSCLC Adjuvant Chemotherapy with Cisplatin and Pemetrexed versus Cisplatin and Vinorelbine (the TREAT Study).* J Thorac Oncol, 2016. **11**(1): p. 85-93.

33. Roselli, M., et al., *Postsurgical chemotherapy in stage IB nonsmall cell lung cancer: Long-term survival in a randomized study.* Int J Cancer, 2006. **119**(4): p. 955-60.

34. Winton, T., et al., *Vinorelbine plus cisplatin vs. observation in resected non-small-cell lung cancer.* N Engl J Med, 2005. **352**(25): p. 2589-97.

35. Waller, D., et al., *Chemotherapy for patients with non-small cell lung cancer: the surgical setting of the Big Lung Trial.* Eur J Cardiothorac Surg, 2004. **26**(1): p. 173-82.

36. Douillard, J.Y., et al., *Adjuvant vinorelbine plus cisplatin versus observation in patients with completely resected stage IB-IIIA non-small-cell lung cancer (Adjuvant Navelbine International Trialist Association [ANITA]): a randomised controlled trial.* Lancet Oncol, 2006. **7**(9): p. 719-27.

37. Schmid-Bindert, G., et al., *A randomized Phase 2 study of pemetrexed in combination with cisplatin or carboplatin as adjuvant chemotherapy in patients with completely resected stage IB or II Non-Small-Cell Lung Cancer.* Lung Cancer, 2015. **90**(3): p. 397-404.

38. Felip, E., et al., *Preoperative chemotherapy plus surgery versus surgery plus adjuvant chemotherapy versus surgery alone in early-stage non-small-cell lung cancer.* J Clin Oncol, 2010. **28**(19): p. 3138-45.

39. *A randomized controlled study of postoperative adjuvant chemoimmunotherapy of resected non-small cell lung cancer with IL2 and LAK cells: H.Kimura, Y.Yamaguchi, T.Fujisawa, M.Baba, and M.Shiba Institute of Pulmonary Cancer Research, School of Medicine, Chi %J Lung Cancer.* 1991.

40. Park, J.H., *P2-202: Postoperative adjuvant therapy for stage IIIA non small cell lung cancer.* Journal of Thoracic Oncology, 2007. **2**(8, Supplement): p. S651.

41. Park, J.H., et al., *Postoperative adjuvant chemotherapy for stage I non-small cell lung cancer ☆.* European Journal of Cardio-Thoracic Surgery, 2005. **27**(6): p. 1086-1091.

42. Scagliotti, G.V., et al., *Randomized study of adjuvant chemotherapy for completely resected stage I, II, or IIIA non-small-cell Lung cancer.* J Natl Cancer Inst, 2003. **95**(19): p. 1453-61.

43. *Chemotherapy in non-small cell lung cancer: a meta-analysis using updated data on individual patients from 52 randomised clinical trials. Non-small Cell Lung Cancer Collaborative Group.* Bmj, 1995. **311**(7010): p. 899-909.

44. Arriagada, R., et al., *Adjuvant chemotherapy, with or without postoperative radiotherapy, in operable non-small-cell lung cancer: two meta-analyses of individual patient data.* Lancet, 2010. **375**(9722): p. 1267-77.

45. Wakelee, H.A., et al., *Adjuvant chemotherapy with or without bevacizumab in patients with resected non-small-cell lung cancer (E1505): an open-label, multicentre, randomised, phase 3 trial.* Lancet Oncol, 2017. **18**(12): p. 1610-1623.

46. Scagliotti, G., et al., *The differential efficacy of pemetrexed according to NSCLC histology: a review of two Phase III studies.* Oncologist, 2009. **14**(3): p. 253-63.

47. Friboulet, L., et al., *ERCC1 isoform expression and DNA repair in non-small-cell lung cancer.* N Engl J Med, 2013. **368**(12): p. 1101-10.

48. Graziano, S.L., et al., *Prognostic significance of mucin and p53 expression in stage IB non-small*

For peer review only

cell lung cancer: a laboratory companion study to CALGB 9633. J Thorac Oncol, 2010. **5**(6): p. 810-7.

49. Reiman, T., et al., *Cross-validation study of class III beta-tubulin as a predictive marker for benefit from adjuvant chemotherapy in resected non-small-cell lung cancer: analysis of four randomized trials.* Ann Oncol, 2012. **23**(1): p. 86-93.

50. Folkman, J., *Antiangiogenesis in cancer therapy--endostatin and its mechanisms of action.* Exp Cell Res, 2006. **312**(5): p. 594-607.

51. Limaverde-Sousa, G., C. Sternberg, and C.G. Ferreira, *Antiangiogenesis beyond VEGF inhibition: a journey from antiangiogenic single-target to broad-spectrum agents.* Cancer Treat Rev, 2014. **40**(4): p. 548-57.

52. Caffo, O., et al., *An evaluation of the pharmacokinetics and clinical use of vinorelbine for NSCLC treatment.* Expert Opin Drug Metab Toxicol, 2013. **9**(8): p. 1037-51.

For peer review only

Figure list

Figure 1. Flow diagram of enrolled studies selection.

Figure 2.

A) The efficacy of platinum-based postoperative adjuvant chemotherapy in improving the RFS compared with the observation group.

B) 2-year RFS rate of the chemotherapy arm in comparison with the observation arm.

C) 3-year RFS rate of the chemotherapy arm in comparison with the observation arm.

D) 5-year RFS rate of the chemotherapy arm in comparison with the observation arm

Figure 3.

A) Network evidence of the comparisons for the best adjuvant chemotherapy concerning RFS. In the Network evidence figure, each node represents a type of treatment. Solid lines connect treatments that are directly compared in at least one study. The thickness of connections varies based on the number of studies involved in a comparison.

B) Forest plots of the comparisons for the different cytotoxicity chemotherapy regimens concerning RFS.

C) The league table of the comparisons for the different cytotoxicity chemotherapy regimens concerning RFS.

D) SUCRA value of every cytotoxicity chemotherapy regimens concerning RFS.

Figure 4.

A) The efficacy of platinum-based postoperative adjuvant chemotherapy in improving the OS compared with the observation group.

For peer review only

B) 2-year OS rate of the chemotherapy arm compared to the observation arm.

C) 3-year OS rate of the chemotherapy arm compared to the observation arm.

D) 5-year OS rate of the chemotherapy arm compared to the observation arm.

Figure 5.

A) Network evidence of the comparisons for the best adjuvant chemotherapy concerning OS.

B) Forest plots of the comparisons for the different cytotoxicity chemotherapy regimens concerning OS.

C) The league table of the comparisons for the different cytotoxicity chemotherapy regimens concerning OS.

D) SUCRA value of every cytotoxicity chemotherapy regimens concerning OS.

Figure 6.

A) Forest plots of the comparisons for the different cytotoxicity chemotherapy regimens concerning the presence of anemia.

B) Forest plots of the comparisons for the different cytotoxicity chemotherapy regimens concerning the presence of neutropenia.

C) Forest plots of the comparisons for the different cytotoxicity chemotherapy regimens concerning the presence of thrombocytopenia .

D) Forest plots of the comparisons for the different cytotoxicity chemotherapy regimens concerning the presence of febrile neutropenia.

E) Forest plots of the comparisons for the different cytotoxicity chemotherapy regimens concerning the presence of nausea/vomiting.

For peer review only

Supplementary Figure list

Supplementary Figure 1.

A) Risk of bias graph.

B) Risk of bias summary

Supplementary Figure 2.

A) Forest plots of the comparisons for the different cytotoxicity chemotherapy regimens with regard to 2-year RFS rate.

B) Forest plots of the comparisons for the different cytotoxicity chemotherapy regimens with regard to 3-year RFS rate.

C) Forest plots of the comparisons for the different cytotoxicity chemotherapy regimens with regard to 5-year RFS rate.

Supplementary Figure 3.

A) Forest plots of the comparisons for the different cytotoxicity chemotherapy regimens with regard to 2-year OS rate.

B) Forest plots of the comparisons for the different cytotoxicity chemotherapy regimens with regard to 3-year OS rate.

C) Forest plots of the comparisons for the different cytotoxicity chemotherapy regimens with regard to 5-year OS rate.

For peer review only

Table 1 Basic characteristics of enrolled studies and participants.

Trail	Publication year	Country of region	Chemotherapy regimes	Sample size	Median follow-up (months)	Phase	Radiotherapy	Patients characteristics (Pathology;stage,PS)	Age	Sex
ANITA	2006	Multi-Center	**Cisplatin**100mg/m2+**Vinorelbine**30mg/m2 VS.**Observation**	840(407vs.433)	76	III	Not mandatory.	NSCLC; Stage IB – IIIA	59 (32 – 75) VS.59 (18 – 75)	85%vs.87%
Big-lung-trial	2003	UK	**Cisplatin**80mg/m2+**Vinorelbine**30mg/m2 VS.**Observation**	66(37VS.29)	58.8	---	Not mandatory	NSCLC;Stage I – III	/	/
CALGB 9633	2004	North Carolina	**Carboplatin**(AUC-6) + **Paclitaxel** 200 mg/m2 VS. **Observation**	344(173VS.171)	74	III	Not-mentioned	NSCLC; T2 with pathologically negative lymph nodes	61(34-78) VS.62(40-81) (range)	65%VS.63%
CSLC0201	2016	China	**Carboplatin**+**Docetaxel** VS.**Observation**	82(43vs.39)	132	---	Not-mentioned	NSCLC; Stage IB-IIIA	/	/
NATCH	2010	Multi-Center	**Carboplatin**(AUC-6.0)**+Paclitaxel**200mg/m2 VS.**Observation**	423(211VS.212)	51	III	Postoperative radiotherapy was allowed in patients with pathologic N2 disease.	NSCLC ; Stage IA with tumor size more than 2 cm, IB, II, or T3N1	64(33-81) VS.64(36-89) (range)	86%VS.88%
Fabrice Barlesi et.al	2015	France	**Cisplatin**75mg/m2+**Gemcitabine**1250mg/m2VS. **Cisplatin**75mg/m2+**Docetaxel**75mg/m2	136(67VS.69)	20.2	---	Not mandatory.	NSCLC; Stage IB-III	57 (44 – 74) VS.57 (36 – 71)	75%vs.74%
HOT0703	2020	Japan	**Cisplatin**40mg/m2+**Gemcitabine**1000mg/m2 VS.**Carboplatin**(AUC-5)+**Gemcitabine** 1000mg/m2	102(51vs.51)	69.6	II	Not-mentioned	NSCLC; Stage IB-IIIA	63(40-72) VS.64(36-74) (range)	67%VS.63%
IALT	2004	France	**Cisplatin**100mg/m2+**Vinorelbine**30mg/m2VS .**Observation**	500(248VS.262)	49.2	---	Not allowed.	NSCLC; Stage I, II, or III	/	/
JBR10	2005;2010	North American	**Cisplatin**+**Vinorelbine**VS. **Observation**	482(240vs.242)	111.6	III	Not-mentioned	NSCLC; Stage IB (T2N0) or II (T1-2N1)	61(35-82) VS.60.5(34-78)	64%VS.66%

For peer review only

JCOG9304	2003	Japan	**Cisplatin**80mg/m2+**Vindesine**3mg/m2 VS.**Observation**	159(59VS.60)	---	---	Not-mentioned	NSCLC;N2	62 (41—75) VS.62 (43—74)	68%VS.62%
Jing Wang et.al	2012	China	**Cisplation**80mg/m2+**Vinorelbine**30mg/m2 VS.**Observation**	451(225VS.226)	46	---	Not-mentioned	NSCLC;Stage I, II, and IIIA	55(38-83) VS.58(38-82)	71%VS.75%
JIPANG	2020	Japan	**Cisplatin**75mg/m2+**Pemetrexed**500mg/m2VS. **Cisplatin**80mg/m2+**Vinorelbine**25mg/m2	804(402VS.402)	45.2	III	Not allowed.	Non-squamous NSCLC; N2 Stage II or IIIA	65(58-69) VS. 64 (57-67)	60%VS.58%
Mario Roselli et.al	2006	Italy	**Cisplatin**100mg/m2+**Etoposide**120mg/m2VS. **Observation**	140(70VS.70)	40.31	---	Not-mentioned	NSCLC;Stage IB disease (pT2N0)	64.7±9.9 VS.62.9±9.2	91%VS.76%
JLCSSG	1993	Japan	**Cisplatin**80mg/m2+**Vindesine** 3mg/m2VS.**Observation**	181(90VS.91)	31.2	---	Not-mentioned	NSCLC; Stage III	56.3 ± 9.1 VS.58.9 ± 8.4	77%S. 87%
Qiuqiang Chen et.al	2015	China	**Cisplatin**75mg/m2+**Docetaxel**75mg/m2VS. **Cisplatin**75mg/m2+**Gemcitabine**1250mg/m2	92(45vs.47)	22	---	Not-mentioned	NSCLC; Stage II to III	55(32-67) VS.56(31-67) (range)	84%VS.87%
Schmid-Binder Geraldt et.al	2015	Germany, France, and Spain	**Cisplatin**75mg/m2+**Pemetrexed**500mg/m2VS. **Carboplatin**(AUC-5)+**Pemetrexed** 500 mg/m2	112(63VS.59)	---	II	Not-mentioned	NSCLC; Stage IB, IIA or IIB	61(44-75) VS.59(43-69)	78%VS.70%
TORG0503	2019	Japan	**Cisplatin**80mg/m2+**Docetaxel**60mg/m2VS. **Carboplatin**AUC-6+**Paclitaxel** 200 mg/m2	111(58vs.53)	---	II	Not-mentioned	NSCLC; Stage IB, II and IIIA	63(33-70) VS.59(34-70)	60%VS.66%
TREAT	2015	German	**Cisplatin**50mg/m2+**Vinorelbine**25mg/m2VS. **Cisplatin**75mg/m2+**Pemetrexed**500mg/m2	132(67vs.65)	36m	II	Not allowed.	NSCLC; Stages IB, IIA, IIB,	58 (40－73) VS.60 (38－74)	72%VS. 77%
Yanzhuo Yang et.al	2012	China	**Carboplatin**(AUC=5-6)+**Docetaxel**75mg/m2+ **Endostar**15mgVS.**Carboplatin**+**Docetaxel**	76(38vs.38)	22	---	Not-mentioned	NSCLC;Stage IB-III	55.6(36-74) VS.60.2(45-77)	31%vs.27%
Zhiwei Chen et.al	2017	China	**Cisplatin**75mg/m2+**Vinorelbine**25mg/m2+ **Endostar**7.5mg/m2VS.**Cisplatin**+**Vinorelbine**	250(125vs.125)	60	III	Not-mentioned	NSCLC; Stage IB to IIIA	58(33-75) VS.55.5(37-71)	66%VS.67%

NSCLC: non-small cell lung cancer; PS: performance status; AUC: area under the curve

For peer review only

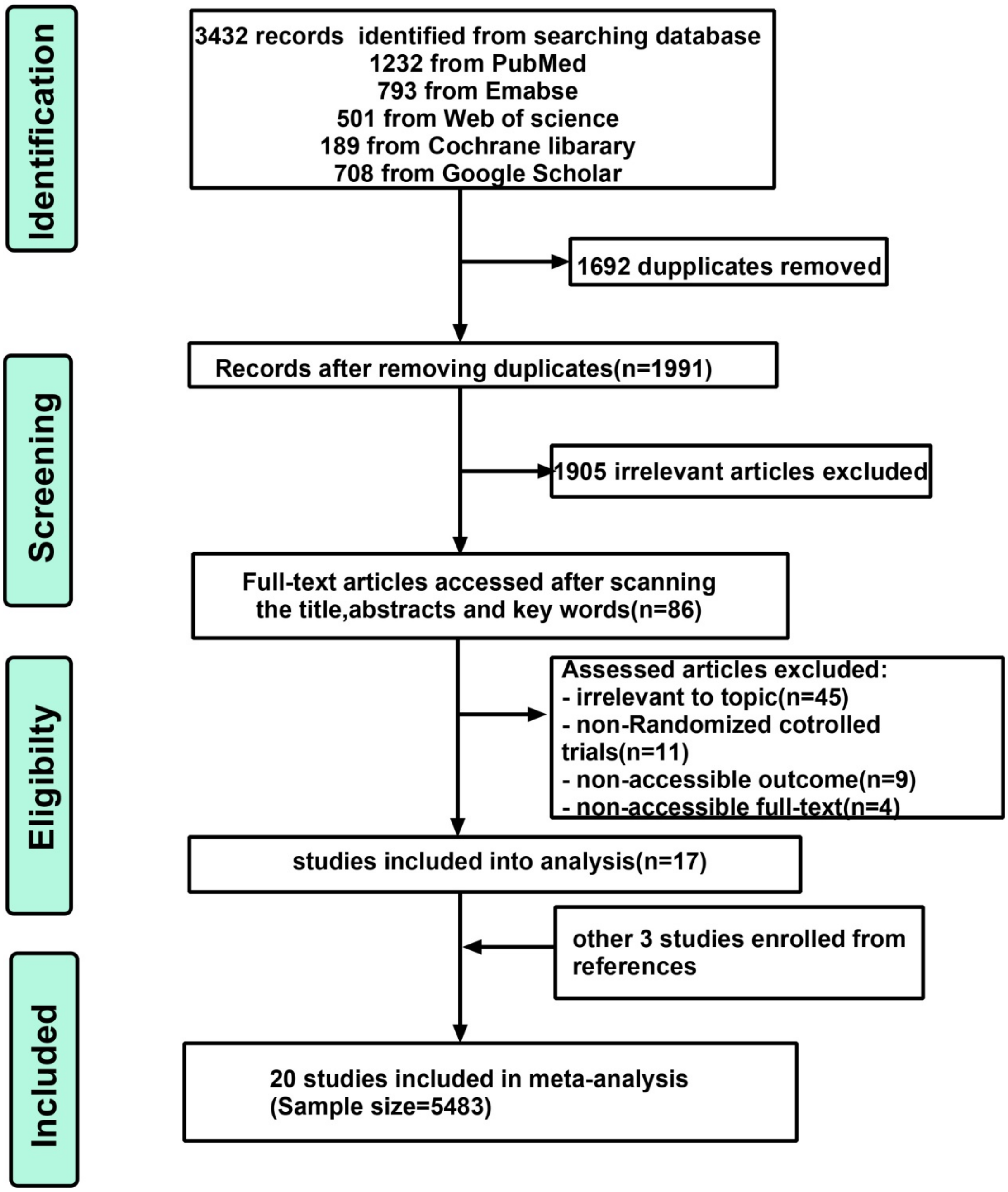

Figure 1. Flow diagram of enrolled studies selection.

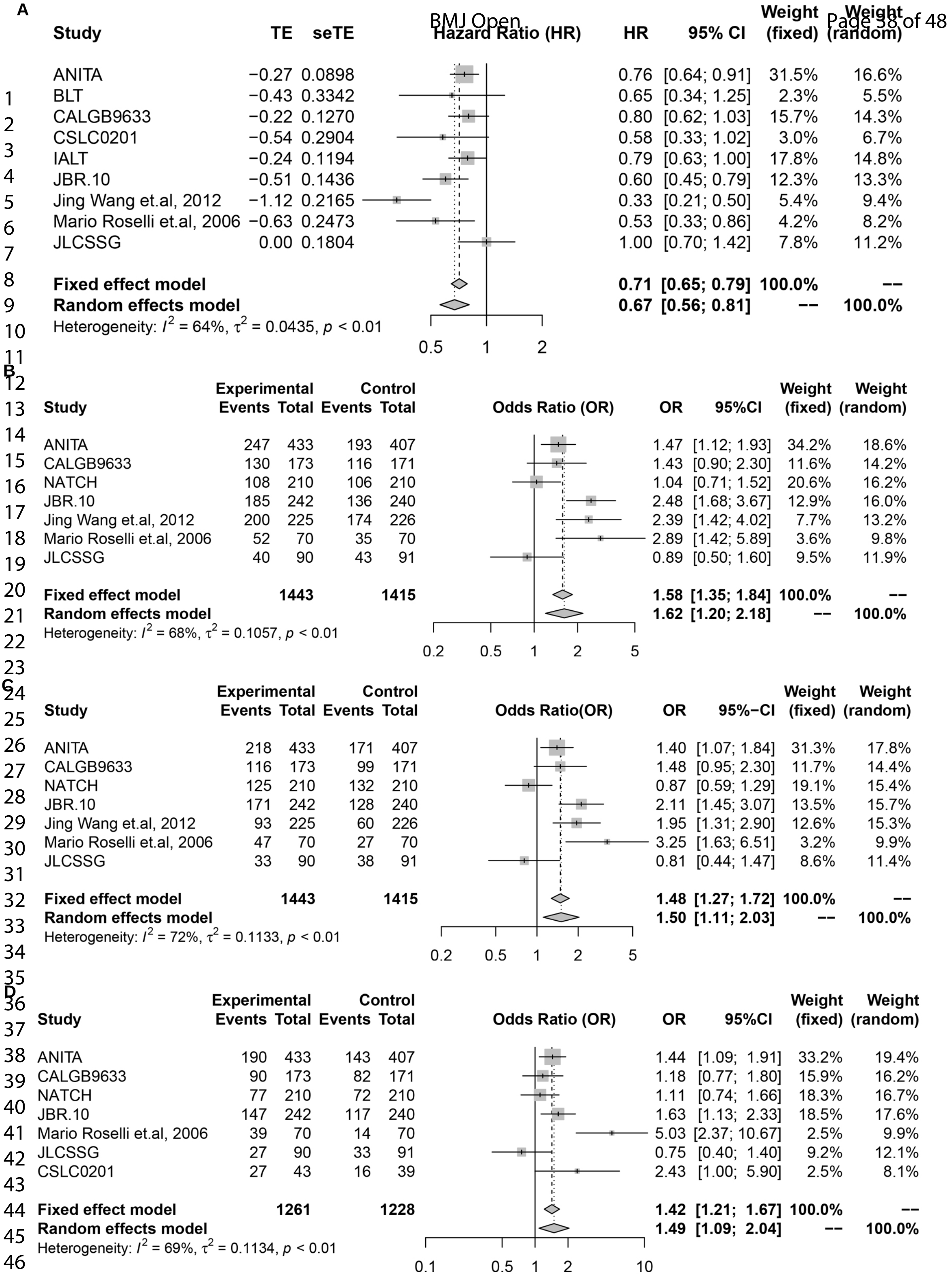

Figure 2.
A) The efficacy of platinum-based postoperative adjuvant chemotherapy in improving the relapse-free survival (RFS) compared with the observation group.
B) 2-year; **C)** 3-year ; **D)** 5-year RFS rate of the chemotherapy arm in comparison with the observation arm.

A

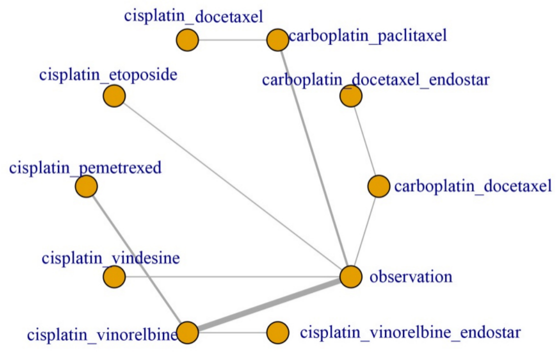

B

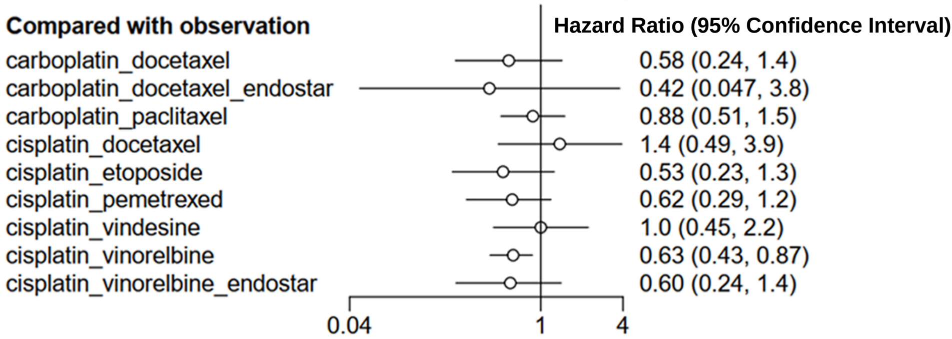

C

carboplatin_ docetaxel									
1.37 (0.19, 10.16)	carboplatin_ docetaxel_ endostar								
0.66 (0.23, 1.9)	0.48 (0.05, 4.53)	carboplatin_ paclitaxel							
0.42 (0.11, 1.68)	0.31 (0.03, 3.44)	0.63 (0.26, 1.57)	cisplatin_ docetaxel						
1.1 (0.32, 3.84)	0.8 (0.08, 8.14)	1.66 (0.6, 4.56)	2.62 (0.68, 10.11)	cisplatin_ etoposide					
0.94 (0.31, 3.14)	0.7 (0.07, 6.97)	1.42 (0.61, 3.71)	2.25 (0.67, 8.44)	0.86 (0.3, 2.77)	cisplatin_ pemetrexed				
0.58 (0.17, 1.95)	0.43 (0.04, 4.28)	0.88 (0.33, 2.29)	1.39 (0.37, 5.13)	0.53 (0.16, 1.72)	0.62 (0.2, 1.7)	cisplatin_ vindesine			
0.93 (0.36, 2.51)	0.68 (0.07, 6.23)	1.39 (0.75, 2.78)	2.21 (0.75, 6.84)	0.84 (0.34, 2.2)	0.98 (0.51, 1.78)	1.59 (0.68, 3.94)	cisplatin_ vinorelbine		
0.97 (0.28, 3.54)	0.72 (0.07, 7.46)	1.46 (0.54, 4.26)	2.32 (0.61, 9.36)	0.88 (0.26, 3.15)	1.03 (0.36, 2.78)	1.67 (0.52, 5.69)	1.05 (0.47, 2.37)	cisplatin_ vinorelbine_ endostar	
0.58 (0.24, 1.43)	0.42 (0.05, 3.74)	0.88 (0.51, 1.5)	1.39 (0.49, 3.94)	0.53 (0.22, 1.25)	0.62 (0.28, 1.19)	1 (0.45, 2.22)	0.63 (0.43, 0.87)	0.6 (0.24, 1.41)	observation

D

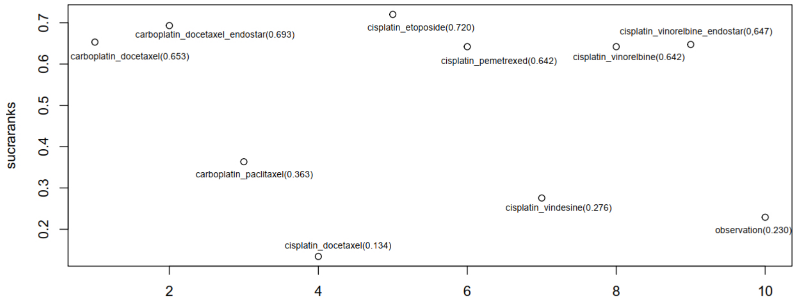

Figure 3.
A) Network evidence of the comparisons for the best adjuvant chemotherapy with regard to Relapse-free Survival (RFS).
B) Forest plots of the comparisons for the different cytotoxicity chemotherapy regimens with regard to RFS.
C) The league table of the comparisons for the different cytotoxicity chemotherapy regimens with regard to RFS.
D) Surface under the cumulative ranking curve (SUCRA) value of every cytotoxicity chemotherapy regimens with regard to RFS.

For peer review only - http://bmjopen.bmj.com/site/about/guidelines.xhtml

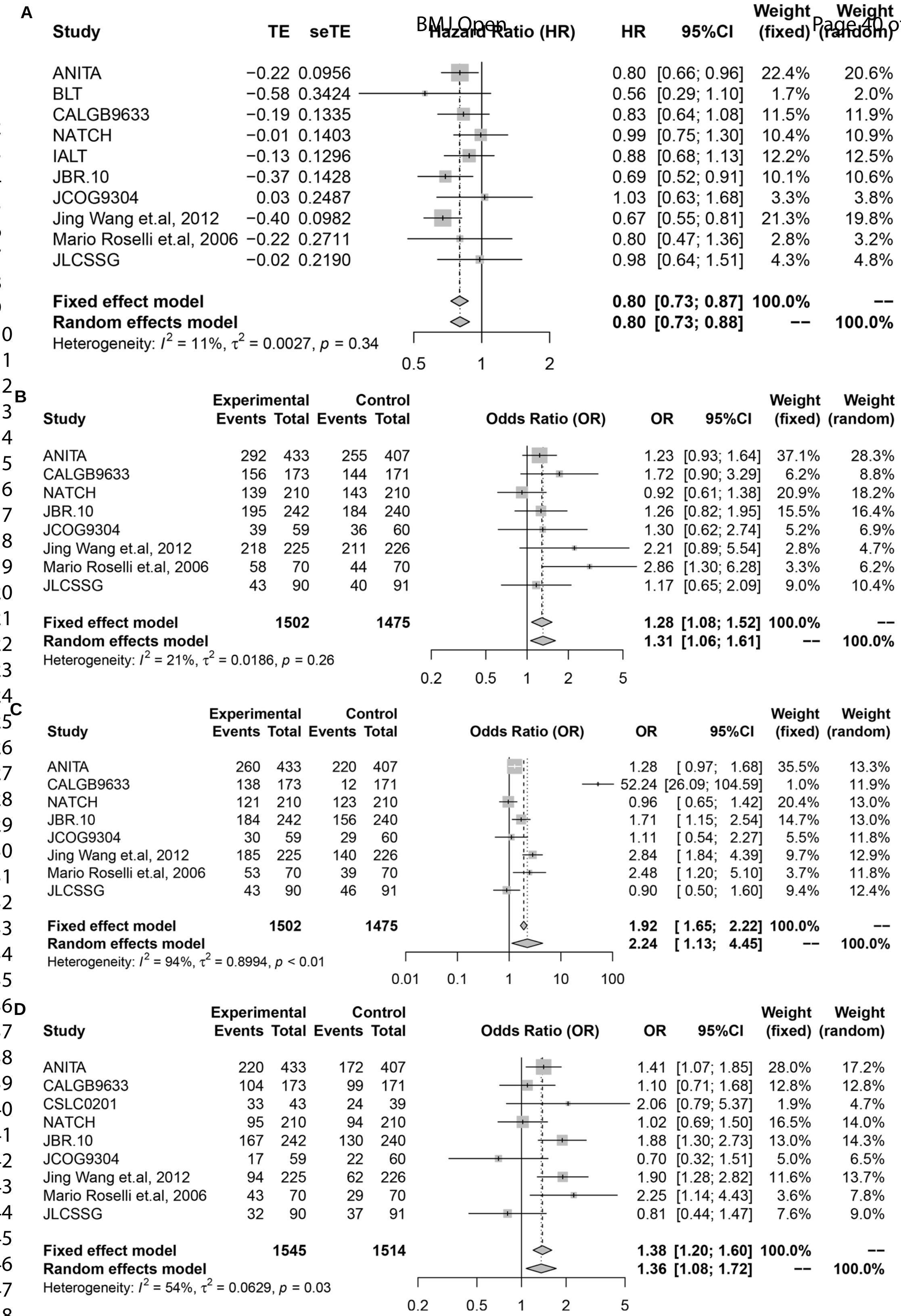

Figure 4.
A) The efficacy of platinum-based postoperative adjuvant chemotherapy in improving the Overall Survival (OS) compared with the observation group.
B) 2-year ; **C)** 3-year; **D)** 5-year OS rate of the chemotherapy arm compared to the observation arm.

For peer review only - http://bmjopen.bmj.com/site/about/guidelines.xhtml

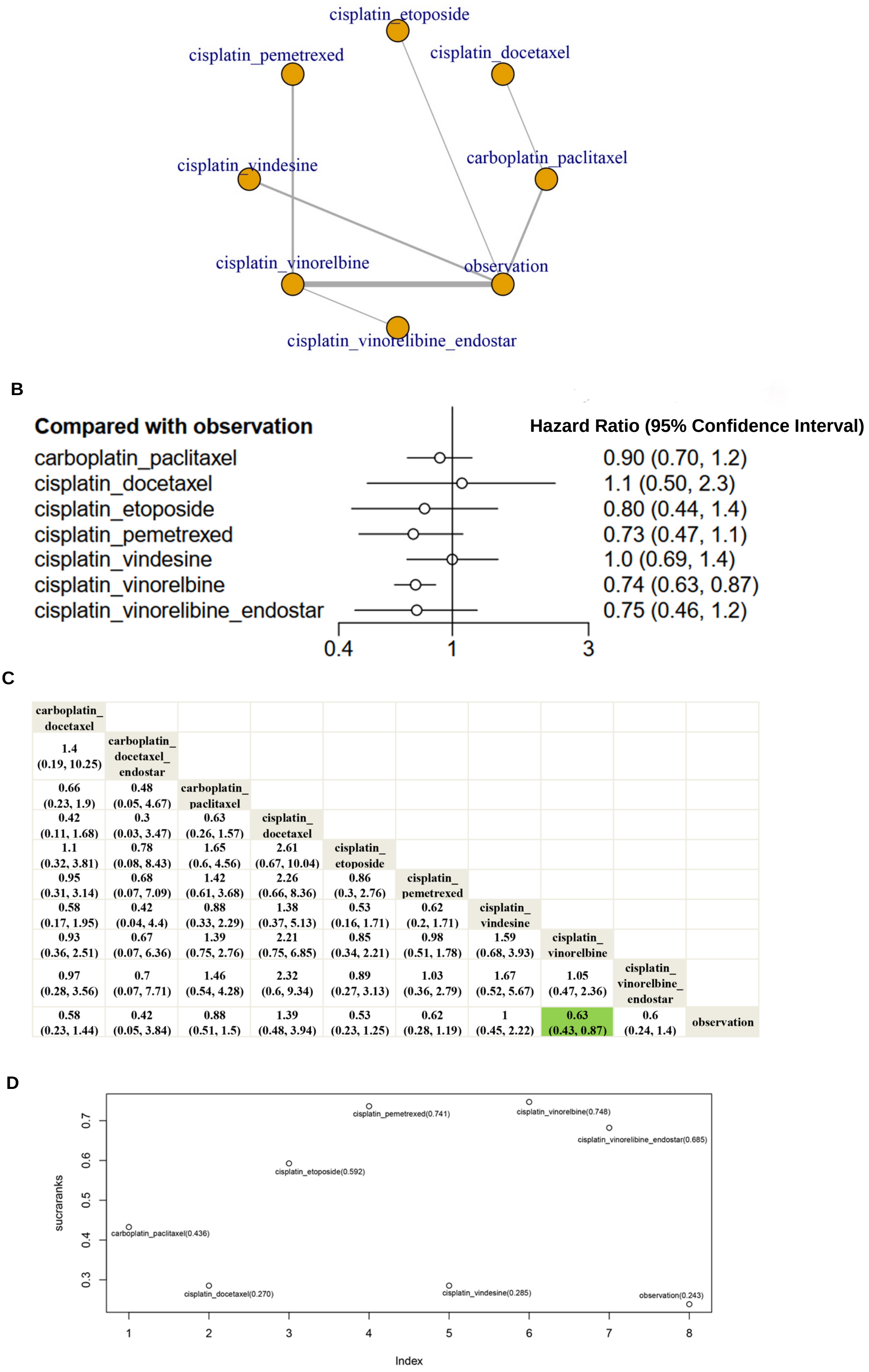

carboplatin_ docetaxel									
1.4 (0.19, 10.25)	carboplatin_ docetaxel_ endostar								
0.66 (0.23, 1.9)	0.48 (0.05, 4.67)	carboplatin_ paclitaxel							
0.42 (0.11, 1.68)	0.3 (0.03, 3.47)	0.63 (0.26, 1.57)	cisplatin_ docetaxel						
1.1 (0.32, 3.81)	0.78 (0.08, 8.43)	1.65 (0.6, 4.56)	2.61 (0.67, 10.04)	cisplatin_ etoposide					
0.95 (0.31, 3.14)	0.68 (0.07, 7.09)	1.42 (0.61, 3.68)	2.26 (0.66, 8.36)	0.86 (0.3, 2.76)	cisplatin_ pemetrexed				
0.58 (0.17, 1.95)	0.42 (0.04, 4.4)	0.88 (0.33, 2.29)	1.38 (0.37, 5.13)	0.53 (0.16, 1.71)	0.62 (0.2, 1.71)	cisplatin_ vindesine			
0.93 (0.36, 2.51)	0.67 (0.07, 6.36)	1.39 (0.75, 2.76)	2.21 (0.75, 6.85)	0.85 (0.34, 2.21)	0.98 (0.51, 1.78)	1.59 (0.68, 3.93)	cisplatin_ vinorelbine		
0.97 (0.28, 3.56)	0.7 (0.07, 7.71)	1.46 (0.54, 4.28)	2.32 (0.6, 9.34)	0.89 (0.27, 3.13)	1.03 (0.36, 2.79)	1.67 (0.52, 5.67)	1.05 (0.47, 2.36)	cisplatin_ vinorelbine_ endostar	
0.58 (0.23, 1.44)	0.42 (0.05, 3.84)	0.88 (0.51, 1.5)	1.39 (0.48, 3.94)	0.53 (0.23, 1.25)	0.62 (0.28, 1.19)	1 (0.45, 2.22)	0.63 (0.43, 0.87)	0.6 (0.24, 1.4)	observation

Figure 5.
A) Network evidence of the comparisons for the best adjuvant chemotherapy with regard to Overall Survival (OS).
B) Forest plots of the comparisons for the different cytotoxicity chemotherapy regimens with regard to OS.
C) The league table of the comparisons for the different cytotoxicity chemotherapy regimens with regard to OS.
D) Surface under the cumulative ranking curve (SUCRA) value of every cytotoxicity chemotherapy regimens with regard to OS.

For peer review only - http://bmjopen.bmj.com/site/about/guidelines.xhtml

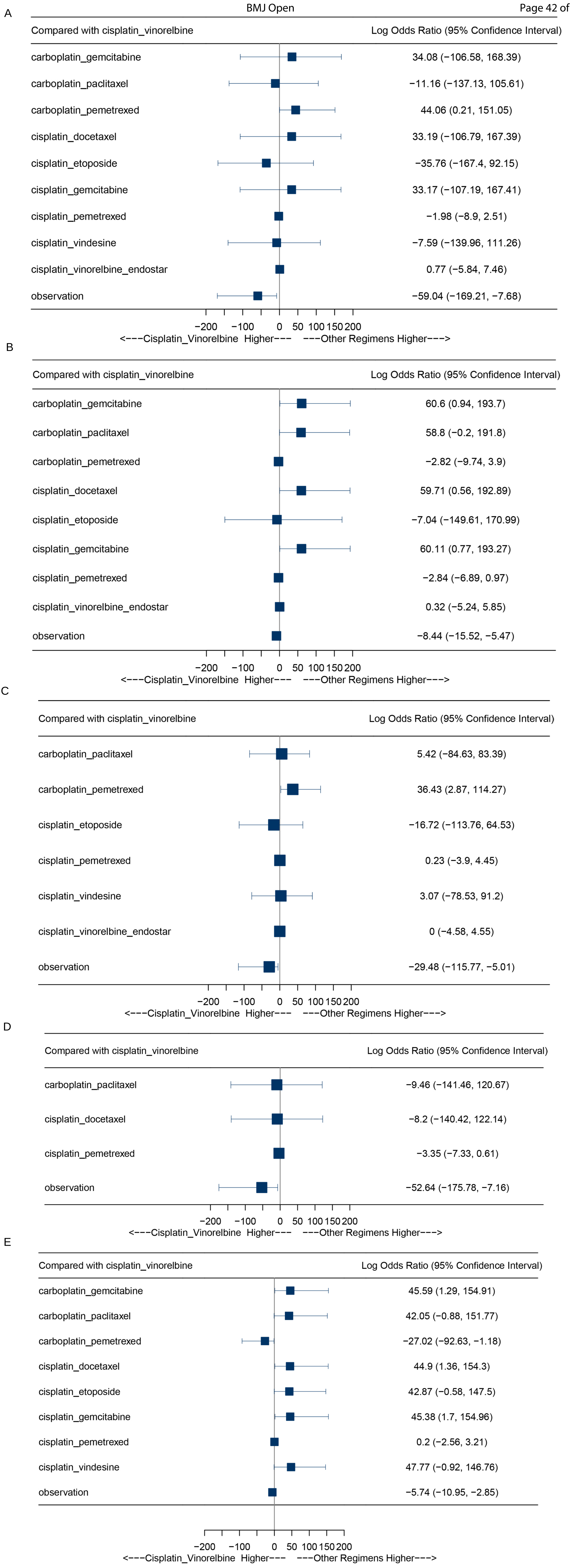

Figure 6. Forest plots of the comparisons for the different cytotoxicity chemotherapy regimens concerning the presence of **A)** anemia;**B)**neutropenia;**C)**thrombocytopenia;**D)**febrile neutropenia;**E)**nausea/vomiting.

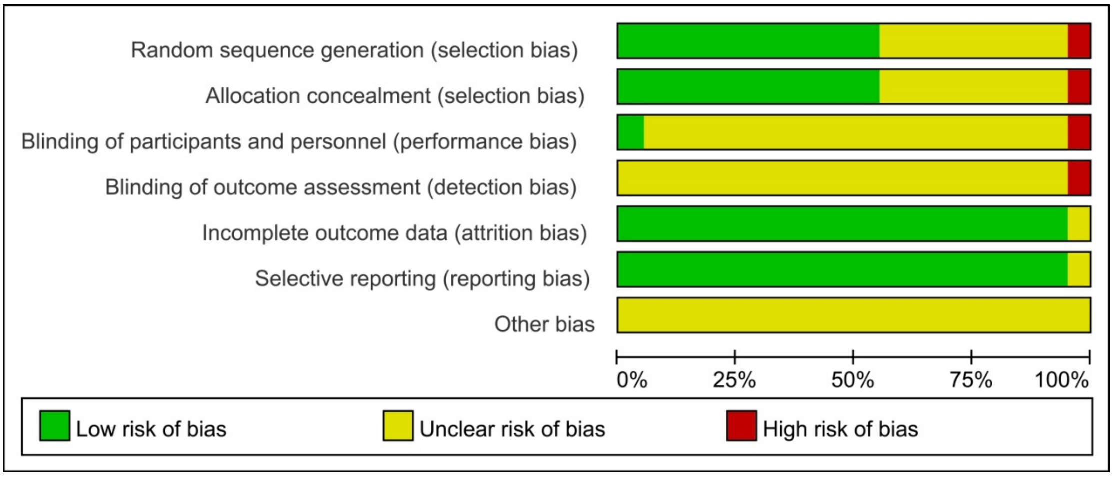

Supplementary Figure 1. A) Risk of bias graph.

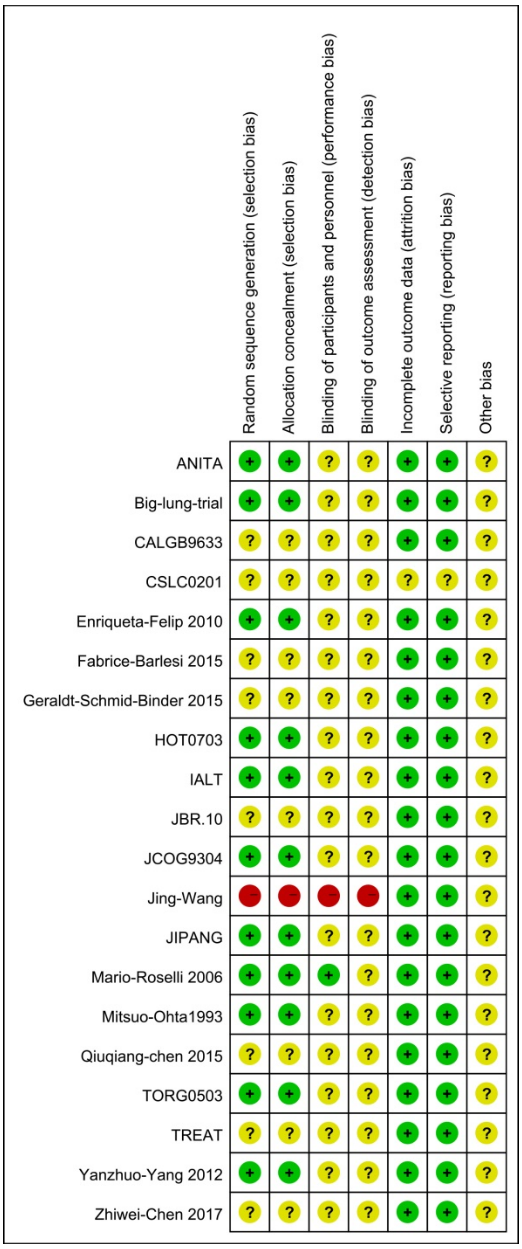

B) Risk of bias summary

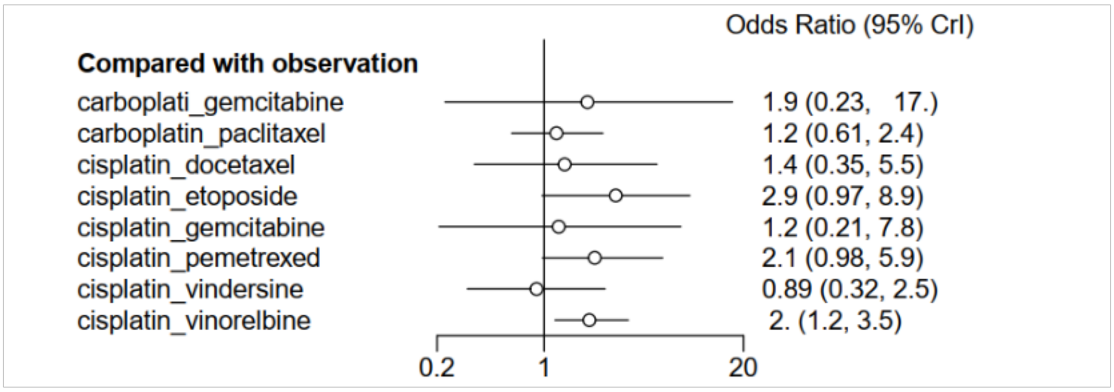

Supplementary.Figure 2.

A) Forest plots of the comparisons for the different cytotoxicity chemotherapy regimens with regard to 2-year RFS rate.

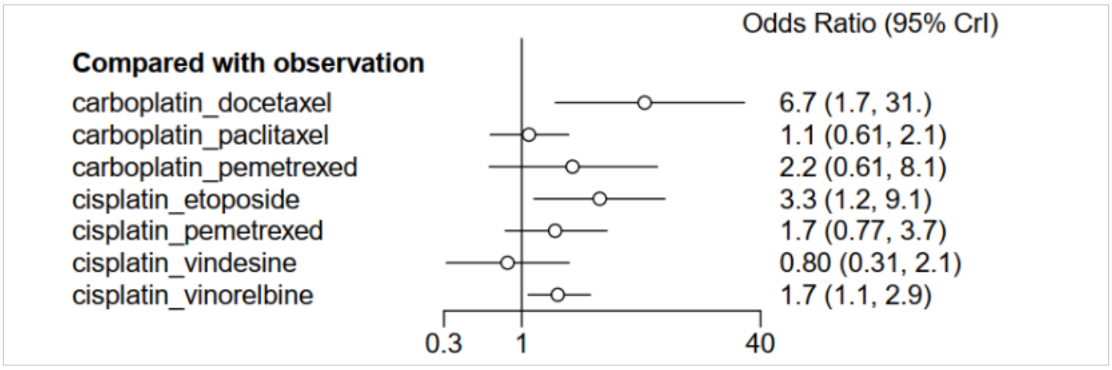

B) Forest plots of the comparisons for the different cytotoxicity chemotherapy regimens with regard to 3-year RFS rate.

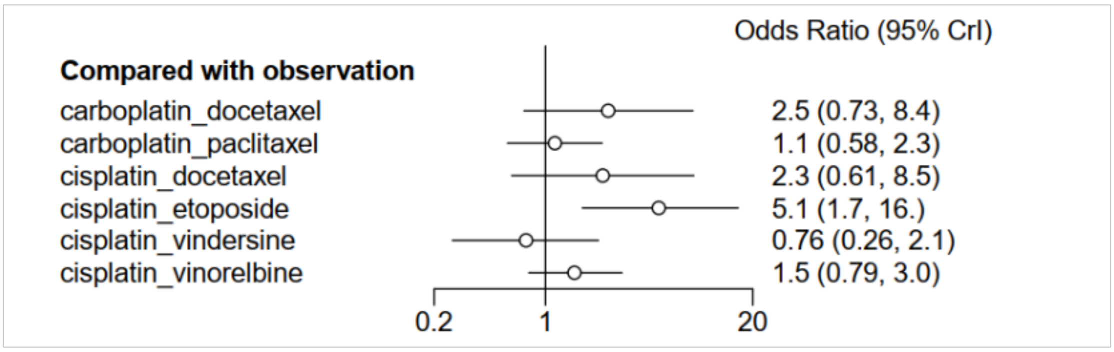

C) Forest plots of the comparisons for the different cytotoxicity chemotherapy regimens with regard to 5-year RFS rate.

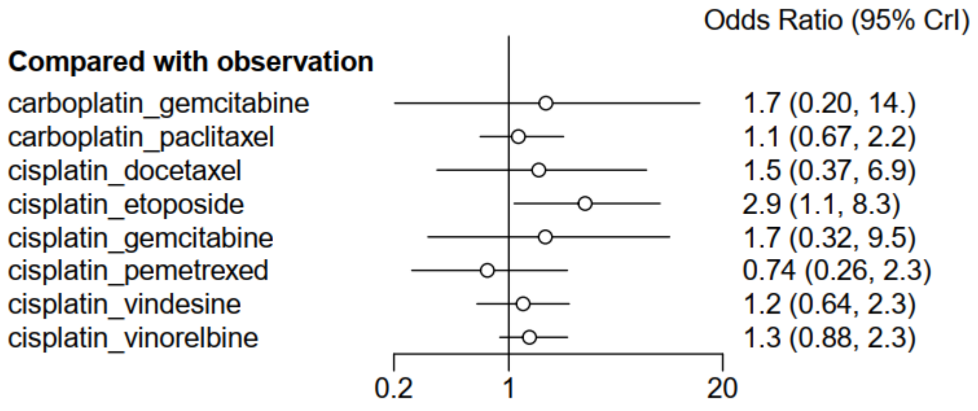

Supplementary.Figure 3.

A) Forest plots of the comparisons for the different cytotoxicity chemotherapy regimens with regard to 2-year OS rate.

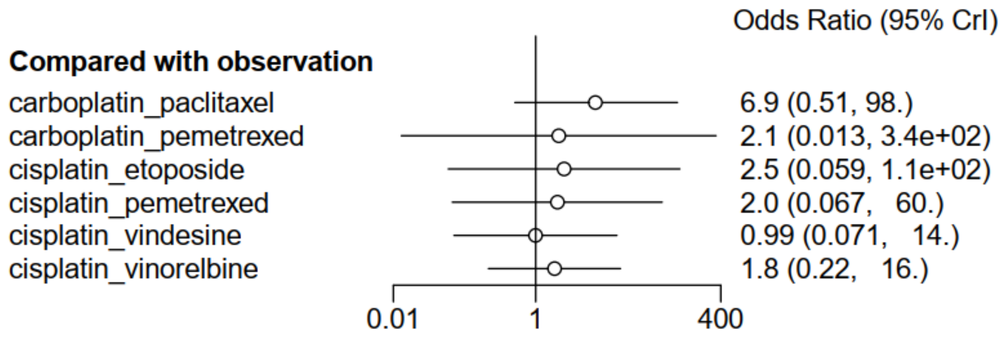

B) Forest plots of the comparisons for the different cytotoxicity chemotherapy regimens with regard to 3-year OS rate.

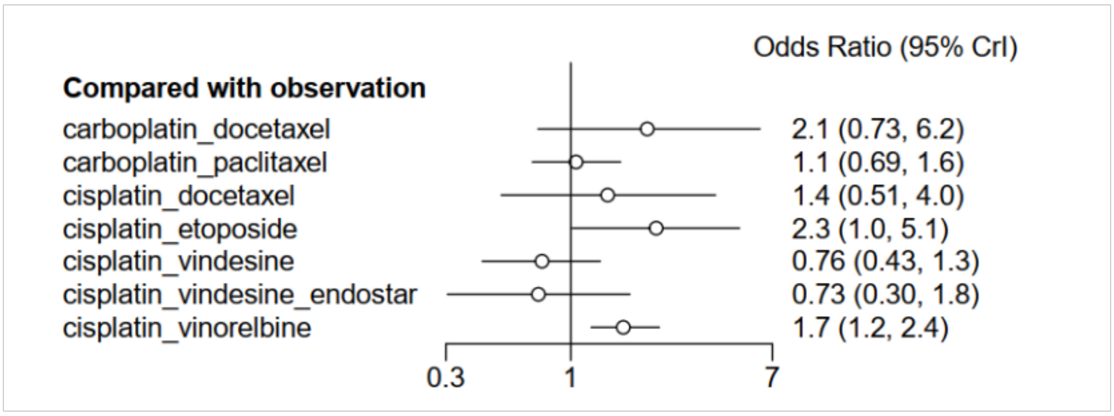

C) Forest plots of the comparisons for the different cytotoxicity chemotherapy regimens with regard to 5-year OS rate.

PRISMA 2009 Checklist

Section/topic	#	Checklist item	Reported on page #
**TITLE**			
Title	1	Identify the report as a systematic review, meta-analysis, or both.	Page 1
**ABSTRACT**			
Structured summary	2	Provide a structured summary including, as applicable: background; objectives; data sources; study eligibility criteria, participants, and interventions; study appraisal and synthesis methods; results; limitations; conclusions and implications of key findings; systematic review registration number.	Page 3-5
**INTRODUCTION**			
Rationale	3	Describe the rationale for the review in the context of what is already known.	Page 6-7
Objectives	4	Provide an explicit statement of questions being addressed with reference to participants, interventions, comparisons, outcomes, and study design (PICOS).	Page 7-8
**METHODS**			
Protocol and registration	5	Indicate if a review protocol exists, if and where it can be accessed (e.g., Web address), and, if available, provide registration information including registration number.	NA
Eligibility criteria	6	Specify study characteristics (e.g., PICOS, length of follow-up) and report characteristics (e.g., years considered, language, publication status) used as criteria for eligibility, giving rationale.	Page 9-10
Information sources	7	Describe all information sources (e.g., databases with dates of coverage, contact with study authors to identify additional studies) in the search and date last searched.	Page 9
Search	8	Present full electronic search strategy for at least one database, including any limits used, such that it could be repeated.	Page 9
Study selection	9	State the process for selecting studies (i.e., screening, eligibility, included in systematic review, and, if applicable, included in the meta-analysis).	Page 9-10
Data collection process	10	Describe method of data extraction from reports (e.g., piloted forms, independently, in duplicate) and any processes for obtaining and confirming data from investigators.	Page 10-11
Data items	11	List and define all variables for which data were sought (e.g., PICOS, funding sources) and any assumptions and simplifications made.	Page 10-11
Risk of bias in individual studies	12	Describe methods used for assessing risk of bias of individual studies (including specification of whether this was done at the study or outcome level), and how this information is to be used in any data synthesis.	Page11
Summary measures	13	State the principal summary measures (e.g., risk ratio, difference in means).	Page 11-12
Synthesis of results	14	Describe the methods of handling data and combining results of studies, if done, including measures of consistency (e.g., I^2) for each meta-analysis.	Page

For peer review only - http://bmjopen.bmj.com/site/about/guidelines.xhtml

PRISMA 2009 Checklist

			11-12

Section/topic	#	Checklist item	Reported on page #
Risk of bias across studies	15	Specify any assessment of risk of bias that may affect the cumulative evidence (e.g., publication bias, selective reporting within studies).	NA
Additional analyses	16	Describe methods of additional analyses (e.g., sensitivity or subgroup analyses, meta-regression), if done, indicating which were pre-specified.	NA
**RESULTS**			
Study selection	17	Give numbers of studies screened, assessed for eligibility, and included in the review, with reasons for exclusions at each stage, ideally with a flow diagram.	Page 13
Study characteristics	18	For each study, present characteristics for which data were extracted (e.g., study size, PICOS, follow-up period) and provide the citations.	Page 13-14
Risk of bias within studies	19	Present data on risk of bias of each study and, if available, any outcome level assessment (see item 12).	Page 14
Results of individual studies	20	For all outcomes considered (benefits or harms), present, for each study: (a) simple summary data for each intervention group (b) effect estimates and confidence intervals, ideally with a forest plot.	NA
Synthesis of results	21	Present results of each meta-analysis done, including confidence intervals and measures of consistency.	Page 14-20
Risk of bias across studies	22	Present results of any assessment of risk of bias across studies (see Item 15).	NA
Additional analysis	23	Give results of additional analyses, if done (e.g., sensitivity or subgroup analyses, meta-regression [see Item 16]).	NA
**DISCUSSION**			
Summary of evidence	24	Summarize the main findings including the strength of evidence for each main outcome; consider their relevance to key groups (e.g., healthcare providers, users, and policy makers).	Page 21-25
Limitations	25	Discuss limitations at study and outcome level (e.g., risk of bias), and at review-level (e.g., incomplete retrieval of identified research, reporting bias).	Page 25-26
Conclusions	26	Provide a general interpretation of the results in the context of other evidence, and implications for future research.	Page 26
**FUNDING**			
Funding	27	Describe sources of funding for the systematic review and other support (e.g., supply of data); role of funders for the systematic review.	Page 2

From: Moher D, Liberati A, Tetzlaff J, Altman DG, The PRISMA Group (2009). Preferred Reporting Items for Systematic Reviews and Meta-Analyses: The PRISMA Statement. PLoS Med 6(7): e1000097.

For peer review only - http://bmjopen.bmj.com/site/about/guidelines.xhtml

PRISMA 2009 Checklist

doi:10.1371/journal.pmed1000097

For more information, visit: www.prisma-statement.org.

For peer review only

For peer review only - http://bmjopen.bmj.com/site/about/guidelines.xhtml

Supplementary.Material.2 The detailed searching strategy

*PubMed:

(“Carcinoma, non-small cell lung” OR “Lung carcinoma, non-small-cell” OR “Carcinoma, non-small cell lung” OR “Non-small cell lung cancer”)

AND (“adjuvant chemotherapy” OR “chemotherapy, adjuvant”)

AND (“platinum” OR “cisplatin” OR “carboplatin” or “nedaplatin” or “oxaliplatin”)

*EMBASE, and The Cochrane Library, Web of Science and Scopus Google Scholar:

(“Carcinoma, non-small cell lung” OR “Lung carcinoma, non-small-cell” OR “Carcinoma, non-small cell lung” OR “Non-small cell lung cancer”)

AND (“postoperative adjuvant chemotherapy”)

AND (“platinum” OR “cisplatin” OR “carboplatin” or “nedaplatin” or “oxaliplatin”)

In addition, searching performed in the database of Web of Science and Scopus Google Scholar was constrained to titles and abstracts while no limitations were applied in the PubMed, EMBASE, and The Cochrane Library.

For peer review only

BMJ Open

Investigation of the Optimal Platinum-based Regimen in the Postoperative Adjuvant Chemotherapy Setting for Early-stage Resected Non-small Lung Cancer: A Bayesian Network Meta-analysis

Journal:	*BMJ Open*
Manuscript ID	bmjopen-2021-057098.R2
Article Type:	Original research
Date Submitted by the Author:	16-May-2022

Complete List of Authors:	pang, lanlan; Sun Yat-sen University Cancer Center, Department of Medical Oncology<br>Gan, Jiadi; Sun Yat-sen University Cancer Center, Department of Medical Oncology<br>Huang, Yi-Hua; Sun Yat-sen University Cancer Center, Department of Medical Oncology<br>Liao, Jun; Sun Yat-sen University Cancer Center, Department of Medical Oncology<br>Lv, Yi; Sun Yat-Sen University, Zhongshan School of Medicine<br>Ali, Wael; Sun Yat-sen University Cancer Center, Department of Medical Oncology<br>Zhang, L ; Sun Yat-sen University Cancer Center, Department of Medical Oncology<br>Fang, Wenfeng; Sun Yat-sen University Cancer Center, Department of Medical Oncology

**Primary Subject Heading**:	Oncology
Secondary Subject Heading:	Oncology
Keywords:	Adult oncology < ONCOLOGY, Respiratory tract tumours < ONCOLOGY, Thoracic surgery < SURGERY, CHEMOTHERAPY

SCHOLARONE™
Manuscripts

I, the Submitting Author has the right to grant and does grant on behalf of all authors of the Work (as defined in the below author licence), an exclusive licence and/or a non-exclusive licence for contributions from authors who are: i) UK Crown employees; ii) where BMJ has agreed a CC-BY licence shall apply, and/or iii) in accordance with the terms applicable for US Federal Government officers or employees acting as part of their official duties; on a worldwide, perpetual, irrevocable, royalty-free basis to BMJ Publishing Group Ltd ("BMJ") its licensees and where the relevant Journal is co-owned by BMJ to the co-owners of the Journal, to publish the Work in this journal and any other BMJ products and to exploit all rights, as set out in our licence.

The Submitting Author accepts and understands that any supply made under these terms is made by BMJ to the Submitting Author unless you are acting as an employee on behalf of your employer or a postgraduate student of an affiliated institution which is paying any applicable article publishing charge ("APC") for Open Access articles. Where the Submitting Author wishes to make the Work available on an Open Access basis (and intends to pay the relevant APC), the terms of reuse of such Open Access shall be governed by a Creative Commons licence – details of these licences and which Creative Commons licence will apply to this Work are set out in our licence referred to above.

Other than as permitted in any relevant BMJ Author's Self Archiving Policies, I confirm this Work has not been accepted for publication elsewhere, is not being considered for publication elsewhere and does not duplicate material already published. I confirm all authors consent to publication of this Work and authorise the granting of this licence.

For peer review only

Investigation of the Optimal Platinum-based Regimen in the Postoperative Adjuvant Chemotherapy Setting for Early-stage Resected Non-small Lung Cancer: A Bayesian Network Meta-analysis

Running title: Postoperative Platinum-based Adjuvant Chemotherapy Setting for NSCLC：a Meta-analysis

Author:

Lan-Lan Pang,MD[1], Jia-Di Gan,MD[1], Yi-Hua Huang,MD[1], Jun Liao,MD[1], Yi Lv, MD[2], Wael-Abdullah -Sultan Ali, MD[1], Li Zhang, MD , Phd[1], Wen-Feng Fang, MD ,Phd[1#]

Institutions:

[1]Department of Medical Oncology, State Key Laboratory of Oncology in South China, Collaborative Innovation Center for Cancer Medicine, Sun Yat-sen University Cancer Center, Guangzhou 510060, People's Republic of China

[2] Zhongshan School of Medicine, Sun Yat-sen University, 74 Zhongshan II Road, Guangzhou510080, People's Republic of China.

#Correspondence:

Wenfeng Fang, MD, Phd, Department of Medical Oncology, State Key Laboratory of Oncology in South China, Collaborative Innovation Center for Cancer Medicine, Sun Yat-sen University Cancer Center, Guangzhou 510060, Peoples Republic of China. Email: fangwf@sysucc.org.cn.

Acknowledgments:

For peer review only

This work was financially supported by the Chinese National Natural Science Foundation Project (81772476). We thank all the co-authors for their contributions to this study.

Formatting of funding sources:

This work was supported by the Chinese National Natural Science Foundation Project (81772476).

For peer review only

Abstracts

Objective: This present study aimed to compare the efficacy and safety of different platinum adjuvant chemotherapy regimens for early-stage resected non-small-cell lung cancer (NSCLC).

Design: Systematic review with network meta-analysis (NMA) of randomized trials.

Data sources: PubMed, EMBASE, The Cochrane Library, Web of Science, and Scopus Google Scholar were searched through March 12th, 2021.

Eligibility criteria: Eligible randomized controlled trials comparing the postoperative platinum chemotherapy regimen with the observation-controlled group or comparing two platinum chemotherapy regimens head-to-head were included.

Data extraction and synthesis: The primary outcome was the efficacy of adjuvant chemotherapy regimens including relapse-free survival (RFS), overall survival (OS), 2, 3, 5-year RFS rate, and OS rate. The secondary outcome was the rate of grade3-4 toxicity assessments. Cochrane Handbook (Version 5) was utilized for the risk of bias assessment. Analyses were performed utilizing R-software version 4.3.1.

Results: 20 RCTs with a sample size of 5483 were enrolled in meta-analysis. The chemotherapy group had a significant RFS and OS advantage compared to the observation group [hazard ratio (HR), 0.67; 95% confidence interval (CI), 0.56 to 0.81, P<0.0001; HR, 0.80; 95% CI, 0.73 to 0.88, P<0.0001, respectively]. Compared with the observation arm, only the “Cisplatin_Vinorelbine” regimen had a significant RFS and OS advantage (HR, 0.63; 95%CI, 0.43 to 0.87; HR, 0.74; 95%CI, 0.63 to 0.87, respectively) while the remaining chemotherapy regimens had no significant difference

of efficacy compared to the observation group. In terms of the safety of adjuvant chemotherapy, the incidence of hematological toxicities and nausea/vomiting was not significantly higher in the "Cisplatin_Vinorelbine" arm than in other chemotherapy group.

Conclusion: The current study summarized the adjuvant cytotoxicity chemotherapy regimens for patients with early-stage resected NSCLC. Our analysis may provide some guiding significance for the clinicians when determining the optimal chemotherapy regimen.

Keywords: Non-small cell lung cancer, adjuvant chemotherapy, platinum-based, Network Meta-analysis

Trial registration: None

Strengths and limitations of this study

*The Bayesian Network Meta-analysis provided access to directly compare the efficacy and safety of different adjuvant chemotherapy regimens for NSCLC patients.

* It is difficult to determine the optimal subgroup populations who may obtain the benefit from certain cytotoxicity chemotherapy regimens.

* We did not incorporate any targeted or immune-biological therapy into the analysis due to the open-loop of interventions.

* The present study could not present a subgroup analysis stratified by postoperative radiotherapy or not as it was undertaken according to every center's policy.

For peer review only

Introduction:

Lung cancer is the most common cause of cancer-related deaths worldwide and patients with non-small lung cancer (NSCLC) account for almost 85% of all cases[1, 2]. Complete surgical resection is recognized as the standard treatment for patients with early-stage NSCLC. However, 30% to 70% of patients ultimately experience local or distant relapse and the five-year survival rate ranges from only 30% to 60%[3, 4]. The presence of micro-metastases, which could not be detected by conventional diagnostic techniques, increases the rate of relapse and exerts a negative impact on the survival of patients[5]. Therefore, adjuvant postoperative systematic therapy is necessary for patients experiencing surgical resection.

A pooled analysis conducted by the Lung Adjuvant Cisplatin Evaluation (LACE) Collaborative Group suggested that cisplatin-based adjuvant chemotherapy yield an absolute OS benefit of 5.40% at five years[6]. In light of this promising data, the American Society of Clinical Oncology (ASCO) guidelines recommended the cisplatin-based adjuvant postoperative chemotherapy regimen as the standard treatment for early-stage NSCLC patients[7]. However, the optimal adjuvant chemotherapy regimen has not yet been determined. “Cisplatin_Vinorelbine” has shown its promising efficacy compared with the observation group and is recognized as the standard adjuvant chemotherapy regimen currently[8, 9]. In addition, several RCTs have also investigated the feasibility, efficacy, and safety of cisplatin plus non-vinorelbine third-generation drug as the postoperative adjuvant chemotherapy [10-12]. Among them, “Cisplatin_Pemetrexed” is characterized as having promising efficacy and an

For peer review only

acceptable safety profile in comparison with "Cisplatin_vinorelbine"[12, 13]. Thus, despite the lack of level 1 data concerning the efficacy of cisplatin plus vinorelbine, it was still recommended by the National Comprehensive Cancer Network (NCCN) guidelines as the optional postoperative adjuvant chemotherapy regimen for non-squamous NSCLC[14]. Meanwhile, although carboplatin-based adjuvant regimens have not yet been recommended by guidelines, it might be a favorable choice for patients with comorbidities and unsuited to receive cisplatin when accompanied by the late toxicity [15, 16].

Recent advances in individualized treatment based on molecular and biological profiling have shaped the future of postoperative adjuvant chemotherapy. Substantial numbers of patients benefit from the corresponding adjuvant regimen. The patients unsuited to adjuvant cytotoxicity chemotherapy may benefit from the first-generation Tyrosine Kinase Inhibitor (TKI) but serval clinical trials showed poor outcomes of TKI adjuvant therapy until the advent of ADJUVANT study [17-19]. However, individualization could not apply to all NSCLC patients and those patients inapplicable to it have no choice but receive platinum adjuvant chemotherapy.

Postoperative adjuvant chemotherapy setting for early-stage resected NSCLC is of great significance for the oncological community in routine clinical practice. However, it seems difficult to conduct large RCT to figure out the preferred platinum chemotherapy regimen and utilizing the published data might be an alternative option. Hence, we conducted this systematic review and network meta-analysis (NMA) aiming

For peer review only

to compare the efficacy and safety of different platinum adjuvant chemotherapy regimens.

For peer review only

Methods:

This systematic review and NMA was performed in accordance with the Preferred Reporting Items for Systematic reviews and Meta-analyses guidelines for RCTs (The PRISMA Statement) (**Supplementary material 1**).

Data Sources

Two authors (L.L.P and J.D.G) independently searched the records in the electronic database of PubMed, EMBASE, and The Cochrane Library, Web of Science and Scopus Google Scholar. The searching terminal date was March 12th, 2021. Searching terms were focused on NSCLC, adjuvant chemotherapy, and platinum. If necessary, an additional manual search of related literature in the reference list would be carried out to enroll any relevant publications. The datasets utilized in this analysis could be obtained from the corresponding author upon request. Records were imported into EndnoteX9 software to eliminate duplications. The detailed strategy was presented in **Supplementary material 2**.

Trial Selection Criteria and Trial Identification

Two authors (L.L.P and Y.H.H) independently reviewed the title, abstracts, and keywords of identified citations to select appropriate articles for a full review. Any disagreement was resolved by consensus or through the judgement of a senior author (W.F.F). Trials would be eligible only if meeting all the following criteria: 1) patients with completely resected NSCLC (squamous and non-squamous) at stage IB–IIIA; 2) Eligible RCTs comparing the postoperative platinum chemotherapy regimen with observation-controlled group or those concerning two platinum chemotherapy

For peer review only

regimens head-to-head comparison; given that Vinorelbine, Etoposide, Pemetrexed, Docetaxel, Paclitaxel, Gemcitabine, Vindesine are currently commonly used in the routine clinical practice, the counterpart of the platinum doublet including these above-mentioned drugs were considered eligible; and a platinum triplet must be a platinum doublet combined with anti-angiogenesis drug; 3) full-text publications or conference abstract; 4) non-language restrictions. Publications would be disregarded if meeting any of the following criteria: 1) any single perioperative chemotherapy, neo-adjuvant chemotherapy, maintenance chemotherapy, or radio-chemotherapy; 2) chemo-naive patients with advanced, incurable, or recurrent NSCLC; 3) no RCTs including reviews, case series or retrospective trials.

Outcomes and Data Extraction

Two authors (L.L.P and J.L) independently performed data extraction and any discrepancies were eliminated by consensus. Data for eligible trials related to basic characteristics were extracted, including: trial/author name, publication year, country of origin/multi-centers, sample size per group, chemotherapy regime, administration dose of each treatment and number of cycles, follow-up time, phases II/III, basic patients' characteristics (age, sex ratio, stage, pathology, performance status).

The primary outcome was the efficacy of adjuvant chemotherapy regimens including RFS, OS, 2, 3, 5-year RFS rate, and OS rate. RFS was defined as the interval from day of randomized assignment to disease recurrence or death, whichever occurred first, while OS referred to the time from randomization to death from any cause.

The secondary outcomes were the rate of grade3-4 toxicity assessment, including

For peer review only

hematological (anemia, neutropenia, febrile neutropenia, and thrombocytopenia) and non-hematological (nausea/vomiting) adverse events. No additional information was requested from the authors. Also, Parmar's method would be utilized to obtain survival if necessary.

Quality and Risk of Bias Assessment

Two researchers (L.L.P and J.D.G) independently assessed the risk of bias of enrolled trials according to the recommendations of the Cochrane Handbook of Systematic Reviews of Interventions (Version 5, http://handbook.cochrane.org)[20]. Disagreements were resolved by discussion.

Data Synthesis and Analysis

For RFS and OS, the logarithm of hazard ratio (HR) and their standard error (SE) were pooled into analysis through a Bayesian multiple treatment network meta-analysis with random effects. As for the dichotomous variables, odds ratio (OR) with 95% confidence interval (CI) was applied to calculate. When a network diagram indicated two or more independent loops, only the loop containing "Cisplatin_Vinorelbine" was selected for further analysis.

Random effects and consistency model was computed utilizing Markov chain Monte Carlo methods with Gibbs sampling based on simulations of 50000 iterations and 20000 adaptions in each of 4 chains. For a forest plot, "observation" was chosen as the common reference comparator in the analysis of RFS and OS while "Cisplatin_Vinorelbine" was applied in the analysis of chemotherapy toxicity. A league table for the survival analysis was presented with the logarithm of HR and their 95%

For peer review only

CI. The surface under the cumulative ranking curve (SUCRA), represents the percentage of efficacy achieved by an agent compared to an imaginary agent that is always the best without uncertainty (ie, SUCRA = 100%)[21]. Namely, the SUCRA value would be 1 if treatment is certain to be the best and 0 if it is certain to be the worst. Higher SUCRA scores correspond to a higher ranking for extending survival.

All analyses in this article were performed utilizing R-software version 4.3.1 and the gemtc package version 0.8 that interfaces with JAGS version 4.3.0 for computing a Markov chain.

Patient and public involvement

Patients and the public were not involved in the design or planning of the study.

For peer review only

Results

The search process, study characteristics, and quality assessment

A total of 3432 records yielded after searching the database and 1991 records were left to be identified after removing 1692 duplications. Then, 1905 irrelevant citations were eliminated by skimming their title, abstracts, and keywords, leaving 86 articles to be considered potentially eligible. 69 articles were further excluded for irrelevant to topic (n=45); non-RCTs (n=11); non-accessible outcome (n=9); non-accessible text (n=4); and 17 articles were enrolled into analysis after skimming their full-text. Additionally, three studies were included by browsing the references. Finally, 20 studies with a sample size of 5483 were enrolled into meta-analysis **(Figure 1)**.

Table 1 showed the basic characteristics of enrolled studies and participants. These studies were conducted in countries worldwide, most of which were Japan[22-26] and China[11, 27-29], followed by France[30, 31], Germany[32], Italy[33], USA[34], UK[35], and North Carolina[10]. Noteworthy, three of them were conducted in multi-countries[36-38]. 11 studies compared the postoperative platinum chemotherapy regimen with the observation-controlled group while the remaining nine studies comparing two platinum chemotherapy regimens head-to-head. Chemotherapy regimens included “Cisplatin_Vinorelbine”, “Cisplatin_Pemetrexed”, “Cisplatin_Gemcitabine”, “Cisplatin_Vindesine”, “Cisplatin_Etoposide”, “Cisplatin _Docetaxel”, “Carboplatin_Gemcitabine”, “Carboplatin_Pemetrexed”, “Carboplatin_ Paclitaxel”, “Carboplatin_Docetaxel_Endostar”, “Carboplatin_Docetaxel”, “Cisplatin _Vinorelbine_Endostar”. The median follow-up time ranged from 20.2 to 116 months.

For peer review only

Of note, we have noticed four well-designed RCTs during the process of eligible trial selection[39-42]. Nevertheless, we could not obtain the specific outcomes in the subgroup populations per treatment regimens from these clinical trials, whereas only the outcomes concerning the comparison between the observation group and "Cisplatin _Vinorelbine" were available in BLT or IALT trial[31, 35]. Given that the objective of the present work was to investigate the optimal platinum-based regimen in the postoperative adjuvant chemotherapy setting for early-stage resected NSCLC, we only enrolled the BLT and IALT while leaving the remaining four RCTs out.

Based on the Cochrane Risk of Bias evaluation, one of the enrolled studies had a high risk of selection bias due to its quasi-randomized setting while nearly half the studies did not clarify the randomization methods in detail. One study had a high risk of performance bias while nearly all of the studies did not mention utilizing the blind design or not. Attrition and reporting bias did not exist in the enrolled studies except one whose bias could not be evaluated due to a conference abstract (**Supplementary figure 1A-B**).

Primary outcome---RFS

10 studies were enrolled to analyze the RFS of platinum postoperative adjuvant chemotherapy compared with the observation group. We found that the chemotherapy group had a significant RFS advantage compared versus the observation group (HR, 0.67; 95%CI, 0.56 to 0.81; I^2=64%, P<0.0001) **(Figure 2A)**. Moreover, in terms of 2-year RFS rate, 3-year RFS rate and 5-year RFS rate, the chemotherapy arm presented higher RFS rate in comparison with the observation arm (OR=1.62, 95%CI 1.20 to 2.18,

For peer review only

I^2=68%, P=0.0016; OR=1.50, 95%CI 1.11 to 2.03, I^2=72%, P=0.0081; OR=1.49, 95%CI 1.09 to 2.04, I^2=69%, P=0.0132; respectively)**(Figure 2B-D)**.

15 clinical trials were enrolled to investigate the RFS of several chemotherapy regimens for patients with early-stage resected NSCLC. In the Network evidence figure, each node represents a type of treatment. Solid lines connect treatments that are directly compared in at least one study. The thickness of connections varies based on the number of studies involved in a comparison. Network evidence of the comparisons for the best adjuvant chemotherapy concerning the RFS was shown in **Figure 3A**. Almost all platinum chemotherapy has a direct comparison with "Observation" except "Cisplatin_Docetaxel", "Cisplatin_Pemetrexed", "Cisplatin_ Vinorelbine_Endostar" and "Carboplatin_Docetaxel_Endostar". Furthermore, the number of trials that compared "Cisplatin_Vinorelbine" versus "Observation" ranked the first. Furthermore, compared with the observation arm, only the "Cisplatin_Vinorelbine" regimen had a significant RFS advantage (HR, 0.63; 95%CI, 0.43 to 0.87) while there was no significant difference between the remaining chemotherapy regimens and the observation group---"Carboplatin_Docetaxel" (HR, 0.58; 95%CI, 0.24 to 1.4); "Carboplatin_Docetaxel_Endostar" (HR, 0.42; 95%CI, 0.05 to 3.8); "Carboplatin_ Paclitaxel" (HR, 0.88; 95%CI, 0.51 to 1.50); "Cisplatin_Docetaxel" (HR, 1.40; 95%CI, 0.49 to 3.90); "Cisplatin_Etoposide" (HR, 0.53; 95%CI, 0.23 to 1.30); " Carboplatin_Pemetrexed" (HR, 0.62; 95%CI, 0.29 to 1.20); "Cisplatin_Vindesine" (HR, 1.00; 95%CI, 0.45 to 2.20); "Cisplatin_Vinorelbine_Endostar" (HR, 0.60; 95%CI, 0.24 to 1.40) (**Figure 3B**). In addition, according to the league table, no significant

For peer review only

For peer review only - http://bmjopen.bmj.com/site/about/guidelines.xhtml

difference was observed in the remaining comparisons, eg “Cisplatin_ Vinorelbine” and “Cisplatin_Pemetrexmed”. (**Figure 3C**). In the rank of network meta-analysis, the biggest SUCRA value holds the potential to be the best chemotherapy regime in extending RFS. Our results showed that “Cisplatin_Etoposide” (72.0%) was the most effective chemotherapy regime to improve RFS, followed by “Carboplatin_Docetaxel_Endostar” (69.30%), “Carboplatin_Docetaxel” (65.31%), “Cisplatin_Vinorelbine” (64.73%), “Cisplatin_ Pemetrexed” (64.2%), “Cisplatin_Vindesine” (64.2%), “Carboplatin_Paclitaxel” (36.3%), “Carboplatin_Vindesine” (27.6%), “Cisplatin_Vinorelbine_Endostar” (23.0%) and “Cisplatin_Docetaxel” (13.4%) (**Figure 3D**). In terms of the 2-year RFS rate, 3-year RFS rate and 5-year RFS rate, “Cisplatin_Vinorelbine” was an effective therapeutic method in improving 2-year RFS rate (OR=2.00, 95%CI 1.20 to 3.50); “Carboplatin_Docetaxel” (OR=6.70, 95%CI 1.70 to 31.00) “Cisplatin_Etoposide” (OR=3.30, 95%CI 1.20 to 9.10) and “Cisplatin_Vinorelbine” (OR=1.70, 95%CI 1.10 to 2.90) had a significantly higher 3-year RFS rate than observation arm; wherase “Cisplatin_Etoposide” (OR=5.10, 95%CI 1.70 to 16.00) was an effective method in improving 5-year RFS rate. However, there was no significant difference between the remaining chemotherapy regimens and observation arm concerning the RFS rate (**Supplementary figure 2A-C**).

Primary outcome--- OS

To compare the efficacy of platinum chemotherapy arm with the observation arm in extending OS, we enrolled 10 studies into analysis. Our results showed that the OS in

For peer review only

280 the chemotherapy group was significantly longer than the observation group (HR, 0.80; 281 95%CI, 0.73 to 0.88; I^2=11%, P<0.0001) (**Figure 4A**). As for 2-year OS rate, 3-year 282 OS rate and 5-year OS rate, the chemotherapy group showed a significant survival 283 advantage than the observation group (OR=1.31, 95%CI 1.06 to 1.61, I^2=21%, 284 P=0.0117; OR=2.24, 95% CI 1.13 to 4.45, I^2=94%, P=0.0081; OR=1.36, 95% CI 1.08 285 to 1.72, I^2=54%, P=0.0100; respectively)(**Figure 4B-D**).

286 Moreover, 14 clinical trials with eight treatment regimens were enrolled to investigate 287 the optimum adjuvant chemotherapy regimens in extending OS. Network evidence of 288 these chemotherapy regimens was shown in **Figure 5A**. Almost all platinum 289 chemotherapy has a direct comparison with "Observation" except 290 "Cisplatin_Docetaxel", "Cisplatin_Pemetrexed", and "Cisplatin_ 291 Vinorelbine_Endostar". Similar to RFS, the number of trials related to 292 "Cisplatin_Vinorelbine" comparing versus "Observation" also ranked the first in 293 extending OS. Compared with the observation arm, only the "Cisplatin_Vinorelbine" 294 regimen had a significant OS advantage(HR, 0.74; 95%CI, 0.63 to 0.87) while there 295 was no significant difference between the remaining chemotherapy regimens and the 296 observation group---"Carboplatin_Paclitaxel" (HR,0.90; 95%CI, 0.70 to 1.20); 297 "Cisplatin_Docetaxel" (HR, 1.10; 95%CI, 0.50 to 2.30) ; "Cisplatin_Etoposide" (HR, 298 0.80; 95% CI, 0.44 to 1.40); "Cisplatin_ Pemetrexed" (HR, 0.73; 95%CI, 0.47 to 1.10) 299 ; " Cisplatin_Vindesine" (HR, 1.00; 95%CI, 0.69 to 1.40); 300 "Cisplatin_Vinorelbine_Endostar" (HR, 0.75; 95%CI, 0.46 to 1.20) (**Figure 5B**). 301 Similar to RFS, as the league table showed, there was no significant difference among

For peer review only

these remaining paltimum chemotherapy regimens in improving OS for patients with early-stage resected NSCLC (**Figure 5C**). In terms of the rank of network meta-analysis, we found that "Cisplatin_Vinorelbine" (74.8%) was the most effective therapeutic arm for the improvement of OS, followed by "Cisplatin_Pemetrexed" (74.1%), "Cisplatin_Vinorelbine_Endostar" (68.5%), "Cisplatin_Etoposide" (59.2%), "Carboplatin_Paclitaxel" (43.6%), "Cisplatin_ Vindesine" (28.5%), "Cisplatin_Docetaxel" (27.0%) and "observation" (24.3%) (**Figure 5D**). In addition, "Cisplatin_Etoposide" was an effective chemotherapy regimen to improve the 2-year OS rate (OR=2.90, 95% CI 1.10 to 8.30) and 5-year OS rate (OR= 6.70, 95% CI 1.70 to 3.10) while "Cisplatin_Vinorelbine" had a significantly higher 5-year OS rate than observation arm (OR=1.70, 95% CI 1.10 to 2.90). However, there was no significant difference between the remain chemotherapy regimens and observation arm concerning 2-year OS rate, 3-year OS rate and 5-year OS rate (**Supplementary figure 3A-C**).

Secondary outcome---anemia, neutropenia, thrombocytopenia, febrile neutropenia and nausea/vomiting

15 clinical trials were enrolled to compare the presence of anemia among different adjuvant chemotherapy regimens. We found that the incidence of anemia in the "Carboplatin_Pemetrexed" arm was significantly higher than the " Cisplatin_Vinorelbine" arm (Log OR=44.06, 95% CI 0.21 to 151.05). However, there was no significant difference between the remaining chemotherapy regimens and "Cisplatin_Vinorelbine" (**Figure 6A**).

As for neutropenia, we enrolled 13 clinical trials to compare the safety of different

For peer review only

324 adjuvant chemotherapy regimens. The neutropenia was more frequently observed in patients receiving "Carboplatin_Gemcitabine" (Log OR= 60.60, 95%CI 0.94 to 193.7)， "Cisplatin_Gemcitabine" (Log OR= 60.11, 95%CI 0.77 to 193.27)， and "Cisplatin_Docetaxel" (Log OR= 59.71, 95%CI 0.56 to 192.89) than Cisplatin_Vinorelbine". However, the incidence of neutropenia in the "Cisplatin_Vinorelbine" group was not significantly higher than the remaining chemotherapy regimens group (**Figure 6B**).

Also, as for thrombocytopenia, we enrolled 11 clinical trials to investigate the safety of different adjuvant chemotherapy regimens. Patients medicated with "Carboplatin_Pemetrexed" were significantly observed thrombocytopenia compared with "Cisplatin_Vinorelbine" (Log OR=36.43, 95%CI 2.87 to 114.27). However, there was no significant difference between the remaining chemotherapy regimens and "Cisplatin_Vinorelbine" (**Figure 6C**).

In terms of febrile neutropenia, six clinical trials were included. Our results showed that there was no significant difference between these chemotherapy regimens and "Cisplatin_Vinorelbine" (**Figure 6D**).

In addition, 14 clinical trials were enrolled to analyze the observation of nausea/vomiting in NSCLC patients receiving postoperative adjuvant chemotherapy. We found that compared to "Cisplatin_Vinorelbine", "Carboplatin_Gemcitabine" (Log OR=45.59, 95%CI 1.29 to 154.91), "Cisplatin_Docetaxel" (Log OR=44.90, 95%CI 1.36 to 154.30), "Cisplatin_Gemcitabine" (Log OR=45.38, 95%CI 1.70 to 154.96) had a higher incidence of nausea/vomiting. However, the nausea/vomiting was more

For peer review only

frequently observed in patients receiving “Cisplatin_Vinorelbine” than “Carboplatin_Pemetrexed” (Log OR=-27.02, 95%CI -92.63 to -1.18). Furthermore, there was no significant difference between the remaining chemotherapy regimens and “Cisplatin_Vinorelbine”(**Figure 6E**).

For peer review only

Discussion

Recent advances in precision medicine have shaped the future of adjuvant therapy for patients with completely resected NSCLC [19]. However, for patients not applicable to targeted therapy or immunotherapy, cytotoxicity chemotherapy is still recommended by guidelines as the standard of care. It is of great significance to investigate the best platinum regimen in the postoperative adjuvant chemotherapy setting for early-stage NSCLC. Our results showed that adjuvant postoperative chemotherapy was beneficial to improve the RFS and OS compared with the observation arm. Among these chemotherapy regimens, the "Cisplatin_Vinorelbine" arm was an effective therapeutic method to improve survival with tolerable toxicity; wherase the "Cisplatin_Pemetrexed" arm did not show advantage over the other therapeutic methods in improving survival. Also, the "Cisplatin_Etoposide" arm contributed to improving the RFS and OS rate for patients with resected NSCLC.

A meta-analysis, published in 1995, demonstrated that postoperative chemotherapy had a significant advantage in 5-year survival compared with the observation group, which opened up a new era of postoperative chemotherapy [43]. Of note, an updated analysis published in 2010 further confirmed the clinical value of postoperative adjuvant chemotherapy, with or without radiotherapy, for patients with operable NSCLC[44]. Moreover, a pooled analysis also indicated the promising role of cisplatin-based adjuvant chemotherapy on the 5-year survival [6]. Based on these findings, it is universally acknowledged that adjuvant cytotoxicity chemotherapy integrating platinum and third-generation agents is conducive to the improvement of postsurgical

For peer review only

survival for NSCLC patients. In the present study, the survival benefit was also observed in the platinum adjuvant chemotherapy setting. The adjuvant chemotherapy regimens were not limited to cisplatin-based chemotherapy, which extended to cisplatin/carboplatin in combination with other third-generation agents. Furthermore, the peak recurrence of patients with early-stage NSCLC is 2 years later after surgery and a higher 5-year RFS rate represented the lower possibility of postoperative relapse. In our study, not only 5-year RFS and OS benefit but the improvement of RFS, OS, 2-year RFS and OS rate, and 3-year RFS and OS rate were observed in the adjuvant chemotherapy group. These significant survival advantages could be explained in part by the fact that postoperative adjuvant chemotherapy could decrease the presence of micro-metastases of cancer cells.

High heterogeneity existed in evaluating the RFS and OS rate between the chemotherapy and observation group. Given that heterogeneity in some analyses may impose a distinct effect on the synthesis results, the mechanisms behind it deserve to be figured out. More importantly, this heterogeneity may implicate the optimal subgroup of patients who could obtain the clinical benefits from postoperative adjuvant chemotherapy. As the JCOG9304[23] indicated, postoperative cisplatin with vindesine chemotherapy did not show efficacious in cases of resected N2 NSCLC. Meanwhile, the JLCSSG trial also failed to elucidate the therapeutic benefits of postoperative cisplatin plus vindesine chemotherapy, since the eligible patients in the trial were limited to be stage III[25]. Therefore, it is reasonable to infer that patients with stage

For peer review only

III (T3 or any N2) may derive very limited benefits from the adjuvant "Cisplatin_Vindesine".

According to the subgroup analysis conducted by LACE in 2010, "Cisplatin_Vinorelbine" was an effective therapeutic method for the improvement of survival with a manageable toxicity profile [8]. Over the few decades, the introduction of the other third-generation cytotoxicity chemotherapy regimens such as Pemetrexed and Docetaxel have brought into the development of platinum adjuvant chemotherapy [10-12]. Noteworthy, regardless of the absence of level 1 data in the adjuvant setting, due to its gold status in non-squamous advanced NSCLC with superior efficacy and low tolerability, "Cisplatin_Pemetrexed" is still reasonably recommended as a standard of care for patients with early-stage resectable non-squamous NSCLC [13, 14]. However, our results indicated that "Cisplatin_Pemetrexed" did not show its significant survival advantages over the observation arm in the adjuvant setting. In addition, a subgroup analysis conducted by a large clinical trial---ECOG1505, did not find a significant difference among the four platinum-based chemotherapy regimens : "Cisplatin_Vinorelbine", "Cisplatin_Docetaxel", "Cisplatin_Gemcitabine" and "Cisplatin_Pemetrexed" [45]. Of note, the absolute effect of adjuvant cytotoxicity chemotherapy on stage IB and squamous patients is still under debated [10, 46]. Hence, it seemed that the "Cisplatin_Vinorelbine" arm still played a vital role in the adjuvant setting for patients with NSCLC since its efficacy was not influenced by the histology or stage [8]. By contrast, the negative results of "Cisplatin_Pemetrexed" implies the restrictions of its application in the adjuvant chemotherapy setting for patients with

For peer review only

squamous. Moreover, some biomarkers such as the excision repair cross-complementation group 1 (ERCC1), b-Tubulin, and Mucin hold the potential to predict the efficacy of cytotoxicity chemotherapy in the adjuvant setting [47-49]. Therefore, the heterogeneity of biological characteristics of each patient imposed confounding factors to the comparisons of different chemotherapy regimens.

According to our analysis, "Cisplatin_Etoposide", the gold chemotherapy regimens for small cell lung cancer, did have a significantly higher survival rate over observation arm in the patients with early-stage resectable NSCLC. Mario Roselli et.al conducted a 10-year follow-up clinical trials to demonstrate the effect of "Cisplatin_Etoposide" on the reduction of recurrences and the extension of long-term survival, which enrolled only patients with stage I NSCLC and adopted the relatively high dose of Cisplatin (100mg/m2) and Etoposide (120mg/m2)[33]. Hence, considering the potential bias, the positive results of "Cisplatin_Etoposide" need to be interpreted with caution.

Anti-angiogenic therapy, such as bevacizumab and endostar, hold the potential to improve the efficacy of adjuvant chemotherapy probably through inhibiting the growth of micro-metastatic cancer cells and increasing the sensitivity of tumor cells to chemotherapy [27, 50, 51]. However, our results showed that the combination of chemotherapy regimens and endostar did not show advantage over a single platinum-based chemotherapy arm. Furthermore, according to the results of ECOG1505 trial, there was no significant difference between the adjuvant chemotherapy with or without bevacizumab for patients with resected NSCLC [45]. One of the reasons to explain these negative results might be the heterogeneity in the expression of vascular

For peer review only

endothelial growth factor (VEGF). Patients with high VEGF expression tend to obtain more benefits from anti-angiogenic therapy than the low VEGF expression ones [27]. In terms of the safety of adjuvant chemotherapy, the incidence of hematological toxicities and nausea/vomiting, which was greatly influenced by the dose and schedules, were not significantly higher in the "Cisplatin_Vinorelbine" arm than other chemotherapy. Noteworthy, the schedules in the adjuvant setting was quite different from the one utilized in the advanced NSCLC patients[52]. Therefore, "Cisplatin_Vinorelbine" could be recommended as an adjuvant cytotoxicity chemotherapy regimen with an acceptable safety profile. On the contrary, the incidence of anemia and thrombocytopenia in the "Carboplatin_Pemetrexed" group was significantly higher than the "Cisplatin_Vinorelbine" group. Both the "Carboplatin" and "Pemetrexed" are characterized as relatively safe cytotoxicity chemotherapy regimens and recommended to be utilized for patients with medical commodities. The contradictory outcomes could be explained in part by the relatively lower dose of "Cisplatin_Vinorelbine".

To the best of our knowledge, the current study was the first to directly compare the efficacy and safety of different adjuvant chemotherapy regimens for NSCLC patients. Despite the strengths, it was evident that there still existed some limitations in the present study. Firstly, for nearly half of the enrolled studies, the risk of bias in certain items remained unclear and the quality of them could not be evaluated. Secondary, due to the lack of specific data, the subgroup analysis stratified by stage and pathology could not be performed. Thus, it is difficult to determine the optimal subgroup populations

For peer review only

who may obtain the most benefits from certain cytotoxicity chemotherapy regimens. Furthermore, we did not incorporate any targeted or immune-biological therapy into the analysis due to the open-loop of interventions. Last but not at least, although the absolute effect of radiotherapy for patients with completely resected NSCLC was presented recently, we did not conduct a subgroup analysis stratified by postoperative radiotherapy or not as it was undertaken according to every center's policy.

Conclusion

In conclusion, the current study summarized the adjuvant cytotoxicity chemotherapy regimens for patients with early-stage resected NSCLC. Research on adjuvant cytotoxicity chemotherapy might be an out-of-date topic but numerous NSCLC patients could obtain benefit from the optimal cytotoxicity chemotherapy regimen. "Cisplatin_Vinorelbine" had a significant survival advantage with a relatively good safety profile in the adjuvant setting while the "Cisplatin_Pemetrexed" arm was not superior to the other therapeutic methods in improving survival. Our analysis may provide some guiding significance for the clinicians when determining the optimal chemotherapy regimen.

For peer review only

Contributorship statement

Wenfeng Fang: Conceptualization; Lanlan Pang& Jiadi Gan & Yihua Huang: Data curation; Lanlan Pang & Jiadi Gan: Formal analysis; Wenfeng Fang: Funding acquisition; Lanlan Pang: Investigation; Lanlan Pang: Methodology; Wenfeng Fang: Project administration; Lanlan Pang: Resources; Lanlan Pang: Software, Li Zhang &Wenfeng Fang: Supervision; Jiadi Gan: Validation; Lanlan Pang: Visualization; All authors---Lan-Lan Pang, Jia-Di Gan, Yi-Hua Huang, Jun Liao, Yi Lv, Wael-Abdullah-Sultan Ali, Li Zhang, Wen-Feng Fang: Roles/Writing - original draft; All Authors---Lan-Lan Pang, Jia-Di Gan, Yi-Hua Huang, Jun Liao, Yi Lv, Wael-Abdullah-Sultan Ali , Li Zhang, Wen-Feng Fang: Writing - review & editing.

Competing Interest

The authors declare no potential conflicts of interest.

Funding

This work was supported by Chinese National Natural Science Foundation Project grant number 81772476.

Data sharing

No additional data available.

Ethics approval

This study did not require ethics approval as no human participants were involved.

For peer review only

References

1. Sung, H., et al., *Global Cancer Statistics 2020: GLOBOCAN Estimates of Incidence and Mortality Worldwide for 36 Cancers in 185 Countries.* CA Cancer J Clin, 2021. **71**(3): p. 209-249.
2. Zappa, C. and S.A. Mousa, *Non-small cell lung cancer: current treatment and future advances.* Transl Lung Cancer Res, 2016. **5**(3): p. 288-300.
3. Mountain, C.F., *Revisions in the International System for Staging Lung Cancer.* Chest, 1997. **111**(6): p. 1710-7.
4. Goldstraw, P., et al., *The IASLC Lung Cancer Staging Project: Proposals for Revision of the TNM Stage Groupings in the Forthcoming (Eighth) Edition of the TNM Classification for Lung Cancer.* J Thorac Oncol, 2016. **11**(1): p. 39-51.
5. Rosell, R., et al., *The role of chemotherapy in early non-small-cell lung cancer management.* Lung Cancer, 2001. **34 Suppl 3**: p. S63-74.
6. Pignon, J.P., et al., *Lung adjuvant cisplatin evaluation: a pooled analysis by the LACE Collaborative Group.* J Clin Oncol, 2008. **26**(21): p. 3552-9.
7. *Cancer Care Ontario and American Society of Clinical Oncology Adjuvant Chemotherapy and Adjuvant Radiation Therapy for Stages I-IIIA Resectable Non-Small-Cell Lung Cancer Guideline.* J Oncol Pract, 2007. **3**(6): p. 332-335.
8. Douillard, J.Y., et al., *Adjuvant cisplatin and vinorelbine for completely resected non-small cell lung cancer: subgroup analysis of the Lung Adjuvant Cisplatin Evaluation.* J Thorac Oncol, 2010. **5**(2): p. 220-8.
9. Postmus, P.E., et al., *Early and locally advanced non-small-cell lung cancer (NSCLC): ESMO Clinical Practice Guidelines for diagnosis, treatment and follow-up.* Ann Oncol, 2017. **28**(suppl_4): p. iv1-iv21.
10. Strauss, G.M., et al., *Adjuvant paclitaxel plus carboplatin compared with observation in stage IB non-small-cell lung cancer: CALGB 9633 with the Cancer and Leukemia Group B, Radiation Therapy Oncology Group, and North Central Cancer Treatment Group Study Groups.* J Clin Oncol, 2008. **26**(31): p. 5043-51.
11. Chen, Q., et al., *Clinical observation of docetaxel or gemcitabine combined with cisplatin in the chemotherapy after surgery for stage II-III non-small cell lung cancer.* Pak J Med Sci, 2015. **31**(5): p. 1095-8.
12. Kreuter, M., et al., *Randomized phase 2 trial on refinement of early-stage NSCLC adjuvant chemotherapy with cisplatin and pemetrexed versus cisplatin and vinorelbine: the TREAT study.* Ann Oncol, 2013. **24**(4): p. 986-92.
13. Scagliotti, G.V., et al., *Phase III study comparing cisplatin plus gemcitabine with cisplatin plus pemetrexed in chemotherapy-naive patients with advanced-stage non-small-cell lung cancer.* J Clin Oncol, 2008. **26**(21): p. 3543-51.
14. Bray, F., et al., *Global cancer statistics 2018: GLOBOCAN estimates of incidence and mortality worldwide for 36 cancers in 185 countries.* CA Cancer J Clin, 2018. **68**(6): p. 394-424.
15. Ardizzoni, A., et al., *Cisplatin- versus carboplatin-based chemotherapy in first-line treatment of advanced non-small-cell lung cancer: an individual patient data meta-analysis.* J Natl Cancer Inst, 2007. **99**(11): p. 847-57.
16. Gridelli, C., et al., *Chemotherapy of advanced NSCLC in special patient population.* Ann Oncol,

For peer review only

2006. **17 Suppl 5**: p. v72-8.

17. Goss, G.D., et al., *Gefitinib versus placebo in completely resected non-small-cell lung cancer: results of the NCIC CTG BR19 study.* J Clin Oncol, 2013. **31**(27): p. 3320-6.

18. Kelly, K., et al., *Adjuvant Erlotinib Versus Placebo in Patients With Stage IB-IIIA Non-Small-Cell Lung Cancer (RADIANT): A Randomized, Double-Blind, Phase III Trial.* J Clin Oncol, 2015. **33**(34): p. 4007-14.

19. Zhong, W.Z., et al., *Gefitinib versus vinorelbine plus cisplatin as adjuvant treatment for stage II-IIIA (N1-N2) EGFR-mutant NSCLC (ADJUVANT/CTONG1104): a randomised, open-label, phase 3 study.* Lancet Oncol, 2018. **19**(1): p. 139-148.

20. Higgins, J.P.T., *Cochrane handbook for systematic reviews of interventions. Cochrane Collaboration.* 2011. **10**.

21. Salanti, G., A.E. Ades, and J.P. Ioannidis, *Graphical methods and numerical summaries for presenting results from multiple-treatment meta-analysis: an overview and tutorial.* J Clin Epidemiol, 2011. **64**(2): p. 163-71.

22. Fukumoto, S.I., et al., *A randomized phase II trial of cisplatin plus gemcitabine versus carboplatin plus gemcitabine in patients with completely resected non-small cell lung cancer: Hokkaido Lung Cancer Clinical Study Group Trial (HOT0703).* Cancer Chemother Pharmacol, 2020. **86**(1): p. 117-127.

23. Tada, H., et al., *A randomized trial comparing adjuvant chemotherapy versus surgery alone for completely resected pN2 non-small cell lung cancer (JCOG9304).* Lung Cancer, 2004. **43**(2): p. 167-73.

24. Kenmotsu, H., et al., *Randomized Phase III Study of Pemetrexed Plus Cisplatin Versus Vinorelbine Plus Cisplatin for Completely Resected Stage II to IIIA Nonsquamous Non-Small-Cell Lung Cancer.* J Clin Oncol, 2020. **38**(19): p. 2187-2196.

25. Ohta, M., et al., *Adjuvant chemotherapy for completely resected stage III non-small-cell lung cancer. Results of a randomized prospective study. The Japan Clinical Oncology Group.* J Thorac Cardiovasc Surg, 1993. **106**(4): p. 703-8.

26. Kubota, K., et al., *Randomized phase II trial of adjuvant chemotherapy with docetaxel plus cisplatin versus paclitaxel plus carboplatin in patients with completely resected non-small cell lung cancer: TORG 0503.* Lung Cancer, 2020. **141**: p. 32-36.

27. Chen, Z., et al., *Endostar in combination with postoperative adjuvant chemotherapy prolongs the disease free survival of stage IIIA NSCLC patients with high VEGF expression.* Oncotarget, 2017. **8**(45): p. 79703-79711.

28. Zhong, W.Z., et al., *Randomized Study of Adjuvant Docetaxel vs. Observation for Completely Resected Stage IB-IIIa NSCLC with 11 Years' Median Follow-Up.* Journal of Thoracic Oncology, 2017. **12**(1): p. S642-S643.

29. Park, J.H., et al., *Postoperative adjuvant chemotherapy for stage I non-small cell lung cancer.* Eur J Cardiothorac Surg, 2005. **27**(6): p. 1086-91.

30. Barlesi, F., et al., *A randomized trial comparing adjuvant chemotherapy with gemcitabine plus cisplatin with docetaxel plus cisplatin in patients with completely resected non-small-cell lung cancer with quality of life as the primary objective.* Interact Cardiovasc Thorac Surg, 2015. **20**(6): p. 783-90.

31. Dunant, A., J.P. Pignon, and T. Le Chevalier, *Adjuvant chemotherapy for non-small cell lung cancer: contribution of the International Adjuvant Lung Trial.* Clin Cancer Res, 2005. **11**(13 Pt

For peer review only

2): p. 5017s-5021s.

32. Kreuter, M., et al., *Three-Year Follow-Up of a Randomized Phase II Trial on Refinement of Early-Stage NSCLC Adjuvant Chemotherapy with Cisplatin and Pemetrexed versus Cisplatin and Vinorelbine (the TREAT Study).* J Thorac Oncol, 2016. **11**(1): p. 85-93.

33. Roselli, M., et al., *Postsurgical chemotherapy in stage IB nonsmall cell lung cancer: Long-term survival in a randomized study.* Int J Cancer, 2006. **119**(4): p. 955-60.

34. Winton, T., et al., *Vinorelbine plus cisplatin vs. observation in resected non-small-cell lung cancer.* N Engl J Med, 2005. **352**(25): p. 2589-97.

35. Waller, D., et al., *Chemotherapy for patients with non-small cell lung cancer: the surgical setting of the Big Lung Trial.* Eur J Cardiothorac Surg, 2004. **26**(1): p. 173-82.

36. Douillard, J.Y., et al., *Adjuvant vinorelbine plus cisplatin versus observation in patients with completely resected stage IB-IIIA non-small-cell lung cancer (Adjuvant Navelbine International Trialist Association [ANITA]): a randomised controlled trial.* Lancet Oncol, 2006. **7**(9): p. 719-27.

37. Schmid-Bindert, G., et al., *A randomized Phase 2 study of pemetrexed in combination with cisplatin or carboplatin as adjuvant chemotherapy in patients with completely resected stage IB or II Non-Small-Cell Lung Cancer.* Lung Cancer, 2015. **90**(3): p. 397-404.

38. Felip, E., et al., *Preoperative chemotherapy plus surgery versus surgery plus adjuvant chemotherapy versus surgery alone in early-stage non-small-cell lung cancer.* J Clin Oncol, 2010. **28**(19): p. 3138-45.

39. *A randomized controlled study of postoperative adjuvant chemoimmunotherapy of resected non-small cell lung cancer with IL2 and LAK cells: H.Kimura, Y.Yamaguchi, T.Fujisawa, M.Baba, and M.Shiba Institute of Pulmonary Cancer Research, School of Medicine, Chi %J Lung Cancer.* 1991.

40. Park, J.H., *P2-202: Postoperative adjuvant therapy for stage IIIA non small cell lung cancer.* Journal of Thoracic Oncology, 2007. **2**(8, Supplement): p. S651.

41. Park, J.H., et al., *Postoperative adjuvant chemotherapy for stage I non-small cell lung cancer ☆.* European Journal of Cardio-Thoracic Surgery, 2005. **27**(6): p. 1086-1091.

42. Scagliotti, G.V., et al., *Randomized study of adjuvant chemotherapy for completely resected stage I, II, or IIIA non-small-cell Lung cancer.* J Natl Cancer Inst, 2003. **95**(19): p. 1453-61.

43. *Chemotherapy in non-small cell lung cancer: a meta-analysis using updated data on individual patients from 52 randomised clinical trials. Non-small Cell Lung Cancer Collaborative Group.* Bmj, 1995. **311**(7010): p. 899-909.

44. Arriagada, R., et al., *Adjuvant chemotherapy, with or without postoperative radiotherapy, in operable non-small-cell lung cancer: two meta-analyses of individual patient data.* Lancet, 2010. **375**(9722): p. 1267-77.

45. Wakelee, H.A., et al., *Adjuvant chemotherapy with or without bevacizumab in patients with resected non-small-cell lung cancer (E1505): an open-label, multicentre, randomised, phase 3 trial.* Lancet Oncol, 2017. **18**(12): p. 1610-1623.

46. Scagliotti, G., et al., *The differential efficacy of pemetrexed according to NSCLC histology: a review of two Phase III studies.* Oncologist, 2009. **14**(3): p. 253-63.

47. Friboulet, L., et al., *ERCC1 isoform expression and DNA repair in non-small-cell lung cancer.* N Engl J Med, 2013. **368**(12): p. 1101-10.

48. Graziano, S.L., et al., *Prognostic significance of mucin and p53 expression in stage IB non-small*

For peer review only

cell lung cancer: a laboratory companion study to CALGB 9633. J Thorac Oncol, 2010. **5**(6): p. 810-7.

49. Reiman, T., et al., *Cross-validation study of class III beta-tubulin as a predictive marker for benefit from adjuvant chemotherapy in resected non-small-cell lung cancer: analysis of four randomized trials.* Ann Oncol, 2012. **23**(1): p. 86-93.
50. Folkman, J., *Antiangiogenesis in cancer therapy--endostatin and its mechanisms of action.* Exp Cell Res, 2006. **312**(5): p. 594-607.
51. Limaverde-Sousa, G., C. Sternberg, and C.G. Ferreira, *Antiangiogenesis beyond VEGF inhibition: a journey from antiangiogenic single-target to broad-spectrum agents.* Cancer Treat Rev, 2014. **40**(4): p. 548-57.
52. Caffo, O., et al., *An evaluation of the pharmacokinetics and clinical use of vinorelbine for NSCLC treatment.* Expert Opin Drug Metab Toxicol, 2013. **9**(8): p. 1037-51.

For peer review only

Figure list

Figure 1. Flow diagram of enrolled studies selection.

Figure 2.

A) The efficacy of platinum-based postoperative adjuvant chemotherapy in improving the RFS compared with the observation group.

B) 2-year RFS rate of the chemotherapy arm in comparison with the observation arm.

C) 3-year RFS rate of the chemotherapy arm in comparison with the observation arm.

D) 5-year RFS rate of the chemotherapy arm in comparison with the observation arm

Figure 3.

A) Network evidence of the comparisons for the best adjuvant chemotherapy concerning RFS. In the Network evidence figure, each node represents a type of treatment. Solid lines connect treatments that are directly compared in at least one study. The thickness of connections varies based on the number of studies involved in a comparison.

B) Forest plots of the comparisons for the different cytotoxicity chemotherapy regimens concerning RFS.

C) The league table of the comparisons for the different cytotoxicity chemotherapy regimens concerning RFS.

D) SUCRA value of every cytotoxicity chemotherapy regimens concerning RFS.

Figure 4.

A) The efficacy of platinum-based postoperative adjuvant chemotherapy in improving the OS compared with the observation group.

For peer review only

665 **B)** 2-year OS rate of the chemotherapy arm compared to the observation arm.

666 **C)** 3-year OS rate of the chemotherapy arm compared to the observation arm.

667 **D)** 5-year OS rate of the chemotherapy arm compared to the observation arm.

668 **Figure 5.**

669 **A)** Network evidence of the comparisons for the best adjuvant chemotherapy concerning OS.

671 **B)** Forest plots of the comparisons for the different cytotoxicity chemotherapy regimens concerning OS.

673 **C)** The league table of the comparisons for the different cytotoxicity chemotherapy regimens concerning OS.

675 **D)** SUCRA value of every cytotoxicity chemotherapy regimens concerning OS.

676 **Figure 6.**

677 **A)** Forest plots of the comparisons for the different cytotoxicity chemotherapy regimens concerning the presence of anemia.

679 **B)** Forest plots of the comparisons for the different cytotoxicity chemotherapy regimens concerning the presence of neutropenia.

681 **C)** Forest plots of the comparisons for the different cytotoxicity chemotherapy regimens concerning the presence of thrombocytopenia .

683 **D)** Forest plots of the comparisons for the different cytotoxicity chemotherapy regimens concerning the presence of febrile neutropenia.

685 **E)** Forest plots of the comparisons for the different cytotoxicity chemotherapy regimens concerning the presence of nausea/vomiting.

For peer review only

Supplementary Figure list

Supplementary Figure 1.

A) Risk of bias graph.

B) Risk of bias summary

Supplementary Figure 2.

A) Forest plots of the comparisons for the different cytotoxicity chemotherapy regimens with regard to 2-year RFS rate.

B) Forest plots of the comparisons for the different cytotoxicity chemotherapy regimens with regard to 3-year RFS rate.

C) Forest plots of the comparisons for the different cytotoxicity chemotherapy regimens with regard to 5-year RFS rate.

Supplementary Figure 3.

A) Forest plots of the comparisons for the different cytotoxicity chemotherapy regimens with regard to 2-year OS rate.

B) Forest plots of the comparisons for the different cytotoxicity chemotherapy regimens with regard to 3-year OS rate.

C) Forest plots of the comparisons for the different cytotoxicity chemotherapy regimens with regard to 5-year OS rate.

For peer review only

Table 1 Basic characteristics of enrolled studies and participants.

Trail	Publication year	Country of region	Chemotherapy regimes	Sample size	Median follow-up (months)	Phase	Radiotherapy	Patients characteristics (Pathology;stage,PS)	Age	Sex
ANITA	2006	Multi-Center	**Cisplatin**100mg/m2+**Vinorelbine**30mg/m2 VS.**Observation**	840(407vs.433)	76	III	Not mandatory.	NSCLC; Stage IB – IIIA	59 (32 – 75) VS.59 (18 – 75)	85%vs.87%
Big-lung-trial	2003	UK	**Cisplatin**80mg/m2+**Vinorelbine**30mg/m2 VS.**Observation**	66(37VS.29)	58.8	---	Not mandatory	NSCLC;Stage I – III	/	/
CALGB 9633	2004	North Carolina	**Carboplatin**(AUC-6) + **Paclitaxel** 200 mg/m2 VS. **Observation**	344(173VS.171)	74	III	Not-mentioned	NSCLC; T2 with pathologically negative lymph nodes	61(34-78) VS.62(40-81) (range)	65%VS.63%
CSLC0201	2016	China	**Carboplatin**+**Docetaxel** VS.**Observation**	82(43vs.39)	132	---	Not-mentioned	NSCLC; Stage IB-IIIA	/	/
NATCH	2010	Multi-Center	**Carboplatin**(AUC-6.0)**+Paclitaxel**200mg/m2 VS.**Observation**	423(211VS.212)	51	III	Postoperative radiotherapy was allowed in patients with pathologic N2 disease.	NSCLC ; Stage IA with tumor size more than 2 cm, IB, II, or T3N1	64(33-81) VS.64(36-89) (range)	86%VS.88%
Fabrice Barlesi et.al	2015	France	**Cisplatin**75mg/m2+**Gemcitabine**1250mg/m2VS. **Cisplatin**75mg/m2+**Docetaxel**75mg/m2	136(67VS.69)	20.2	---	Not mandatory.	NSCLC; Stage IB-III	57 (44 – 74) VS.57 (36 – 71)	75%vs.74%
HOT0703	2020	Japan	**Cisplatin**40mg/m2+**Gemcitabine**1000mg/m2 VS.**Carboplatin**(AUC-5)+**Gemcitabine** 1000mg/m2	102(51vs.51)	69.6	II	Not-mentioned	NSCLC; Stage IB-IIIA	63(40-72) VS.64(36-74) (range)	67%VS.63%
IALT	2004	France	**Cisplatin**100mg/m2+**Vinorelbine**30mg/m2VS .**Observation**	500(248VS.262)	49.2	---	Not allowed.	NSCLC; Stage I, II, or III	/	/
JBR10	2005;2010	North American	**Cisplatin**+**Vinorelbine**VS. **Observation**	482(240vs.242)	111.6	III	Not-mentioned	NSCLC; Stage IB (T2N0) or II (T1-2N1)	61(35-82) VS.60.5(34-78)	64%VS.66%

For peer review only

JCOG9304	2003	Japan	**Cisplatin**80mg/m2+**Vindesine**3mg/m2 VS.**Observation**	159(59VS.60)	---	---	Not-mentioned	NSCLC;N2	62 (41—75) VS.62 (43—74)	68%VS.62%
Jing Wang et.al	2012	China	**Cisplation**80mg/m2+**Vinorelbine**30mg/m2 VS.**Observation**	451(225VS.226)	46	---	Not-mentioned	NSCLC;Stage I, II, and IIIA	55(38-83) VS.58(38-82)	71%VS.75%
JIPANG	2020	Japan	**Cisplatin**75mg/m2+**Pemetrexed**500mg/m2VS. **Cisplatin**80mg/m2+**Vinorelbine**25mg/m2	804(402VS.402)	45.2	III	Not allowed.	Non-squamous NSCLC; N2 Stage II or IIIA	65(58-69) VS. 64 (57-67)	60%VS.58%
Mario Roselli et.al	2006	Italy	**Cisplatin**100mg/m2+**Etoposide**120mg/m2VS. **Observation**	140(70VS.70)	40.31	---	Not-mentioned	NSCLC;Stage IB disease (pT2N0)	64.7±9.9 VS.62.9±9.2	91%VS.76%
JLCSSG	1993	Japan	**Cisplatin**80mg/m2+**Vindesine** 3mg/m2VS.**Observation**	181(90VS.91)	31.2	---	Not-mentioned	NSCLC; Stage III	56.3 ± 9.1 VS.58.9 ± 8.4	77%S. 87%
Qiuqiang Chen et.al	2015	China	**Cisplatin**75mg/m2+**Docetaxel**75mg/m2VS. **Cisplatin**75mg/m2+**Gemcitabine**1250mg/m2	92(45vs.47)	22	---	Not-mentioned	NSCLC; Stage II to III	55(32-67) VS.56(31-67) (range)	84%VS.87%
Schmid-Binder Geraldt et.al	2015	Germany, France, and Spain	**Cisplatin**75mg/m2+**Pemetrexed**500mg/m2VS. **Carboplatin**(AUC-5)+**Pemetrexed** 500 mg/m2	112(63VS.59)	---	II	Not-mentioned	NSCLC; Stage IB, IIA or IIB	61(44-75) VS.59(43-69)	78%VS.70%
TORG0503	2019	Japan	**Cisplatin**80mg/m2+**Docetaxel**60mg/m2VS. **Carboplatin**AUC-6+**Paclitaxel** 200 mg/m2	111(58vs.53)	---	II	Not-mentioned	NSCLC; Stage IB, II and IIIA	63(33-70) VS.59(34-70)	60%VS.66%
TREAT	2015	German	**Cisplatin**50mg/m2+**Vinorelbine**25mg/m2VS. **Cisplatin**75mg/m2+**Pemetrexed**500mg/m2	132(67vs.65)	36m	II	Not allowed.	NSCLC; Stages IB, IIA, IIB,	58 (40 – 73) VS.60 (38 – 74)	72%VS. 77%
Yanzhuo Yang et.al	2012	China	**Carboplatin**(AUC=5-6)+**Docetaxel**75mg/m2+ **Endostar**15mgVS.**Carboplatin**+**Docetaxel**	76(38vs.38)	22	---	Not-mentioned	NSCLC;Stage IB-III	55.6(36-74) VS.60.2(45-77)	31%vs.27%
Zhiwei Chen et.al	2017	China	**Cisplatin**75mg/m2+**Vinorelbine**25mg/m2+ **Endostar**7.5mg/m2VS.**Cisplatin**+**Vinorelbine**	250(125vs.125)	60	III	Not-mentioned	NSCLC; Stage IB to IIIA	58(33-75) VS.55.5(37-71)	66%VS.67%

NSCLC: non-small cell lung cancer; PS: performance status; AUC: area under the curve

For peer review only

For peer review only - http://bmjopen.bmj.com/site/about/guidelines.xhtml

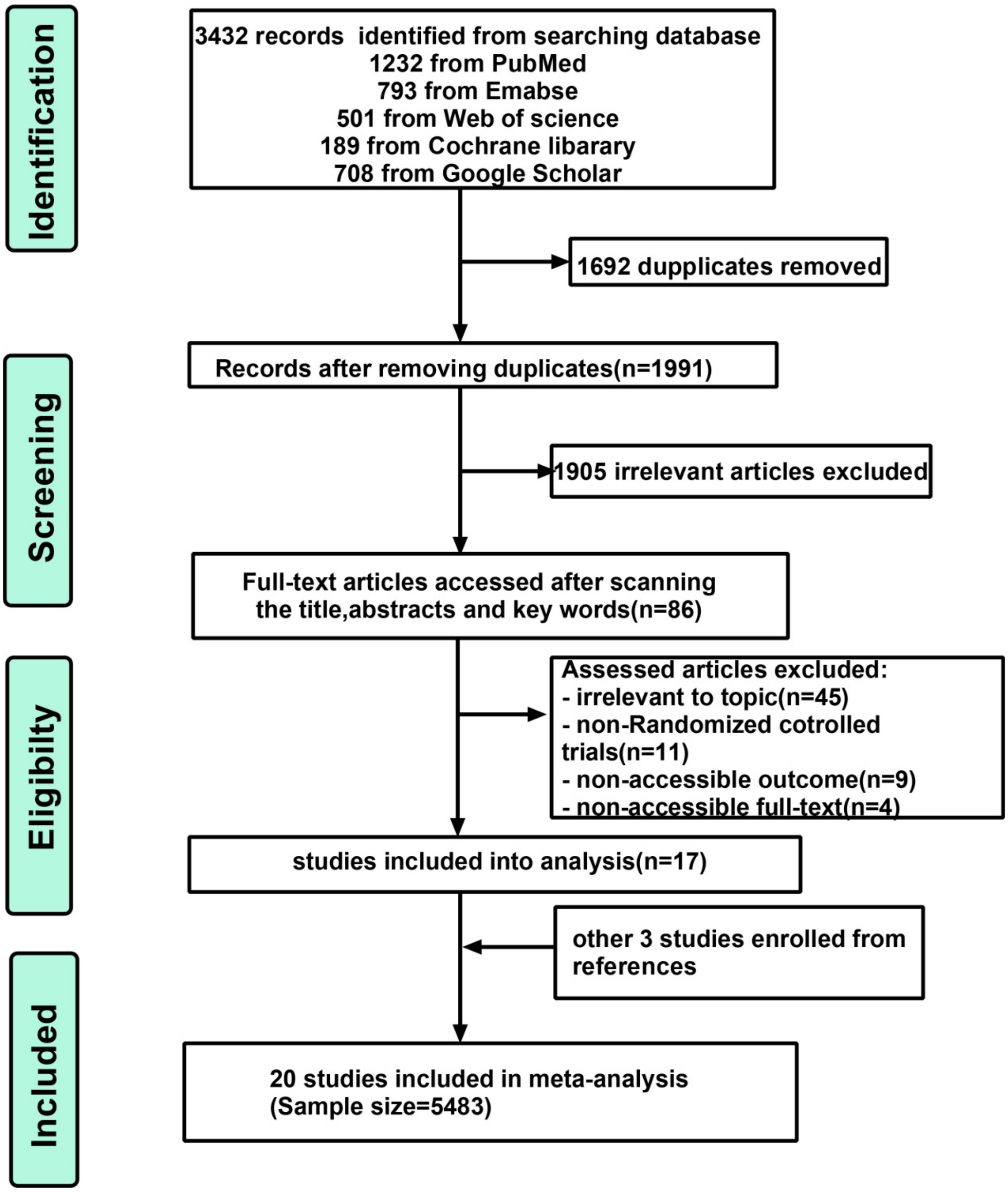

Figure 1. Flow diagram of enrolled studies selection.

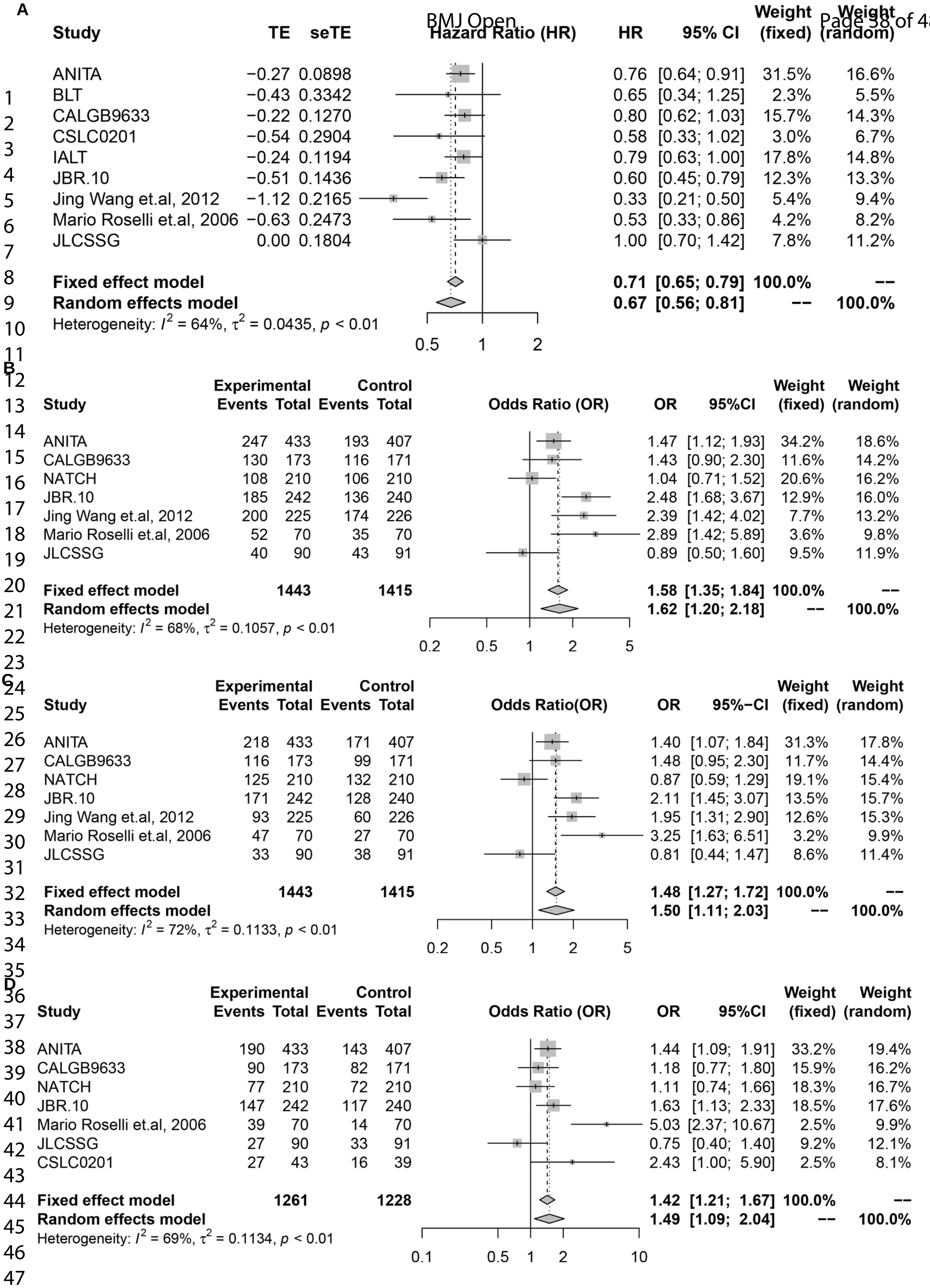

Figure 2.
A) The efficacy of platinum-based postoperative adjuvant chemotherapy in improving the relapse-free survival (RFS) compared with the observation group.
B) 2-year; **C)** 3-year ; **D)** 5-year RFS rate of the chemotherapy arm in comparison with the observation arm.

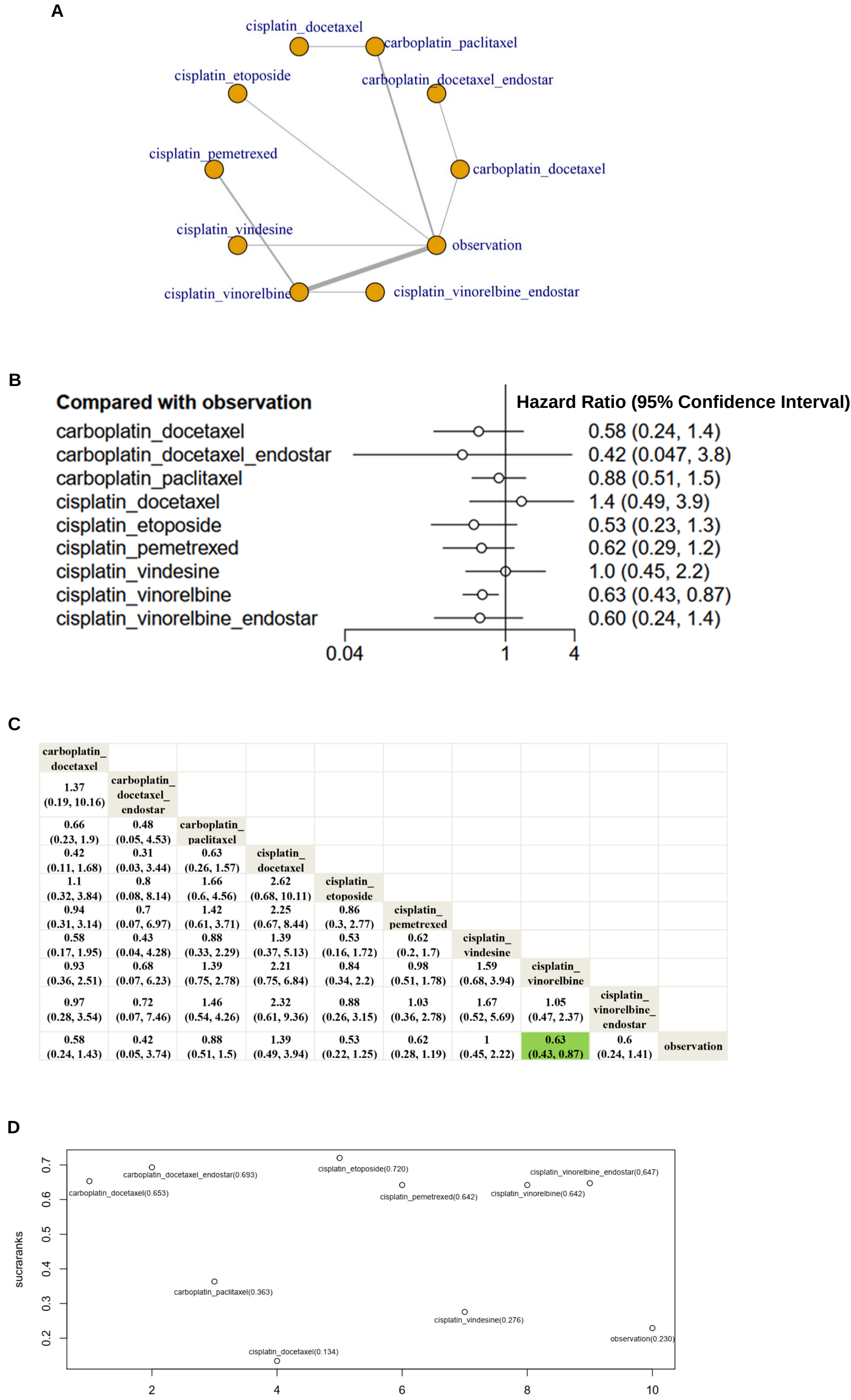

carboplatin_ docetaxel									
1.37 (0.19, 10.16)	carboplatin_ docetaxel_ endostar								
0.66 (0.23, 1.9)	0.48 (0.05, 4.53)	carboplatin_ paclitaxel							
0.42 (0.11, 1.68)	0.31 (0.03, 3.44)	0.63 (0.26, 1.57)	cisplatin_ docetaxel						
1.1 (0.32, 3.84)	0.8 (0.08, 8.14)	1.66 (0.6, 4.56)	2.62 (0.68, 10.11)	cisplatin_ etoposide					
0.94 (0.31, 3.14)	0.7 (0.07, 6.97)	1.42 (0.61, 3.71)	2.25 (0.67, 8.44)	0.86 (0.3, 2.77)	cisplatin_ pemetrexed				
0.58 (0.17, 1.95)	0.43 (0.04, 4.28)	0.88 (0.33, 2.29)	1.39 (0.37, 5.13)	0.53 (0.16, 1.72)	0.62 (0.2, 1.7)	cisplatin_ vindesine			
0.93 (0.36, 2.51)	0.68 (0.07, 6.23)	1.39 (0.75, 2.78)	2.21 (0.75, 6.84)	0.84 (0.34, 2.2)	0.98 (0.51, 1.78)	1.59 (0.68, 3.94)	cisplatin_ vinorelbine		
0.97 (0.28, 3.54)	0.72 (0.07, 7.46)	1.46 (0.54, 4.26)	2.32 (0.61, 9.36)	0.88 (0.26, 3.15)	1.03 (0.36, 2.78)	1.67 (0.52, 5.69)	1.05 (0.47, 2.37)	cisplatin_ vinorelbine_ endostar	
0.58 (0.24, 1.43)	0.42 (0.05, 3.74)	0.88 (0.51, 1.5)	1.39 (0.49, 3.94)	0.53 (0.22, 1.25)	0.62 (0.28, 1.19)	1 (0.45, 2.22)	0.63 (0.43, 0.87)	0.6 (0.24, 1.41)	observation

Figure 3.
A) Network evidence of the comparisons for the best adjuvant chemotherapy with regard to Relapse-free Survival (RFS).
B) Forest plots of the comparisons for the different cytotoxicity chemotherapy regimens with regard to RFS.
C) The league table of the comparisons for the different cytotoxicity chemotherapy regimens with regard to RFS.
D) Surface under the cumulative ranking curve (SUCRA) value of every cytotoxicity chemotherapy regimens with regard to RFS.

For peer review only - http://bmjopen.bmj.com/site/about/guidelines.xhtml

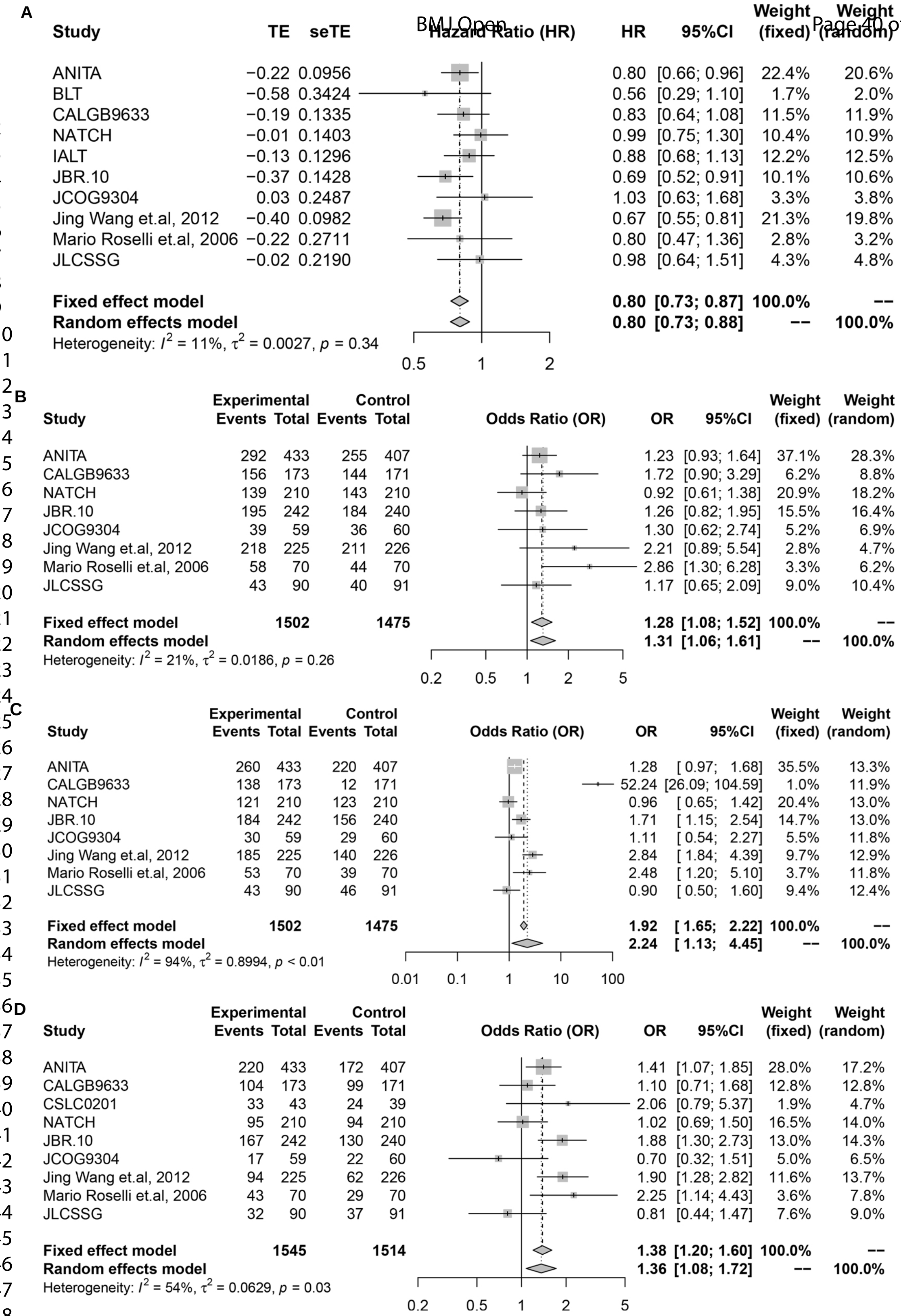

Figure 4.
A) The efficacy of platinum-based postoperative adjuvant chemotherapy in improving the Overall Survival (OS) compared with the observation group.
B) 2-year ; **C)** 3-year; **D)** 5-year OS rate of the chemotherapy arm compared to the observation arm.

A

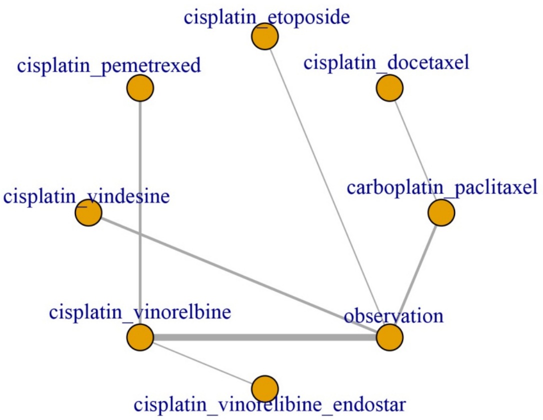

B

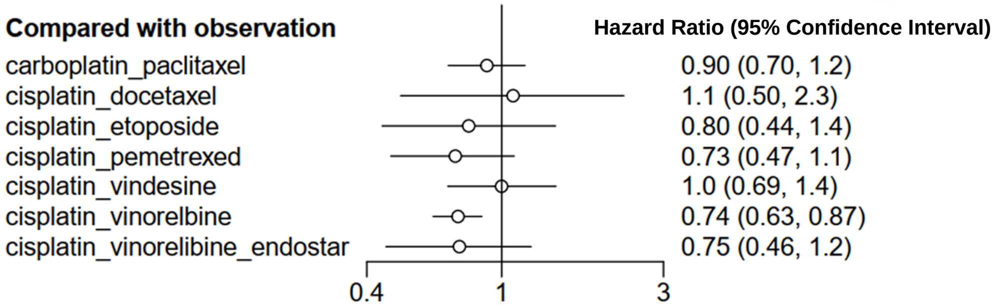

C

carboplatin_ docetaxel									
1.4 (0.19, 10.25)	carboplatin_ docetaxel_ endostar								
0.66 (0.23, 1.9)	0.48 (0.05, 4.67)	carboplatin_ paclitaxel							
0.42 (0.11, 1.68)	0.3 (0.03, 3.47)	0.63 (0.26, 1.57)	cisplatin_ docetaxel						
1.1 (0.32, 3.81)	0.78 (0.08, 8.43)	1.65 (0.6, 4.56)	2.61 (0.67, 10.04)	cisplatin_ etoposide					
0.95 (0.31, 3.14)	0.68 (0.07, 7.09)	1.42 (0.61, 3.68)	2.26 (0.66, 8.36)	0.86 (0.3, 2.76)	cisplatin_ pemetrexed				
0.58 (0.17, 1.95)	0.42 (0.04, 4.4)	0.88 (0.33, 2.29)	1.38 (0.37, 5.13)	0.53 (0.16, 1.71)	0.62 (0.2, 1.71)	cisplatin_ vindesine			
0.93 (0.36, 2.51)	0.67 (0.07, 6.36)	1.39 (0.75, 2.76)	2.21 (0.75, 6.85)	0.85 (0.34, 2.21)	0.98 (0.51, 1.78)	1.59 (0.68, 3.93)	cisplatin_ vinorelbine		
0.97 (0.28, 3.56)	0.7 (0.07, 7.71)	1.46 (0.54, 4.28)	2.32 (0.6, 9.34)	0.89 (0.27, 3.13)	1.03 (0.36, 2.79)	1.67 (0.52, 5.67)	1.05 (0.47, 2.36)	cisplatin_ vinorelbine_ endostar	
0.58 (0.23, 1.44)	0.42 (0.05, 3.84)	0.88 (0.51, 1.5)	1.39 (0.48, 3.94)	0.53 (0.23, 1.25)	0.62 (0.28, 1.19)	1 (0.45, 2.22)	0.63 (0.43, 0.87)	0.6 (0.24, 1.4)	observation

D

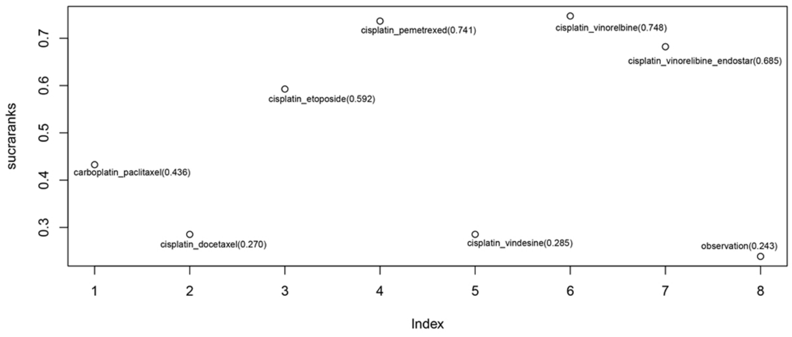

Figure 5.
A) Network evidence of the comparisons for the best adjuvant chemotherapy with regard to Overall Survival (OS).
B) Forest plots of the comparisons for the different cytotoxicity chemotherapy regimens with regard to OS.
C) The league table of the comparisons for the different cytotoxicity chemotherapy regimens with regard to OS.
D) Surface under the cumulative ranking curve (SUCRA) value of every cytotoxicity chemotherapy regimens with regard to OS.

For peer review only - http://bmjopen.bmj.com/site/about/guidelines.xhtml

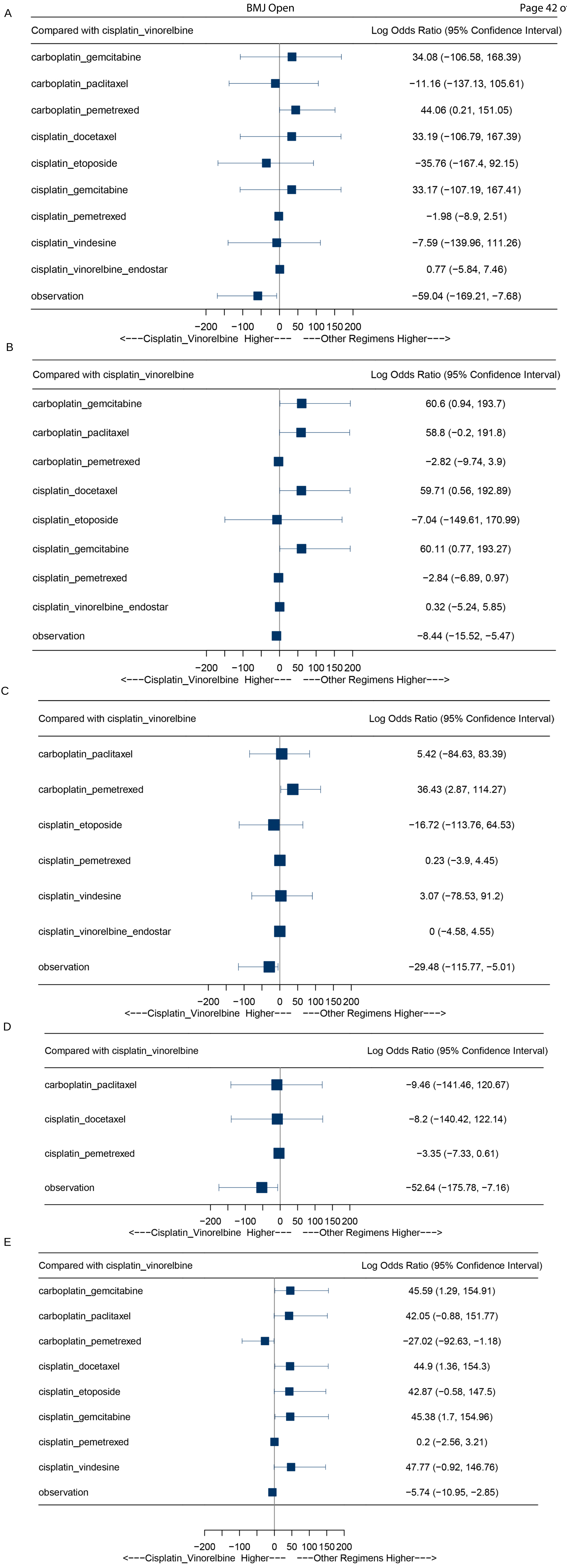

Figure 6. Forest plots of the comparisons for the different cytotoxicity chemotherapy regimens concerning the presence of **A)** anemia;**B)**neutropenia;**C)**thrombocytopenia;**D)**febrile neutropenia;**E)**nausea/vomiting.

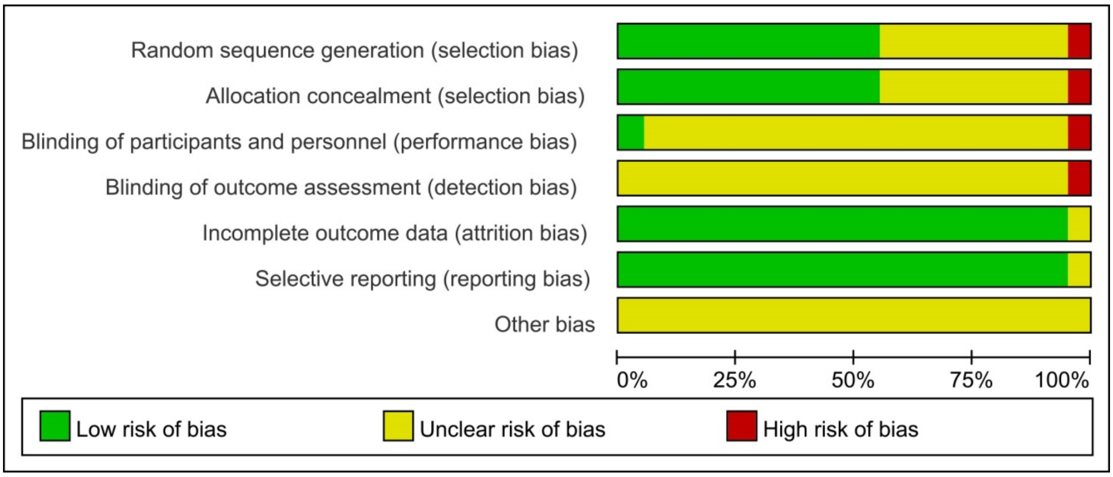

Supplementary Figure 1. A) Risk of bias graph.

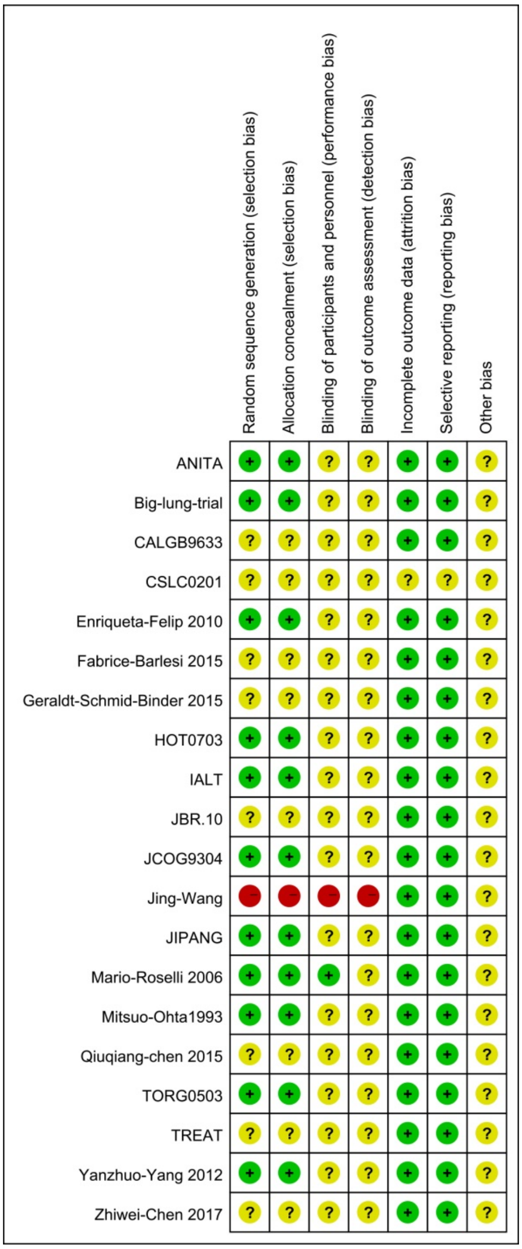

B) Risk of bias summary

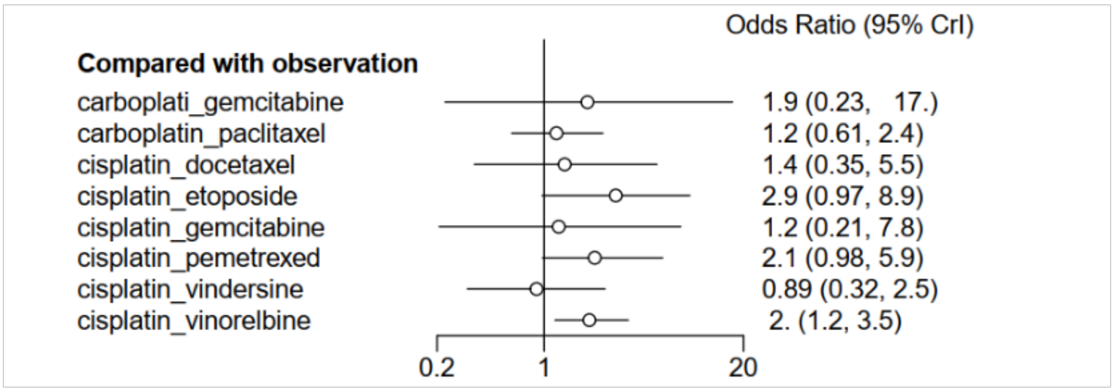

Supplementary.Figure 2.

A) Forest plots of the comparisons for the different cytotoxicity chemotherapy regimens with regard to 2-year RFS rate.

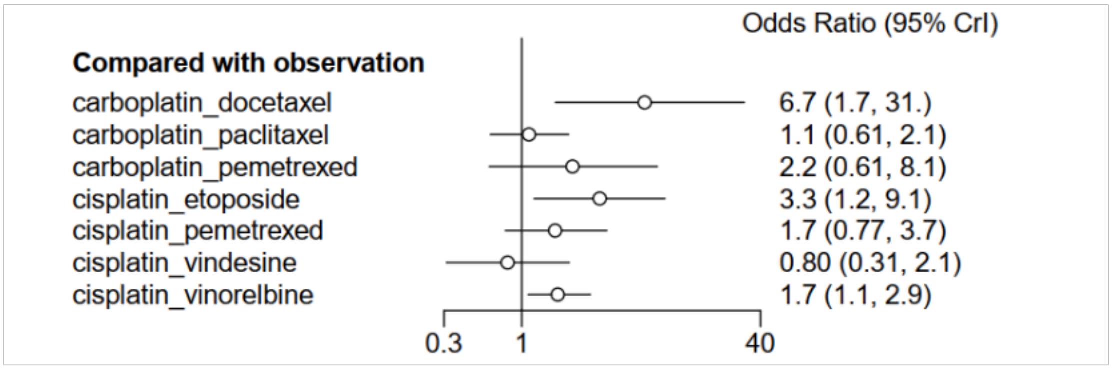

B) Forest plots of the comparisons for the different cytotoxicity chemotherapy regimens with regard to 3-year RFS rate.

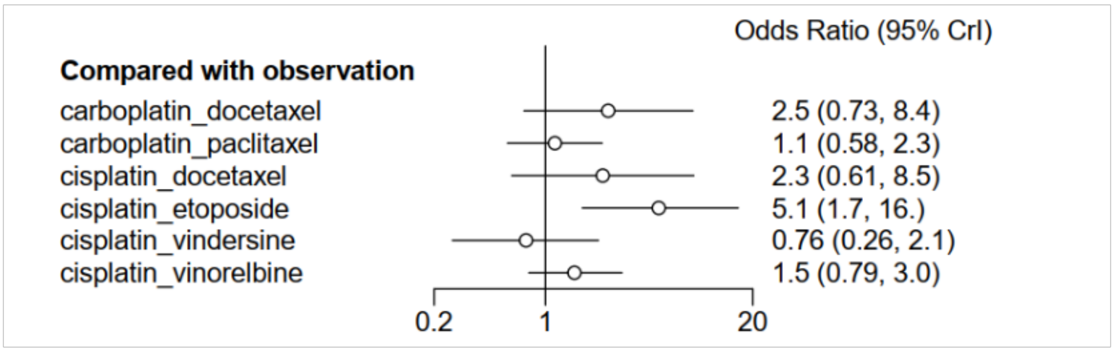

C) Forest plots of the comparisons for the different cytotoxicity chemotherapy regimens with regard to 5-year RFS rate.

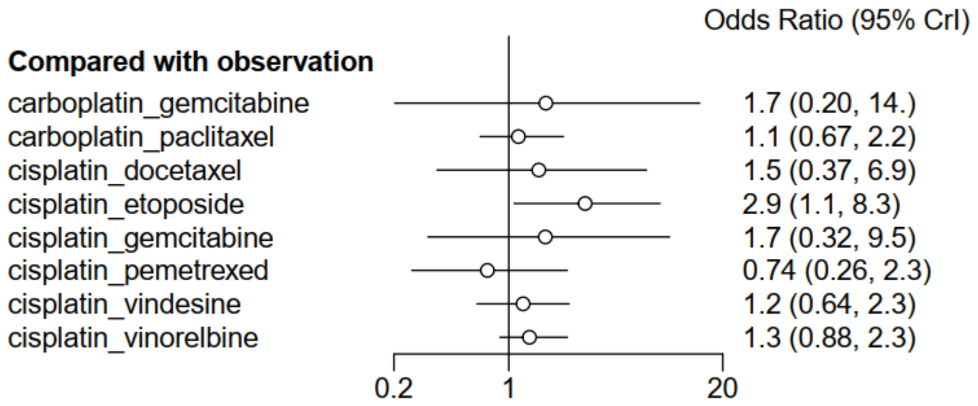

Supplementary.Figure 3.

A) Forest plots of the comparisons for the different cytotoxicity chemotherapy regimens with regard to 2-year OS rate.

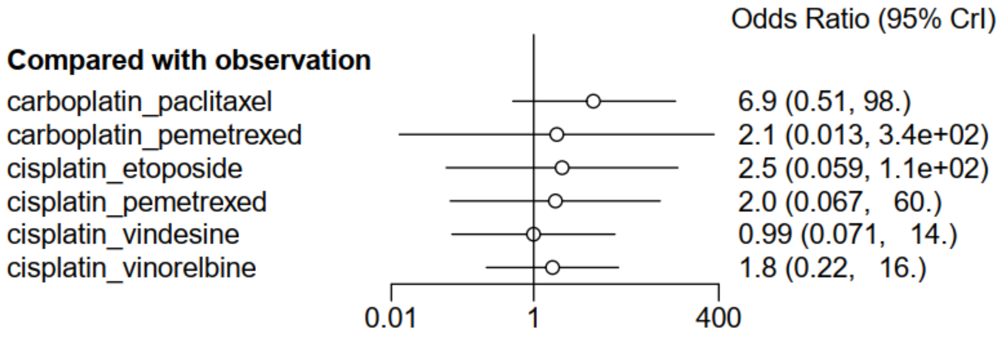

B) Forest plots of the comparisons for the different cytotoxicity chemotherapy regimens with regard to 3-year OS rate.

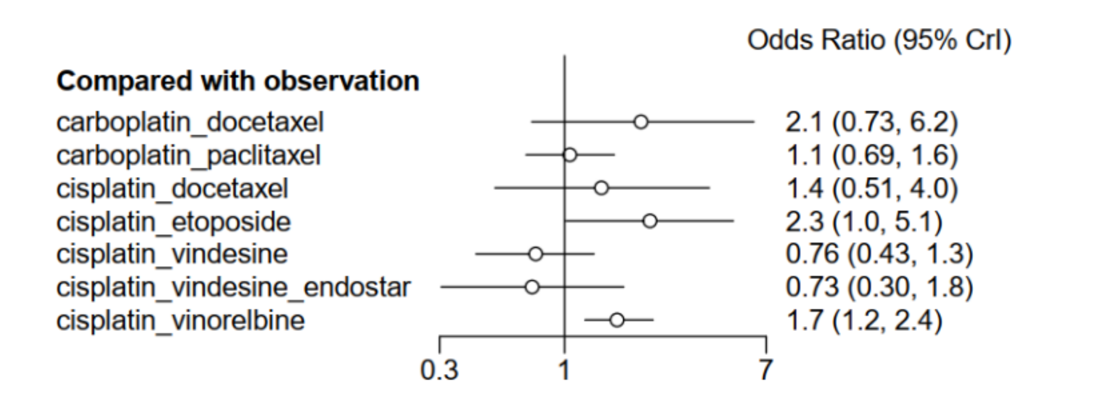

C) Forest plots of the comparisons for the different cytotoxicity chemotherapy regimens with regard to 5-year OS rate.

PRISMA 2009 Checklist

Section/topic	#	Checklist item	Reported on page #
**TITLE**			
Title	1	Identify the report as a systematic review, meta-analysis, or both.	Page 1
**ABSTRACT**			
Structured summary	2	Provide a structured summary including, as applicable: background; objectives; data sources; study eligibility criteria, participants, and interventions; study appraisal and synthesis methods; results; limitations; conclusions and implications of key findings; systematic review registration number.	Page 3-5
**INTRODUCTION**			
Rationale	3	Describe the rationale for the review in the context of what is already known.	Page 6-7
Objectives	4	Provide an explicit statement of questions being addressed with reference to participants, interventions, comparisons, outcomes, and study design (PICOS).	Page 7-8
**METHODS**			
Protocol and registration	5	Indicate if a review protocol exists, if and where it can be accessed (e.g., Web address), and, if available, provide registration information including registration number.	NA
Eligibility criteria	6	Specify study characteristics (e.g., PICOS, length of follow-up) and report characteristics (e.g., years considered, language, publication status) used as criteria for eligibility, giving rationale.	Page 9-10
Information sources	7	Describe all information sources (e.g., databases with dates of coverage, contact with study authors to identify additional studies) in the search and date last searched.	Page 9
Search	8	Present full electronic search strategy for at least one database, including any limits used, such that it could be repeated.	Page 9
Study selection	9	State the process for selecting studies (i.e., screening, eligibility, included in systematic review, and, if applicable, included in the meta-analysis).	Page 9-10
Data collection process	10	Describe method of data extraction from reports (e.g., piloted forms, independently, in duplicate) and any processes for obtaining and confirming data from investigators.	Page 10-11
Data items	11	List and define all variables for which data were sought (e.g., PICOS, funding sources) and any assumptions and simplifications made.	Page 10-11
Risk of bias in individual studies	12	Describe methods used for assessing risk of bias of individual studies (including specification of whether this was done at the study or outcome level), and how this information is to be used in any data synthesis.	Page11
Summary measures	13	State the principal summary measures (e.g., risk ratio, difference in means).	Page 11-12
Synthesis of results	14	Describe the methods of handling data and combining results of studies, if done, including measures of consistency (e.g., I^2) for each meta-analysis.	Page

For peer review only - http://bmjopen.bmj.com/site/about/guidelines.xhtml

PRISMA 2009 Checklist

			11-12

Section/topic	#	Checklist item	Reported on page #
Risk of bias across studies	15	Specify any assessment of risk of bias that may affect the cumulative evidence (e.g., publication bias, selective reporting within studies).	NA
Additional analyses	16	Describe methods of additional analyses (e.g., sensitivity or subgroup analyses, meta-regression), if done, indicating which were pre-specified.	NA
**RESULTS**			
Study selection	17	Give numbers of studies screened, assessed for eligibility, and included in the review, with reasons for exclusions at each stage, ideally with a flow diagram.	Page 13
Study characteristics	18	For each study, present characteristics for which data were extracted (e.g., study size, PICOS, follow-up period) and provide the citations.	Page 13-14
Risk of bias within studies	19	Present data on risk of bias of each study and, if available, any outcome level assessment (see item 12).	Page 14
Results of individual studies	20	For all outcomes considered (benefits or harms), present, for each study: (a) simple summary data for each intervention group (b) effect estimates and confidence intervals, ideally with a forest plot.	NA
Synthesis of results	21	Present results of each meta-analysis done, including confidence intervals and measures of consistency.	Page 14-20
Risk of bias across studies	22	Present results of any assessment of risk of bias across studies (see Item 15).	NA
Additional analysis	23	Give results of additional analyses, if done (e.g., sensitivity or subgroup analyses, meta-regression [see Item 16]).	NA
**DISCUSSION**			
Summary of evidence	24	Summarize the main findings including the strength of evidence for each main outcome; consider their relevance to key groups (e.g., healthcare providers, users, and policy makers).	Page 21-25
Limitations	25	Discuss limitations at study and outcome level (e.g., risk of bias), and at review-level (e.g., incomplete retrieval of identified research, reporting bias).	Page 25-26
Conclusions	26	Provide a general interpretation of the results in the context of other evidence, and implications for future research.	Page 26
**FUNDING**			
Funding	27	Describe sources of funding for the systematic review and other support (e.g., supply of data); role of funders for the systematic review.	Page 2

From: Moher D, Liberati A, Tetzlaff J, Altman DG, The PRISMA Group (2009). Preferred Reporting Items for Systematic Reviews and Meta-Analyses: The PRISMA Statement. PLoS Med 6(7): e1000097.

For peer review only - http://bmjopen.bmj.com/site/about/guidelines.xhtml

PRISMA 2009 Checklist

doi:10.1371/journal.pmed1000097

For more information, visit: **www.prisma-statement.org**.

For peer review only

Supplementary.Material.2 The detailed searching strategy

*PubMed:

(“Carcinoma, non-small cell lung” OR “Lung carcinoma, non-small-cell” OR “Carcinoma, non-small cell lung” OR “Non-small cell lung cancer”)

AND (“adjuvant chemotherapy” OR “chemotherapy, adjuvant”)

AND (“platinum” OR “cisplatin” OR “carboplatin” or “nedaplatin” or “oxaliplatin”)

*EMBASE, and The Cochrane Library, Web of Science and Scopus Google Scholar:

(“Carcinoma, non-small cell lung” OR “Lung carcinoma, non-small-cell” OR “Carcinoma, non-small cell lung” OR “Non-small cell lung cancer”)

AND (“postoperative adjuvant chemotherapy”)

AND (“platinum” OR “cisplatin” OR “carboplatin” or “nedaplatin” or “oxaliplatin”)

In addition, searching performed in the database of Web of Science and Scopus Google Scholar was constrained to titles and abstracts while no limitations were applied in the PubMed, EMBASE, and The Cochrane Library.

For peer review only
